# Risks in the analogue and digitally-supported medication process and potential solutions to increase patient safety in the hospital: A mixed methods study

**DOI:** 10.1371/journal.pone.0297491

**Published:** 2024-02-27

**Authors:** Julia Kopanz, Katharina Lichtenegger, Christine Schwarz, Melanie Wimmer, Lars Peter Kamolz, Thomas Pieber, Gerald Sendlhofer, Julia Mader, Magdalena Hoffmann

**Affiliations:** 1 Department of Internal Medicine, Division of Endocrinology and Diabetology, Medical University of Graz, Styria, Austria; 2 Department of Quality and Risk Management, University Hospital of Graz, Styria, Austria; 3 Department for Surgery, c/o Division for Plastic, Aesthetic and Reconstructive Surgery, Research Unit for Safety and Sustainability in Healthcare, Medical University of Graz, Styria, Austria; University of Copenhagen: Kobenhavns Universitet, DENMARK

## Abstract

**Background:**

In hospital medication errors are common. Our aim was to investigate risks of the analogue and digitally-supported medication process and any potential solutions.

**Methods:**

A mixed methods study including a structured literature search and online questionnaires based on the Delphi method was conducted. First, all risks were structured into main and sub-risks and second, risks were grouped into risk clusters. Third, healthcare experts assessed risk clusters regarding their likelihood of occurrence their possible impact on patient safety. Experts were also asked to estimate the potential for digital solutions and solutions that strengthen the competence of healthcare professionals.

**Results:**

Overall, 160 main risks and 542 sub-risks were identified. Main risks were grouped into 43 risk clusters. 33 healthcare experts (56% female, 50% with >20 years professional-experience) ranked the likelihood of occurrence and the impact on patient safety in the top 15 risk clusters regarding the process steps: admission (n = 4), prescribing (n = 3), verifying (n = 1), preparing/dispensing (n = 3), administering (n = 1), discharge (n = 1), healthcare professional competence (n = 1), and patient adherence (n = 1). 28 healthcare experts (64% female, 43% with >20 years professional-experience) mostly suggested awareness building and training, strengthened networking, and involvement of pharmacists at point-of-care as likely solutions to strengthen healthcare professional competence. For digital solutions they primarily suggested a digital medication list, digital warning systems, barcode-technology, and digital support in integrated care.

**Conclusions:**

The medication process holds a multitude of potential risks, in both the analogue and the digital medication process. Different solutions to strengthen healthcare professional competence and in the area of digitalization were identified that could help increase patient safety and minimize possible errors.

## Introduction

Medication errors cause about 1–2% of complications in inpatients [1]. Annually, around 7,000 patients worldwide die due to illegible writing [2], and it can be assumed that this is only the tip of the iceberg, as medication errors and their effects often remain undetected [3].

Factors that contribute to medication errors are diverse [4, 5] and occur in all phases of the medication process, including handwritten prescription (from 7% to 49% error rate), transcription of medication to another document (11% error rate), dosing/dispensing (14% error rate) and administration (26% error rate) [4, 6]. Similarly, an Australian literature review described that at hospital admission two errors per three patients occurred, and in the prescription process up to one error per patient was found [7]. Errors occurred in 9% of administrations and in the discharge documentation up to two errors per patient were found. Factors that contribute to errors include a lack of training or experience, fatigue, stress, high workload, insufficient knowledge or a lack of interest on the part of the prescriber or the nursing staff [8].

The majority of digitally-supported medication processes are still partially organized in an analogue way [9–12]. An analogue medication process is defined as a process that still includes work steps and processes that do not use digital tools. For example, a process is initiated with a digital prescription tool, but the medication is then dispensed and distributed manually without further digital tools. The goal is a completely digital process which is not only initialized with a digital prescription tool but also followed by machine-supported medication dispensing and secure, barcode-supported medication distribution to patients. The completely digital process aims to minimize or even prevent medication errors by ensuring that the right medication is prescribed, dispensed, and distributed to the right patient [9–12].

This study focused on investigating risks in the analogue and digitally-supported medication process and potential solutions in a hospital setting. Thus, the long-term goal is to investigate possibilities, challenges, and competence needs in order to introduce a digitally-supported medication process to increase patient safety in hospitals and intersectoral interfaces, respectively as well as healthcare professional safety.

## Patients, materials and methods

### Study design and setting

**Fig 1:**
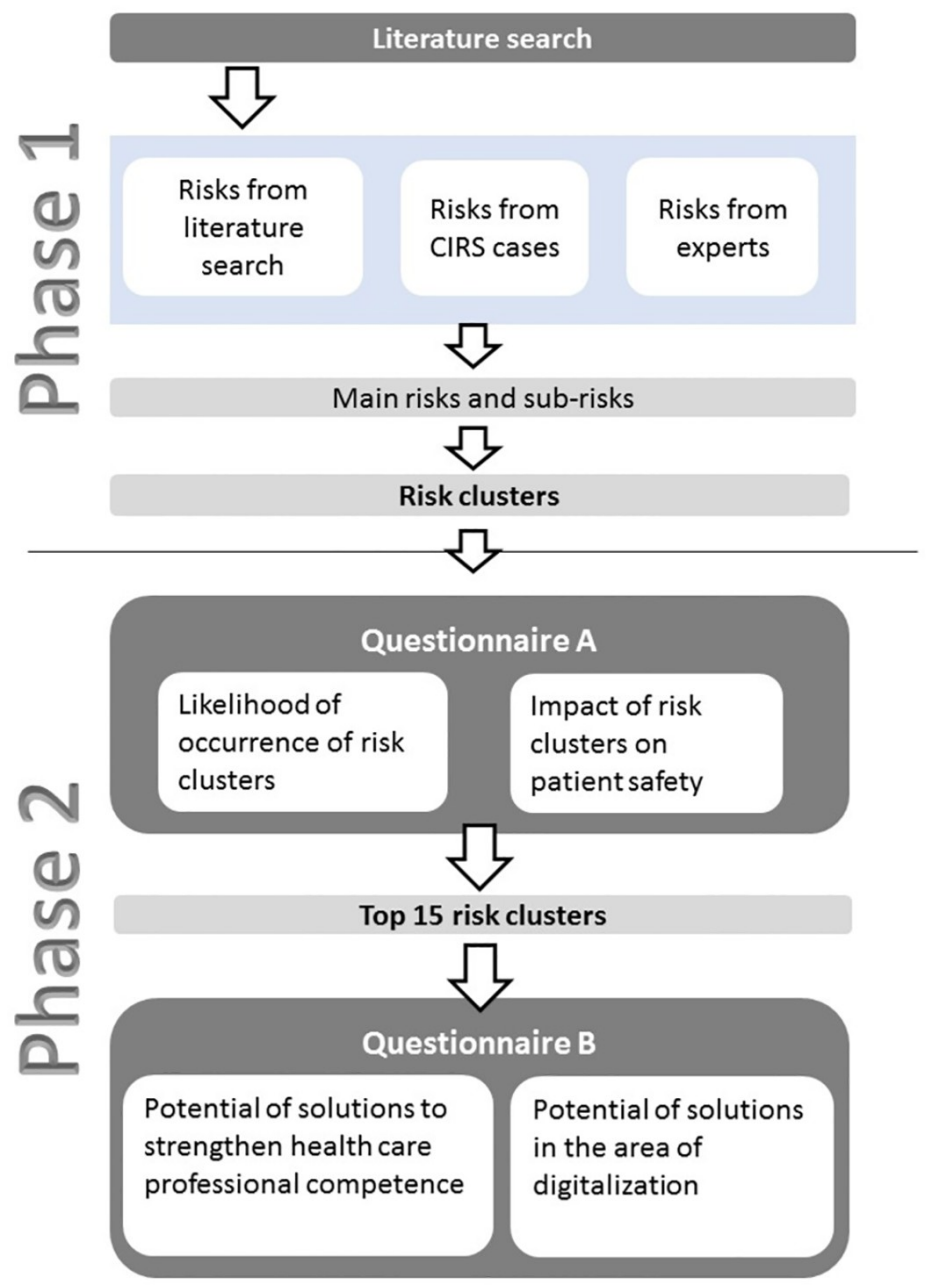
Procedure of the mixed method study with literature search and questionnaires based on the Delphi method.

This was a mixed methods study including a structured literature search and online questionnaires based on the Delphi method (Fig 1). The study was approved by the ethics committee of the Medical University of Graz (No. 32–498 ex 19/20) and was designed, conducted, performed, and analyzed in accordance with Good Clinical Practice, with the principles of the “Declaration of Helsinki”, with the laws and regulations of the participating European country.

### Phase 1—Identification of main risk clusters

#### Literature search

A structured literature search using a hermeneutic approach was performed to identify risks in the analogue and digitally-supported medication process in a hospital setting.

The database PubMed was searched for relevant literature between February and March 2020 to answer the research question: “What are the major risks in the hospital medication use process?”. We used the berrypicking approach [13] and also included previously identified literature when it provided important knowledge. In doing so, search terms were created to identify further significant literature [13]. The following main categories of search terms were used: risk, quality indicator, medication process, medication error, hospital, and digital. We defined keywords and MeSH terms for these main categories and combined them using the Boolean operators “AND” and “OR”. We limited the search to systematic reviews in English. Reviews concerning only the setting critically ill patients, pediatrics, emergency department, or outpatient clinic were excluded. Systematic reviews that included studies in different settings (e.g., general inpatient ward and pediatric wards and/or intensive care units) and that investigated the whole medication process and thus provided relevant information to answer the research question were included. To ensure current data we only searched for reviews published between 2010 and 2020. Results were screened regarding title and abstract. We identified a total of 40 relevant systematic reviews and chose 15 after full text screening. In these 15 systematic reviews we identified risks and main outcomes.

#### Grouping to risk clusters

Identified risks from the literature were supplemented with risks from the Critical Incident Reporting System (CIRS) reports from the University Hospital of Graz and with risks identified by seven health care professionals. All CIRS cases between 2013 and 2020 were screened and risks related to the medication process were extracted. In addition, categorized "near misses" and "no CIRS cases" with risks relevant to the medication process were also extracted. All risks, including risks from the literature, risks from CIRS reports, and risks from healthcare professionals were combined into one list and structured into main risks and sub-risks of the medication process. Subsequently, two investigators independently grouped the main risks into risk cluster to identify the most relevant risks. The risk clusters were matched and in case of discrepancies a third researcher was consulted.

### Phase 2—Likelihood of risk occurrence, impact on patient safety, and potential for solutions

#### Questionnaires

Two online questionnaires (A, B) based on the Delphi method [14] were conducted to assess the likelihood of occurrence, the impact on patient safety, the potential for solutions for the identified risk clusters. Questionnaire A aimed to answer the research questions: “How likely will this risk cluster occur and what is the estimated impact of this risk cluster on patient safety in a hospital?”. Questionnaire B aimed to answer the research questions: “What is the potential for solutions to strengthen the competence of healthcare professionals and what is the potential of finding digital solutions in the hospital?”. In total, 75 healthcare experts from the DACH region (Germany, Austria, Switzerland) from different clinical and patient-related fields were asked to fill-in both questionnaires.

*Questionnaire A*. (S1 File) was sent out in July 2020 and August 2020. We asked experts to evaluate the risk clusters concerning the likelihood of occurrence and the impact on patient safety on a Likert scale ranging from 1–10. For both parameters, 10 represented the highest likelihood of occurrence and a fatal impact on patient safety, whereas 1 represented a very low likelihood of occurrence and a low impact on patient safety. Experts were also asked to identify the greatest risk in each risk cluster, regardless of how they rated other individual risks in this cluster. Results were ranked and for the top 15 risk clusters a further risk assessment was conducted and investigation on potential for solutions was assessed by adding the likelihood of occurrence (mean) and the impact on patient safety of each risk cluster (mean). Finally, these results were divided into two and were further ranked starting with the highest rating.

*Questionnaire B*. Questionnaire B (S2 File) (October 2020 –November 2020) was based on the top 15 risk clusters. The potential for solutions to strengthen the competence of healthcare professionals and the potential for digital solutions were analysed. We asked the same experts to evaluate the top 15 risk clusters on a Likert scale ranging from 1–10, with 10 representing a very high potential for solutions and 1 representing a very low potential for solutions. For each risk cluster, experts also had the option to give a specific suggestion for a solution by adding a comment.

### Data management and statistical analysis

Data gathered from the online questionnaires were collected and pseudo-anonymized so that no conclusions could be drawn about participants. All collected data were automatically imported into the data management software EvaSys (Electric Paper. EvaSys 8.1, Electric Paper Evaluationssysteme GmbH, Lüneburg. Deutschland, 2013), and data were checked for completeness, and plausibility. The statistical analysis was carried out using descriptive statistics by using the software EvaSys. For numerical data, mean values, and standard deviations were calculated. Categorical data are presented as absolute and relative frequencies.

## Results

### Phase 1—Risk clusters of the hospital medication process

**Fig 2:**
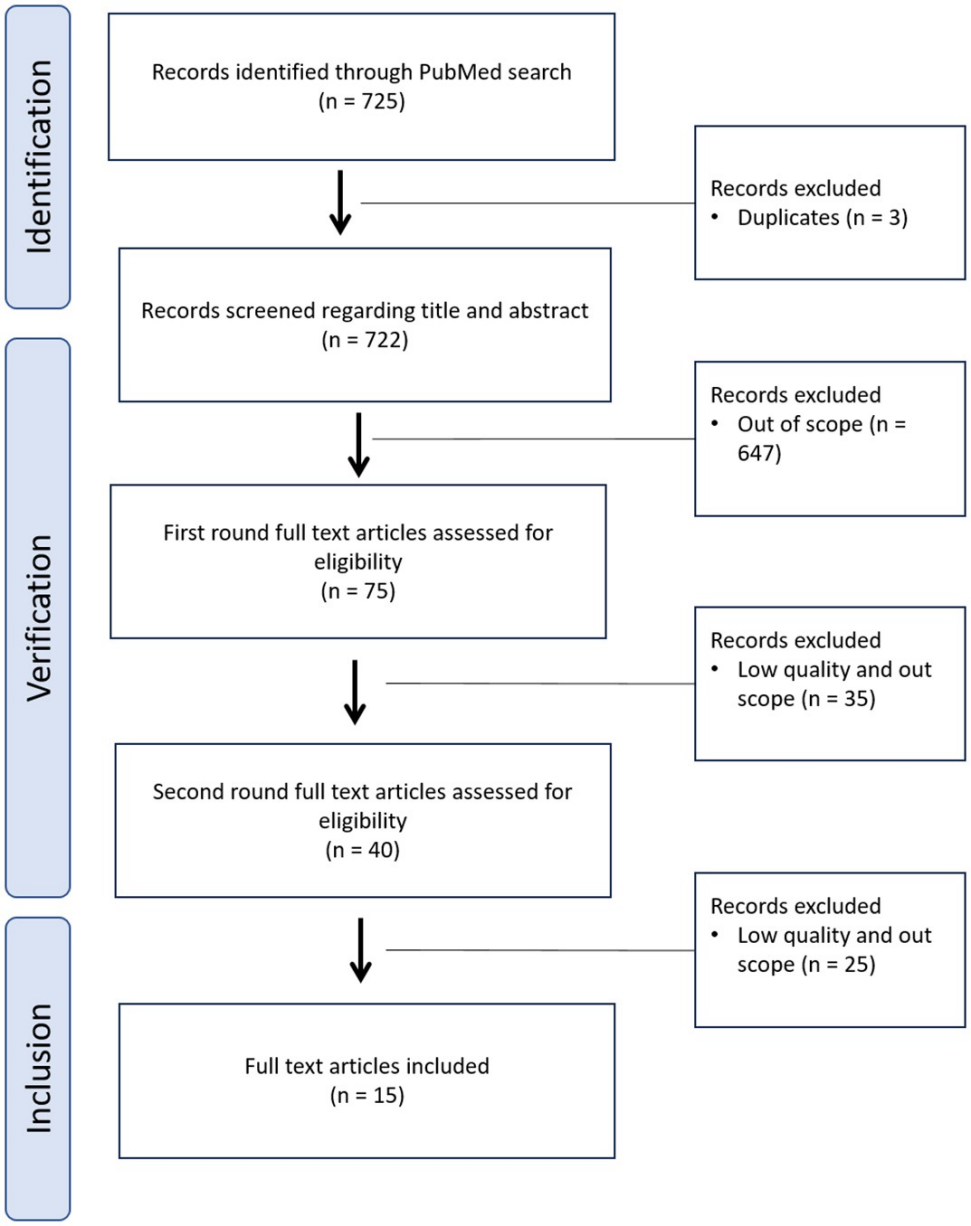
Flow chart of literature search process.

In total, 15 systematic reviews were included [15–29] (see Table 1). Details on the structured literature search process are presented in Fig 2.

**Table 1:** Characteristics of included systematic reviews to identify risks in the medication process in the hospital.

Reference, year of publication, country	Population Setting	Aim	Steps of the medication process to which risks were structured
Alanazi MA, Tully MP, Lewis PJ., 2016, UK [15]	**Population**: children and adults **Setting**: hospital	To review the literature in order to report on the prevalence and incidence of prescribing errors in high-risk medicines in hospital inpatients	Prescribing
Berdot S, Gillaizeau F, Caruba T, Prognon P, Durieux P, Sabatier B., 2013, France [16]	**Population**: patients **Setting**: hospital	To analyze the published evidence concerning the prevalence, nature and severity of administration errors in hospitals detected by the observation technique	Preparing/Dispensing Administering
Bos JM, van den Bemt PMLA, de Smet PAGM, Kramers C., 2017, The Netherlands [17]	**Population**: different physicians in different settings (intensive care, surgical, internal, pediatric, geriatric wards, surgical and medical house staff, trainees) **Setting**: hospital	To focus on the existing literature on the education of prescribers in hospitals reporting outcomes of (potential) patient harm and to address the scope and form of the education programs described in the literature and give an appraisal of the scientific merits of the individual studies	Prescribing
Dalton K, O’Brien G, O’Mahony D, Byrne S., 2018, Ireland [18]	**Population**: prescribers in settings with computerized provider order entry (CPOE) **Setting**: hospital (teaching hospital, medical ward, acute geriatric ward in academic urban hospital, urban teaching hospital, medical, surgical, neurology, gynecology services in tertiary care, emergency unit in a university affiliated, urban, public hospital)	To examine the evidence for efficacy of computerized interventions designed to reduce potentially inappropriate prescribing in this patient group	Prescribing Patient Digital process and IT-security Organization
Hedlund N, Beer I, Hoppe-Tichy T, Trbovich P., 2017, USA, Germany, Canada [19]	**Population**: pediatric and adult patients **Setting**: in the hospital or any other institutional or outpatient healthcare setting associated with a hospital. (critical care, general inpatient wards, pediatric units, oncology or hematology, obstetrics)	To identify the incidence of intravenous admixture preparation errors (IAPEs) (overall and by subtype) reported across institutional healthcare settings and to understand the frequency of error subtypes and associated burden of patient harm attributable to IAPEs as reported in the published literature	Preparing/Dispensing Administering
Hias J, Van der Linden L, Spriet I, Vanbrabant P, Willems L, Tournoy J, De Winter S., 2017, Belgium [20]	**Population**: adult patients **Setting**: admission to hospital	To identify predictors of medication discrepancies and to identify specific variables that are aligned with the highest risk for unintentional discrepancies in medication histories	Admission Discharge
Keers RN, Williams SD, Cooke J, Ashcroft DM., 2013, UK [21]	**Population**: nurses and physicians **Setting**: teaching hospitals, general and unspecific hospitals, tertiary care hospitals, army medical center, pediatrics hospital as well as unknown institution	To review and appraise empirical evidence relating to the causes of medication administration errors (MAEs) in hospital settings	Administering Monitoring Healthcare professional competence Patient Organization
Keers RN, Williams SD, Cooke J, Walsh T, Ashcroft DM., 2014, UK [22]	**Population**: medical staff **Setting**: medical, surgical, intensive care units, step-down units, operating theatre and geriatric assessment and rehabilitation.	To review and appraise interventions designed to reduce MAEs in the hospital setting	Administering Monitoring Healthcare professional competence Patient
Korb-Savoldelli V, Boussadi A, Durieux P, Sabatier B., 2018, France [23]	**Population**: prescribers **Setting**: hospitals (teaching hospital, community hospital, pediatric teaching hospital)	To analyze the prevalence of prescription errors related to the use of CPOE systems.	Prescribing Preparing/Dispensing Administering Healthcare professionals’ competence Digital process and IT-security
Larmené-Beld KHM, Alting EK, Taxis K., 2018, The Netherlands [24]	**Population**: healthcare professionals, non-healthcare professionals. **Setting**: laboratory and healthcare setting	To evaluate the current evidence on strategies to minimize medication errors due to look-alike labels	Preparing/Dispensing
Nuckols TK, Smith-Spangler C, Morton SC, Asch SM, Patel VM, Anderson LJ, Deichsel EL, Shekelle PG., 2014, USA [25]	**Population**: prescribers in hospital settings **Setting**: adult medical or surgical wards, adult medical or surgical intensive care units, emergency departments, or the entire hospital	To assess the effectiveness of CPOE at reducing preventable adverse drug events (pADEs) in hospital-related settings and to examine reasons for heterogeneous effects on medication errors	Admission Preparing/Dispensing Administering
Page N, Baysari MT, Westbrook JI., 2017, Australia [26]	**Population**: prescriber **Setting**: university hospitals, tertiary hospitals, academic medical centers and specialty transplant hospital	To assess the evidence of the effectiveness of different categories of interruptive medication prescribing alerts to change prescriber behavior and/or improve patient outcomes in hospital CPOE systems	Admission Prescribing Healthcare professional competence Digital process and IT-security
Redmond P, Grimes TC, McDonnell R, Boland F, Hughes C, Fahey T., 2018, Ireland and UK [27]	**Population**: all patients (aged > 18 years) at a transition of care **Setting**: hospitals, primary care practices, long-term care facilities	To assess the effect of medication reconciliation on medication discrepancies, patient-related outcomes and healthcare utilization in people receiving this intervention during care transitions compared to people not receiving medication reconciliation	Prescribing Administering
Smeulers M, Verweij L, Maaskant JM, de Boer M, Krediet CT, Nieveen van Dijkum EJ, Vermeulen H., 2015, The Netherlands [28]	**Population**: nursing indicators, patient safety indicators, indicators for quality use in medicines, medication safety indicators **Setting:** acute care setting, hospital, inpatient and outpatient setting	To identify evidence-based quality indicators (structure, process and outcome) for the 7 rights of safe in-hospital medication preparation and administration	Prescribing Verifying Preparing/Dispensing Administering Monitoring Healthcare professional competence Organization
Vélez-Díaz-Pallarés M, Pérez-Menéndez-Conde C, Bermejo-Vicedo T., 2018, Spain [29]	**Population**: Hospitalized patients **Setting:** hospital	To evaluate the effect of CPOE with clinical decision support on medication error (ME) and to present adverse drug event (ADE) rates	Prescribing Administering Digital process and IT-security

**Fig 3:**
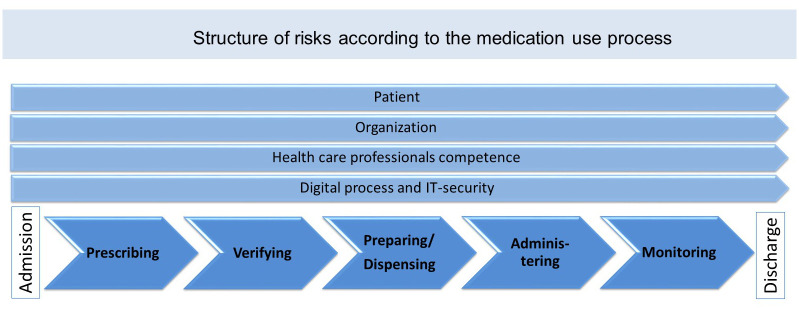
Structure of risks identified in the literature search according to the in-hospital medication process.

All risks were structured in relation to the following steps in the medication process: admission, prescribing, verifying, preparing/dispensing, administering, monitoring, and discharge. Additionally, we defined additional steps that are also important in a comprehensive medication process: patient adherence, organization, healthcare professional competence, digital process, IT-security (Fig 3).

In total we identified 160 main risks and 542 sub-risks from literature, CIRS reports, and healthcare experts. Main risks were grouped into 43 risk clusters (see S1 Appendix). Risk clusters were built for all defined steps of the medication process, except for “organization” which was excluded because it is related to organizational issues only.

### Burden and potential solutions for 15 top risk clusters

#### Questionnaire A (S1 File)

In total, 33 out of 75 experts (56% female, 50% with > 20 years of professional experience, profession: 32% nurses, 29% physicians, 23% quality management/risk management, 7% management, and 10% others) evaluated 43 risk clusters (response rate: 44%). Table 2 provides an overview of results ranked by priorities. The top 15 risk clusters comprise the following steps of the medication process: admission (n = 4), prescribing (n = 3), verifying (n = 1), preparing/dispensing (n = 3), administering (n = 1), discharge (n = 1), healthcare professional competence (n = 1), patient adherence (n = 1). The results for all 43 risk clusters are shown in the S1 Appendix.

**Table 2:** Results from questionnaire A (S1 File): Likelihood of occurrence and impact on patient safety of the 15 top risk clusters (ranked by priority starting with highest priority).

No.	Risk cluster	Likelihood of occurrence, impact on patient safety	Mean ± SD; (N)
**1**	**Admission**: Challenge with medications (polypharmacy defined as more than 5 drugs, generic drugs vs. original drugs, high-risk drugs, drug-drug interactions).	Likelihood of occurrence	7.7 ± 2.1; (33)
		Impact on patient safety	7.4 ± 2.0; (33)
**2**	**Prescribing**: Difficulties with handwritten prescription (e.g., incomplete prescription, illegibility of prescription, prescription written in pencil or “non-waterproof pen“, use of correction varnish).	Likelihood of occurrence	7.7 ± 2.2; (33)
		Impact on patient safety	6.9 ± 2.5; (33)
**3**	**Prescribing**: General prescription errors (e.g., wrong drug, wrong dose, incomplete prescription, other types of errors such as omission errors, transcription errors, duplication errors).	Likelihood of occurrence	7.1 ± 2.2; (33)
		Impact on patient safety	7.3 ± 1.8; (33)
**4**	**Healthcare professional competence**: Problematic environment during each step of the medication process (e.g., noise, poor lighting, emergencies, chaotic work environment, interruption/distraction and heavy staff workload due to e.g., understaffing, poor ward equipment).	Likelihood of occurrence	6.6 ± 3.0; (33)
		Impact on patient safety	6.9 ± 2.6; (33)
**5**	**Admission**: Allergy errors (allergies are not recorded, not or incorrectly documented, or not considered, no information about allergies provided by patients).	Likelihood of occurrence	5.9 ± 2.5; (32)
		Impact on patient safety	7.4 ± 2.1; (33)
**6**	**Admission**: Incomplete medication list at admission with discrepancies in medication histories (e.g., different lists from patient, general practitioner, specialist, electronic medication record).	Likelihood of occurrence	7.1 ± 2.0; (33)
		Impact on patient safety	6.1 ± 1.9; (33)
**7**	**Prescribing**: Challenges in the prescription of complex/high risk medications (e.g., polypharmacy, lack of control of drug-drug interactions) due to lack of clinical pharmacological knowledge (e.g., irrational, inappropriate, ineffective prescribing) and/or due to lack of prescribing regimens or non-use of existing prescribing regimens.	Likelihood of occurrence	6.0 ± 2.3; (33)
		Impact on patient safety	7.0 ± 2.0; (33)
**8**	**Administering**: Errors in administering medications (e.g., wrong drug, wrong dosage, wrong route of administration, confusion of look-a-like or sound-a-like medications, wrong time of administration, unauthorized drugs, omission errors, faulty checking activities, difficulties with infusion equipment, confusion of packaging of medications, incorrect labeling of medication on packaging).	Likelihood of occurrence	5.2 ± 2.5; (33)
		Impact on patient safety	7.5 ± 2.2; (33)
**9**	**Preparing/Dispensing**: Lack of communication/misunderstandings in communication (e.g., errors in telephone orders, misunderstanding regarding drug name, dosage, interval, type of dosage, patient).	Likelihood of occurrence	6.1 ± 2.5; (32)
		Impact on patient safety	6.7 ± 2.5; (32)
**10**	**Admission**: Inadequate communication about prescribed medications among community care services and hospital.	Likelihood of occurrence	6.6 ± 2.2; (32)
		Impact on patient safety	6.2 ± 1.8; (32)
**11**	**Discharge**: Missing communication/information with patients and relatives (e.g., medication needs, medications are not explained).	Likelihood of occurrence	6.3 ± 2.6; (32)
		Impact on patient safety	6.4 ± 2.1; (32)
**12**	**Preparing/Dispensing:** Errors in preparing/dispensing medications (e.g., errors in splitting tablets, wrong drug, wrong dose, wrong drug calculation, missing or incorrect change of ordered medication in the dispenser, missing or incorrect documentation, missing/incorrect/unclear labeling/marking of prepared medications).	Likelihood of occurrence	5.9 ± 2.6; (33)
		Impact on patient safety	6.7 ± 2.3; (33)
**13**	**Patient:** Risk factors related to patients (e.g., lack of compliance, lack of health literacy, lack of knowledge about their own medications, wrong intake of medications).	Likelihood of occurrence	6.1 ± 2.0; (32)
		Impact on patient safety	6.6 ± 2.1; (32)
**14**	**Verifying**: Missing verification/support for complex prescriptions by (clinical) pharmacists (e.g., high-risk medications, polypharmacy, complex indications and diagnoses).	Likelihood of occurrence	6.1 ± 3.0; (33)
		Impact on patient safety	6.3 ± 2.5; (33)
**15**	**Preparing/Dispensing:** Confusion of medications (e.g., look-a-like medication error, sound-a-like medication error, confusion of medication names/packages).	Likelihood of occurrence	5.4 ± 2.6; (33)
		Impact on patient safety	7.0 ± 2.4; (32)

Mean ± SD (standard deviation); N = number of responses from experts: Likert scale 1–10 (1 = very low likelihood of occurrence / impact on patient safety; 10 = very high likelihood of occurrence / impact on patient safety).

#### Questionnaire B (S2 File)

28 (response rate: 37%) out of 75 experts (64% female, 43% with > 20 years of professional experience, profession: 32% nurses, 29% physicians, 25% quality management/risk management, 7% management and 7% other) evaluated the top 15 risk clusters regarding their potential for solutions to strengthen the competence of healthcare professionals and the potential for digital solutions (Table 3). Experts suggested different solutions: awareness and training for healthcare professionals, more networking, and involvement of pharmacists at point of care were the most frequently mentioned solutions to strengthen competences. As a digital solution experts mostly suggested a digital medication list, digital warning systems, use of barcode technology, and digital support in integrated care.

**Table 3:** Results from questionnaire B (S2 File): Potential for solutions to strengthen healthcare professional competence and the potential for digital solutions of the top 15 risk clusters.

No	Risk cluster	Potential of a solution	Mean ± SD; (N)	Suggested solutions from experts
**1**	**Admission**: Challenge with medications (polypharmacy defined as more than 5 drugs, generic drugs vs. original drugs, high-risk drugs, drug-drug interactions).	Solution to strengthen healthcare professional competence	6.6 ± 2.1; (28)	Verification of medication list if more than 5 drugs Pharmacist at point of care
		Solution in the area of digitalization	7.7 ± 1.5; (28)	Automatic check of interactions with intelligent algorithms Automatic presentation of potential risks (e.g., double prescription, incorrect dosage) Digital warning systems Medication training for healthcare professionals
**2**	**Prescribing**: Difficulties with handwritten prescription (e.g., incomplete prescription, illegibility of prescription, prescription written in pencil or “non-waterproof pen “, use of correction varnish).	Solution to strengthen healthcare professional competence	5.1 ± 2.5; (28)	No specific solution
		Solution in the area of digitalization	9.0 ± 1.7; (28)	Electronic medication record
**3**	**Prescribing**: General prescription errors (e.g., wrong drug, wrong dose, incomplete prescription, other types of errors such as omission errors, transcription errors, duplication errors).	Solution to strengthen healthcare professional competence	7.0 ± 1.7; (28)	No specific solution
		Solution in the area of digitalization	7.9 ± 2.1; (28)	Intelligent software for medication documentation and verification of prescribing by specialist
**4**	**Healthcare professional competence**: Problematic environment during each step of the medication process (e.g., noise, poor lighting, emergencies, chaotic work environment, interruption/distraction and heavy staff workload due to e.g., understaffing, poor ward equipment).	Solution to strengthen healthcare professional competence	7.4 ± 2.5; (28)	Adaption of environment Training and awareness building of healthcare professionals Time for preparing medication without interruptions (e.g., warn reflecting vest during dispensing “Talk to me only in emergency…”) Crisis-resources-management Use of check lists Strengthen of awareness
		Solution in the area of digitalization	4.0 ± 2.2; (28)	No specific solution
**5**	**Admission**: Allergy errors (allergies are not recorded, not or incorrectly documented or not considered, no information about allergies provided by patients).	Solution to strengthen healthcare professional competence	6.6 ± 2.5; (28)	Training of healthcare professionals
		Solution in the area of digitalization	7.8 ± 2.1; (28)	Allergy list available via digital medication list of family doctor Verification of allergies obligatory Digital alert system Central data entry (e.g., family doctor) and verification of medication
**6**	**Admission**: Incomplete medication list at admission with discrepancies in medication histories (e.g., different lists from patient, General Practitioner, specialist, electronic medication record).	Solution to strengthen healthcare professional competence	5.1 ± 2.5; (28)	Digital medication list
		Solution in the area of digitalization	7.9 ± 1.6; (28)	Digital medication list with date of expiry Medication list with QR-Code to transfer data in hospital information system and vice versa
**7**	**Prescribing**: Challenges in the prescription of complex/high risk medications (e.g., polypharmacy, lack of control of drug-drug interactions) due to lack of clinical pharmacological knowledge (e.g., irrational, inappropriate, ineffective prescribing) and/or due to lack of prescribing regimens or non-use of existing prescribing regimens.	Solution to strengthen healthcare professional competence	7.1 ± 1.9; (28)	Pharmacist available for interactive communication Information available via digital medication list Integrated patient care (not only one discipline)
		Solution in the area of digitalization	8.0 ± 1.6; (28)	Digital medication list with intelligent alarming (medical expertise for off label use of certain drugs are more important than software solution)
**8**	**Administering**: Errors in administering medications (e.g., wrong drug, wrong dosage, wrong route of administration, confusion of look-a-like or sound-a-like medications, wrong time of administration, unauthorized drugs, omission errors, faulty checking activities, difficulties with infusion equipment, confusion of packaging of medications, incorrect labeling of medication on packaging).	Solution to strengthen healthcare professional competence	7.8 ± 1.9; (28)	Awareness building of healthcare professionals
		Solution in the area of digitalization	5.8 ± 2.0; (28)	Approval/check-out with digital medication list Digital alarm for,, digital”overdosing Barcode-scan for alignment of right patient and right medication in right timing Special presentation of new prescribed medication
**9**	**Preparing/Dispensing**: Lack of communication/misunderstandings in communication (e.g., errors in telephone orders, misunderstanding regarding drug name, dosage, interval, type of dosage, patient).	Solution to strengthen healthcare professional competence	7.1 ± 2.2; (28)	Awareness training of healthcare professionals Approval of medication by treating physician
		Solution in the area of digitalization	6.6 ± 1.9; (28)	No specific solution
**10**	**Admission**: Inadequate communication about prescribed medications between community care services and hospital.	Solution to strengthen healthcare professional competence	6.0 ± 2.6; (28)	Awareness building of healthcare professionals for communication with external sectors Strengthen networking
		Solution in the area of digitalization	7.9 ± 1.4; (28)	Digital medication list filled-in by general practitioner Identification of possible risk factors Support of integrated care (general practitioner—hospital)
**11**	**Discharge**: Missing communication/information with patients and relatives (e.g., medication needs, medications are not explained).	Solution to strengthen healthcare professional competence	8.0 ± 1.8; (28)	No specific solution
		Solution in the area of digitalization	4.5 ± 2.0; (28)	Digital medication list used in all healthcare settings Transparent presentation of changes in medication list Medication app for patient education
**12**	**Preparing/Dispensing:** Errors in preparing/dispensing medications (e.g., errors in dividing tablets, wrong drug, wrong dose, wrong drug calculation, missing or incorrect change of ordered medication in the dispenser, missing or incorrect documentation, missing/incorrect/unclear labeling/marking of prepared medications).	Solution to strengthen healthcare professional competence	7.9 ± 1.2; (28)	4-eye-principle Silent environment Outsourcing of dispensing process (to reduce errors, more time for patient care) Awareness building of healthcare professionals
		Solution in the area of digitalization	6.2 ± 1.8; (28)	Readable digital medication list Digital help for error reduction
**13**	**Patient**: Risk factors related to patients (e.g., lack of compliance, lack of health literacy, lack of knowledge about their own medications, wrong intake of medications).	Solution to strengthen healthcare professional competence	7.6 ± 1.9; (28)	Educational work Information sheets for patients in understandable language Medication lists printed out for wallet Communication training healthcare professional-patient Relationship with patient (empathy, patient will) Healthcare professionals training in patient education (e.g., meaningful conversation techniques such as motivational interviewing)
		Solution in the area of digitalization	4.4 ± 2.2; (28)	App for special medication groups
**14**	**Verifying**: Missing verification/support for complex prescriptions by (clinical) pharmacists (e.g., high-risk medications, polypharmacy, complex indications and diagnoses).	Solution to strengthen healthcare professional competence	7.1 ± 2.1; (28)	No specific solution
		Solution in the area of digitalization	7.4 ± 2.0; (28)	Automatic message in digital record for patients with more than 5 drugs if pharmaceutical check is needed Definition of red flags
**15**	**Preparing/Dispensing**: Confusion of medications (e.g., look-a-like medication error, sound-a-like medication error, confusion of medication names/packages).	Solution to strengthen healthcare professional competence	7.5 ± 2.3; (28)	Outsourcing of dispensing process Reduce look-a-like medication in use Training for healthcare professionals CIRS reporting Awareness building of healthcare professionals
		Solution in the area of digitalization	6.2 ± 2.5; (28)	Dispensing with barcode reader to reduce mistakes

Mean ± SD (standard deviation); N = number of experts: Likert scale 1–10 (1 = very low likelihood of solutions; 10 = very high likelihood of solutions)

## Discussion

This study aimed to investigate the risks of the analogue and digitally-supported medication process and the likelihood of solutions in a hospital setting. The study identified 160 main risks and 542 sub-risks regarding the medication process using literature search, review of CIRS cases, and experts’ opinions. Results underline the complexity of the medication process and its high potential for risks and consequently resulting possible medication errors in all steps [4, 6, 7, 30].

All identified risks were grouped into 43 risk clusters. According to the participating experts the greatest burden relates to the main steps in the medication process such as admission, prescribing, verifying, preparing/dispensing, administering, discharge, healthcare professional competence, and patient. Most critical risks occur at admission, in prescribing, and during preparing/dispensing of medications. Similar to our results, Roughead et al. [7] found error occurrences at admission and in medication prescribing, but they also indicated error rates in other steps of the medication process such as medication administration and discharge. A review of medication incidents revealed that administration (50%) and prescription (18%) are most critical [31]. Similar results have been found in another study, where 68% of medication errors were related to administration and 24% to prescribing based on the incident reports in Norwegian hospitals [32]. Comparability of different studies is limited as different definitions for medication errors were used and standard definitions and thus standard data collection methods for medication errors are missing [15, 16, 19, 33, 34].

To minimize the burden of risks and potential errors, experts mostly suggested the following solutions to strengthen the competence of healthcare professionals: awareness building and additional training for healthcare professionals, strengthened networking, and involvement of pharmacists at the point of care. The highest likelihood of strengthening the competence of healthcare professionals was assessed by experts in the risk cluster”missing communication/information with patients and relatives at discharge”. With regard to communication and discharge planning, a “Guide to Patient and Family Engagement in Hospital Quality and Safety” by the US Agency for Healthcare Research and Quality (AHRQ) advises healthcare professionals to engage patients and families to promote improvement in care [35]. An alternative possibility, as suggested by the American Society of Health System Pharmacists (ASHP) guideline, is the involvement of pharmacists in the discharge process who are competent in key skills, such as patient education, active listening, and interpretation of non-verbal communication [36].

With regard to digital solutions, experts primarily suggested the following: digital medication list [37], digital warning systems, barcode technology [38], and digital support in integrated care [39, 40]. Experts indicated a very high potential for digital solutions in the risk cluster “difficulties with handwritten prescription” and suggested a digital medication list as a specific solution. In a study that compared handwritten prescriptions with digital electronic prescriptions a significant decrease in the incidence of medication errors was detected when digital electronic prescription was used [41]. However, errors can also occur in the digital medication process and must be taken into account. A crucial factor during digitalization is the competence of healthcare professionals. Continuously updated knowledge and skills are essential when using digital technologies but personal attitude can have a major influence and must thus be taken into account [24, 42–47]. It is of major importance for hospitals to follow international recommendations and to enable healthcare professionals to successfully develop, select, implement, or work with digital solutions [48].

This study is limited by focusing on the hospital setting and not reflecting the medication process in other settings. However, the finding from our study can be transferred to other settings (e.g., nursing homes, home healthcare) which are also affected by risks in the medication process, their likelihood of occurrence, the impact on patient safety, and the likelihood of solutions. Another limitation is the targeted expert group that was approached to participate in the two questionnaires. Because all experts are working in the DACH region, a transfer of results to other healthcare system has to be done with caution. Nevertheless, clinical practice can profit from the results by a raised awareness of the risk clusters and the list of possible solutions.

The results of this study form the basis for a larger project that aims to investigate chances, challenges, and needed competences regarding digitally-supported medication processes. In a next step, healthcare professional competence and digital solutions will be investigated in more detail to derive concrete recommendations for intra-hospital clinical practice use.

## Conclusions

The medication process holds a multitude of potential risks, in both the analogue and the digital medication process. Different solutions to strengthen healthcare professional competence and in the area of digitalization were identified that could help increase patient safety and minimize possible errors.

## Supporting information

S1 Appendix 43 risk clusters and their results from questionnaire A (likelihood of occurrence and impact on patient safety). (DOCX)

S1 File Delphi MeDiPro 1. Befragung.
(PDF)

S2 File Delphi MeDiPro 2. Befragung.
(PDF)

S3 File Delphi MeDiPro 1. Survey (English).
(PDF)

S4 File Delphi MeDiPro 2. Survey (English).
(PDF)
