# Supplementary material for: Risks in the analogue and digitally-supported medication process and potential solutions to increase patient safety in the hospital: A mixed methods study
Source: PLoS One. 2024 Feb 27;19(2):e0297491. doi: 10.1371/journal.pone.0297491 (PMC10898776; doi:10.1371/journal.pone.0297491)
Supplement: S1 Appendix — (DOCX) [file pone.0297491.s001.docx]

**Appendix:** 43 risk clusters and their results from Questionnaire A (likelihood of occurrence and impact on patient safety)

| **Steps of the medication use process** | **No.** | **Risk cluster** | **Results from Questionnaire A (Likelihood of occurrence and impact on patient safety)** | **Mean ± SD; (N)** |
| --- | --- | --- | --- | --- |
| **Admission** | 1 | Inadequate communication about prescribed medications among community care services and hospital. | Likelihood of occurrence  Impact on patient safety | 6.6 ±2.2; (32)  6.2 ± 1.8; (32) |
|  | 2 | Incomplete medication list at admission with discrepancies in medication histories (e.g., different lists from patient, general practitioner, specialist, electronic medication record). | Likelihood of occurrence  Impact on patient safety | 7.1 ± 2.0; (33)  6.1 ± 1.9; (33) |
|  | 3 | Patient-related predictors at admission (e.g., lack of compliance, health status, type of admission (acute, elective), or health literacy). | Likelihood of occurrence  Impact on patient safety | 5.9 ± 2.1; (32)  5.4 ± 2.1; (32) |
|  | 4 | Little experience of the admitting physician or lack of support from experienced physicians in the hospital. | Likelihood of occurrence  Impact on patient safety | 5.5 ± 2.2; (33)  6.0 ± 2.5; (33) |
|  | 5 | Challenge with medications (polypharmacy defined as more than 5 drugs, generic drugs vs. original drugs, high-risk drugs, drug-drug interactions). | Likelihood of occurrence  Impact on patient safety | 7.7 ± 2.1; (33)  7.4 ± 2.0; (33) |
|  | 6 | Allergy errors (allergies are not recorded, not or incorrectly documented, or not considered, no information about allergies provided by patients). | Likelihood of occurrence  Impact on patient safety | 5.9 ± 2.5; (32)  7.4 ± 2.1; (33) |
| **Prescribing** | 7 | General prescription errors (e.g., wrong drug, wrong dose, incomplete prescription, other types of errors such as omission errors, transcription errors, duplication errors). | Likelihood of occurrence  Impact on patient safety | 7.1 ± 2.2; (33)  7.3 ± 1.8; (33) |
|  | 8 | Unintentional/intentional override of an electronic warning during prescription, alert fatigue due to repeated, too many or inappropriate warnings. | Likelihood of occurrence  Impact on patient safety | 5.4 ± 2.5; (32)  5.6 ± 2.4; (32) |
|  | 9 | Difficulties with handwritten prescription (e.g., incomplete prescription, illegibility of prescription, prescription written in pencil or “non-waterproof pen“, use of correction varnish). | Likelihood of occurrence  Impact on patient safety | 7.7 ± 2.2; (33)  6.9 ± 2.5; (33) |
|  | 10 | Errors and challenges in electronic prescribing (e.g., lack of usability, errors in automated processes, incorrect use, incorrect/problematic default settings and features; additional need for paper records; technology downtime). | Likelihood of occurrence  Impact on patient safety | 5.2 ± 2.7; (32)  5.2 ± 2.4; (32) |
|  | 11 | Challenges in the prescription of complex/high risk medications (e.g., polypharmacy, lack of control of drug-drug interactions) due to lack of clinical pharmacological knowledge (e.g., irrational, inappropriate, ineffective prescribing) and/or due to lack of prescribing regimens or non-use of existing prescribing regimens. | Likelihood of occurrence  Impact on patient safety | 6.0 ± 2.3; (33)  7.0 ± 2.0; (33) |
|  | 12 | Wrong patient in prescription (e.g., mistaken identity, same name of patient). | Likelihood of occurrence  Impact on patient safety | 4.4 ± 2.5; (32)  7.1 ± 2.5; (33) |
| **Verifying** | 13 | Missing verification/support for complex prescriptions by (clinical) pharmacists (e.g., high-risk medications, polypharmacy, complex indications and diagnoses). | Likelihood of occurrence  Impact on patient safety | 6.1 ± 3.0; (33)  6.3 ± 2.5; (33) |
| **Preparing/Dispensing** | 14 | Errors in preparing/dispensing medications (e.g., errors in splitting tablets, wrong drug, wrong dose, wrong drug calculation, missing or incorrect change of ordered medication in the dispenser, missing or incorrect documentation, missing/incorrect/unclear labeling/marking of prepared medications). | Likelihood of occurrence  Impact on patient safety | 5.9 ± 2.6; (33)  6.7 ± 2.3; (33) |
|  | 15 | Confusion of medications (e.g., look-a-like medication error, sound-a-like medication error, confusion of medication names/packages). | Likelihood of occurrence  Impact on patient safety | 5.4 ± 2.6; (33)  7.0 ± 2.4; (32) |
|  | 16 | No use of guidelines and/or standards for safe preparation/dispensing (e.g., 4-eyes principle or read-back method). | Likelihood of occurrence  Impact on patient safety | 5.4 ± 2.6; (33)  6.2 ± 2.3; (33) |
|  | 17 | Wrong patient when preparing/dispensing medication (e.g., preparing a medication for the wrong patient). | Likelihood of occurrence  Impact on patient safety | 4.6 ± 2.5; (33)  7.2 ± 2.4; (33) |
|  | 18 | Difficulties with supply and storage of medications (e.g., delayed or wrong deliveries, no information regarding originator vs. generic, no safe storage of dispensers/medications). | Likelihood of occurrence  Impact on patient safety | 4.6 ± 2.8; (33)  4.3 ± 2.0; (33) |
|  | 19 | Lack of communication/misunderstandings in communication (e.g., errors in telephone orders, misunderstanding regarding drug name, dosage, interval, type of dosage, patient). | Likelihood of occurrence  Impact on patient safety | 6.1 ± 2.5; (32)  6.7 ± 2.5; (32) |
|  | 20 | Intravenous drug preparation errors (e.g., wrong drug, wrong diluent solution, wrong label, wrong dose, bacterial contamination, incompatibility, or instability). | Likelihood of occurrence  Impact on patient safety | 4.8 ± 2.3; (33)  7.5 ± 2.5; (33) |
| **Administering** | 21 | Errors in administering medications (e.g., wrong drug, wrong dosage, wrong route of administration, confusion of look-a-like or sound-a-like medications, wrong time of administration, unauthorized drugs, omission errors, faulty checking activities, difficulties with infusion equipment, confusion of packaging of medications, incorrect labeling of medication on packaging). | Likelihood of occurrence  Impact on patient safety | 5.2 ± 2.5; (33)  7.5 ± 2.2; (33) |
|  | 22 | Missing documentation and communication about intake of medications, missed intake of medications, drug interactions (e.g., allergies and drug-drug interactions). | Likelihood of occurrence  Impact on patient safety | 5.4 ± 2.5; (33)  6.5 ± 2.1; (33) |
|  | 23 | Wrong patient identification during administration (e.g., misidentification of patient, drug is administered to the wrong patient). | Likelihood of occurrence  Impact on patient safety | 4.1 ± 2.6; (33)  7.3 ± 2.8; (33) |
|  | 24 | Administering medications to patients with cognitive impairment or missing compliance (e.g., missing or incorrect control and monitoring of medication intake, missing or incorrect documentation of administration). | Likelihood of occurrence  Impact on patient safety | 4.8 ± 2.6; (31)  6.3 ± 2.5; (31) |
|  | 25 | Problems with infusion pumps (e.g., incorrect handling, incorrect setting, uncalibrated pumps, different pump characteristics, infusomat lacking lock, lack of flushing, lack of uniform standards). | Likelihood of occurrence  Impact on patient safety | 4.1 ± 2.5; (33)  6.1 ± 2.7; (33) |
|  | 26 | Errors related to barcode technology (e.g. missing/incorrect barcodes, systems not communicating with each other). | Likelihood of occurrence  Impact on patient safety | 3.2 ± 2.1; (32)  4.3 ± 2.7; (32) |
| **Monitoring** | 27 | Errors in further prescription of medications (e.g., without review of reactions/interactions, missing or incorrect prescription or continued prescription, missing or incorrect labeling, missing further prescription before weekends or holidays, further prescription of medication was not stopped). | Likelihood of occurrence  Impact on patient safety | 5.2 ± 2.5; (33)  5.4 ± 2.4; (33) |
|  | 28 | Poor communication among staff (e.g., among nurses or doctors regarding medication such as effect, side effect, interaction, prescription, changes or passing on wrong information). | Likelihood of occurrence  Impact on patient safety | 5.3 ± 2.3; (33)  6.0 ± 2.3; (33) |
| **Discharge** | 29 | Inadequate communication/information between hospital and general practitioners, nursing services, and other health care providers regarding medication needs at discharge; digital communication, interface problems. | Likelihood of occurrence  Impact on patient safety | 6.0 ± 2.7; (32)  6.1 ± 1.8; (32) |
|  | 30 | Missing communication/information with patients and relatives (e.g., medication needs, medications are not explained). | Likelihood of occurrence  Impact on patient safety | 6.3 ± 2.6; (32)  6.4 ± 2.1; (32) |
|  | 31 | Lack of availability of medications in the general practice setting after an inpatient stay (e.g., originator vs. generic drugs). | Likelihood of occurrence  Impact on patient safety | 4.6 ± 2.6; (32)  4.2 ± 2.3; (32) |
|  | 32 | Inadequate/incorrect written discharge information ("discharge letter") (e.g., missing/incomplete prescription of medication, recommended medication is incomplete, discharge letter does not reach patient, general practitioner, specialist, or nursing home or is delayed). | Likelihood of occurrence  Impact on patient safety | 4.9 ± 2.9; (33)  5.6 ± 2.4; (33) |
|  | 33 | Lack of discharge assessment regarding further (nursing) needs of patients (e.g., patients are not able to organize medication by themselves, have problems paying for medication, live far away from general practitioner/pharmacy in a remote area). | Likelihood of occurrence  Impact on patient safety | 5.2 ± 2.8; (33)  5.8 ± 2.3; (33) |
| **Health care professional competence** | 34 | Problematic environment during each step of the medication use process (e.g., noise, poor lighting, emergencies, chaotic work environment, interruption/distraction and heavy staff workload due to e.g., understaffing, poor ward equipment). | Likelihood of occurrence  Impact on patient safety | 6.6 ± 3.0; (33)  6.9 ± 2.6; (33) |
|  | 35 | Knowledge-based errors and lack of training/experience (e.g., lack of knowledge, lack of qualified staff, working with inexperienced or new staff member, frequent staff turnover, and lack of training/education of those involved in the medication use process). | Likelihood of occurrence  Impact on patient safety | 4.9 ± 2.4; (33)  6.5 ± 2.5; (33) |
|  | 36 | Lack of adherence to guidelines/policies regarding the medication use process by staff members and lack of guidelines and standards in the hospital regarding the medication use process. | Likelihood of occurrence  Impact on patient safety | 4.6 ± 2.5; (33)  5.7 ± 2.3; (33) |
|  | 37 | Poor health of staff (e.g., fatigue, physical exhaustion, stress) as well as problematic personalities (e.g., lack of error perception and diligence, complacency). | Likelihood of occurrence  Impact on patient safety | 5.2 ± 2.7; (32)  6.6 ± 2.6; (32) |
|  | 38 | General communication difficulties between healthcare staff along the complete medication use process, deficiencies in communication structure/information flow, lack of "speaking up" culture (voicing e.g., safety deficiencies to other staff), poor local work culture, and lack of supervision/guidance from senior colleagues. | Likelihood of occurrence  Impact on patient safety | 5.5 ± 2.7; (32)  6.8 ± 2.3; (32) |
| **Patient** | 39 | Compliance of family caregivers/relatives regarding medication (e.g., family caregivers do not understand medication information; relatives bring additional medication without consultation; relatives administer medication without consultation). | Likelihood of occurrence  Impact on patient safety | 5.1 ± 2.4; (32)  6.1 ± 2.3; (32) |
|  | 40 | Risk factors related to patients (e.g., lack of compliance, lack of health literacy, lack of knowledge about their own medications, wrong intake of medications). | Likelihood of occurrence  Impact on patient safety | 6.1 ± 2.0; (32)  6.6 ± 2.1; (32) |
| **Digital process and IT-security** | 41 | Missing/deficient hardware (e.g., lack of equipment for the use of electronic systems, outdated devices, poor WLAN technology). | Likelihood of occurrence  Impact on patient safety | 4.5 ± 3.1; (33)  4.7 ± 2.6; (33) |
|  | 42 | Poor software (includes all health information technology in the hospital), missing/poorly designed interfaces between programs used in the medication use process (e.g., at admission, prescription, order, inventory, discharge), poor usability, unanticipated interaction of systems, software bugs, lack of security. | Likelihood of occurrence  Impact on patient safety | 5.3 ± 3.1; (33)  5.2 ± 2.7; (33) |
|  | 43 | Poor implementation of health information technologies (e.g., poor implementation plans, training and strengthening of health care professionals' IT competences has not been carried out). | Likelihood of occurrence  Impact on patient safety | 5.4 ± 3.1; (33)  4.8 ± 2.4; (33) |

Mean ± SD (standard deviation); N = number of experts: Likert scale 1-10 (1 = very low likelihood of occurrence / impact on patient safety; 10 = very high likelihood of occurrence / impact on patient safety)
