# Supplementary material for: Risks in the analogue and digitally-supported medication process and potential solutions to increase patient safety in the hospital: A mixed methods study
Source: PLoS One. 2024 Feb 27;19(2):e0297491. doi: 10.1371/journal.pone.0297491 (PMC10898776; doi:10.1371/journal.pone.0297491)
Supplement: S1 File — Befragung. (PDF) [file pone.0297491.s002.pdf]

Wissenschaftliche Projekte

MeDi-Pro 1. Befragung ()
No. of responses = 33

Legend

Question text

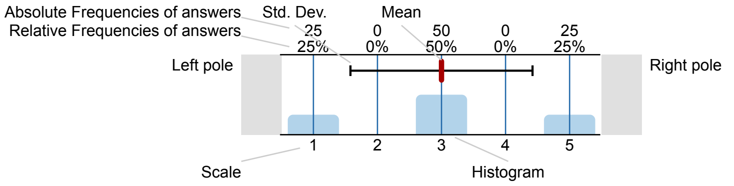

n=No. of responses
av.=Mean
dev.=Std. Dev.
ab.=Abstention

Fragen zur Person

Geschlecht

n=32

Weiblich	56.3%
Männlich	43.8%
Keine Angabe	0%

Alter

n=32

18-30 Jahre	15.6%
31-40 Jahre	18.8%
41-50 Jahre	31.3%
51-60 Jahre	34.4%
≥ 61 Jahre	0%

Beruf (in dem Sie vorwiegend arbeiten)

n=31

Pflegeperson	32.3%
Arzt/Ärztin	29%
Qualitätsmanagement/ Risikomanagement	22.6%
Management	6.5%
Sonstiges:	9.7%

Sonstiges:

- Apotheker
- Pflegewissenschsftlerin
- Wissenschaftler

Berufserfahrung

n=32

0-2 Jahre	3.1%
3-6 Jahre	12.5%
7-10 Jahre	9.4%
11-20 Jahre	25%
>20 Jahre	50%

Code zur Wiedererkennung in der 2.Delphi-Befragung Geburtsjahr Mutter z.B. 1955

- 1922
- 1929 (2 Counts)
- 1932
- 1937
- 1938 (2 Counts)
- 1941 (2 Counts)
- 1942
- 1944 (2 Counts)
- 1947
- 1948
- 1950
- 1951
- 1952
- 1953
- 1954 (2 Counts)
- 1955 (2 Counts)
- 1956
- 1959
- 1963 (2 Counts)
- 1965
- 1966
- 1967
- 1968 (2 Counts)
- 1969
- 1985

1. Buchstabe Vorname Mutter

- A (3 Counts)
- B
- C (2 Counts)
- D
- E (2 Counts)
- G
- H (4 Counts)
- I (3 Counts)
- M
- R (5 Counts)
- S (2 Counts)
- T
- U

- W (2 Counts)
- b
- c (2 Counts)
- i

1. Buchstabe Vorname Vater

- A (4 Counts)
- E
- F (5 Counts)
- G (2 Counts)
- H (2 Counts)
- J (4 Counts)
- K
- M (2 Counts)
- R (5 Counts)
- S
- W (2 Counts)
- f (2 Counts)
- g
- r

Allgemeine Information

Wir ersuchen Sie, die Risikocluster bestmöglich nach Eintrittswahrscheinlichkeit und Auswirkung zu bewerten. Die Risikocluster bestehen aus einzelnen Risiken, die beispielhaft für eine Gruppe von einzelnen Risiken stehen.
Zur Bewertung der Risikocluster steht eine Skala von 1-10 zur Verfügung, wobei 10 die höchste Eintrittswahrscheinlichkeit und fatale Auswirkung und 1 keine bis sehr geringe Eintrittswahrscheinlichkeit sowie geringe Auswirkung für die Patient*innen darstellt. Bei der Bewertung berücksichtigen Sie immer das aus **Ihrer Sicht größte Risiko** für den jeweiligen Risikocluster, unabhängig davon, wie Sie die anderen einzelnen Risiken in diesem Cluster bewerten.

Bei jeder Frage, haben Sie auch die Möglichkeit, einen Kommentar zu dem Risikocluster abzugeben. Das wäre beispielsweise das Fehlen von weiteren Risiken, in diesem Risikocluster.

Ziel der Befragung ist es, die relevantesten Risiken im Medikationsprozess zu identifizieren.

Kapitel: Aufnahme

Risikocluster: **Unzureichende Kommunikation** über die verordneten Medikamente zwischen dem niedergelassenen Bereich und dem Krankenhaus.

Wie schätzen Sie die **Eintrittswahrscheinlichkeit** von diesem Risikocluster im Krankenhaus ein? Kreuzen Sie bitte an:

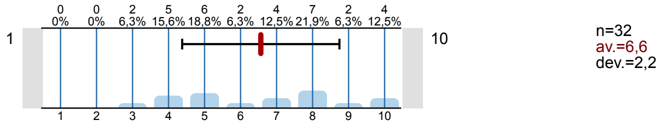

Wie schätzen Sie die **Auswirkungen** dieses Risikoclusters für Patient*innen im Krankenhaus ein? Kreuzen Sie bitte an:

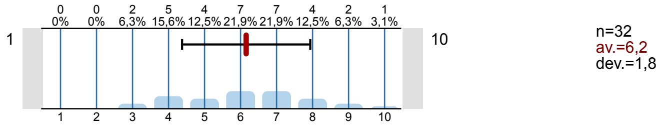

Haben Sie Anmerkungen?

- .
- ELGA bringt Verbesserung, viele Patienten bzw. Angehörige können Auskunft über tatsächliche Medikation geben
- Gesundheitssysteme in den Ländern sind verschieden - auch abhängig von den jeweiligen Dokumentationssystemen die Bereichsübergreifend vorhanden sind. -gehe ich in der Beurteilung von meinem Krankenhaus aus? von was für einem Patienten gehe ich aus?. einem informierten Patienten? oder einem Patienten der bei seinem Medikamentenmanagement Hilfe braucht?
- Meist hat der Patient eine Media-Liste oder die Medikamente Schachteln mit. Leider sind in ELGA keine Dosierungen.
- Teilweise vergessen Pat. im Rahmen des Aufnahmeprozesses ins KH Medika anzugeben wie z.B. Bedarfsmed., Schlafmed. oder NMH bei pausierter oAK, wenn diese nicht schriftlich dokumentiert wurden.

- Unzureichende Reglementierung der eMedikation (verpflichtende Inhalte?) führt dazu, dass Informationen nicht nutzbar sind.
- eMedikationsprozess über eCard derzeit unzureichend. Abgesetzte Medikamente oft nicht erkennbar.
- kann nicht beurteilt werden

Risikocluster: **Unvollständige Medikationsliste bei Aufnahme** mit Diskrepanzen in der Medikamentenhistorie (z.B. unterschiedliche Listen von Patient*in, Hausarzt*in, Facharzt*in, elektronischer Medikamentenerfassung).

Wie schätzen Sie die **Eintrittswahrscheinlichkeit** von diesem Risikocluster im Krankenhaus ein? Kreuzen Sie bitte an:

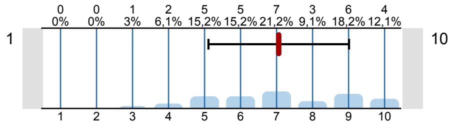

n=33
av.=7,1
dev.=2

Wie schätzen Sie die **Auswirkungen** dieses Risikoclusters für Patient*innen im Krankenhaus ein? Kreuzen Sie bitte an:

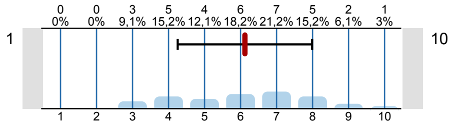

n=33
av.=6,1
dev.=1,9

Haben Sie Anmerkungen?

- .
- Fehlende Dokumentation, wenn Pat. bspw. eigenmächtig Dosierungen von Medikamenten ändern oder selbstständig absetzen.
- Hoch bei nicht-ansprechbaren, nicht-geschäftsfähigen Patient*innen. Mittel bei anderen, da im ärztlichen Gespräch dies eigentlich bereinigt werden sollte.
- Ist sehr abhängig von Hausarzt, der Familie des Patienten usw. - Zudem spielt die Vorbereitung ins Spital eine wesentliche Rolle - welche Dokumente usw. erhält der Patient die Patientin für den Eintritt
- Siehe oben
- keine einheitliche digitale Krankenakte die von allen spezifischen Berufsgruppen gepflegt wird

---

Risikocluster: **Patient*innen-bezogene Prädiktoren bei Aufnahme** (z.B. mangelnde Compliance, Gesundheitsstatus, Art der Aufnahme (akut, elektiv) oder Gesundheitskompetenz).

Wie schätzen Sie die **Eintrittswahrscheinlichkeit** von diesem Risikocluster im Krankenhaus ein? Kreuzen Sie bitte an:

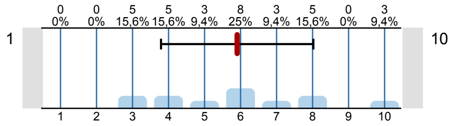

n=32
av.=5,9
dev.=2,1

Wie schätzen Sie die **Auswirkungen** dieses Risikoclusters für Patient*innen im Krankenhaus ein? Kreuzen Sie bitte an:

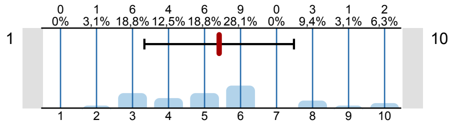

n=32
av.=5,4
dev.=2,1

Haben Sie Anmerkungen?

- .
- Risiko in Kombination mit den beiden vorangegangenen Clustern relevant, sonst sollte die klinische Professionalität und der Obsorgeauftrag dieses Risiko minimieren.
- kann nicht beurteilt werden
- sehr Abteilungsspezifisch- Bereiche mit hoher Anzahl betagter und hochbetagter Patienten haben ein höheres Risiko

---

Risikocluster: **Geringe berufliche Erfahrung** des aufnehmenden Arztes/ der aufnehmenden Ärztin bzw. fehlender Support durch erfahrene Ärzt*innen im Krankenhaus.

Wie schätzen Sie die **Eintrittswahrscheinlichkeit** von diesem Risikocluster im Krankenhaus ein? Kreuzen Sie bitte an:

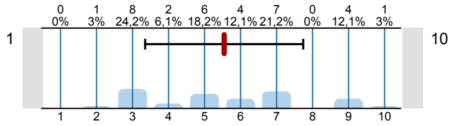

n=33
av.=5,5
dev.=2,2

Wie schätzen Sie die **Auswirkungen** dieses Risikoclusters für Patient*innen im Krankenhaus ein? Kreuzen Sie bitte an:

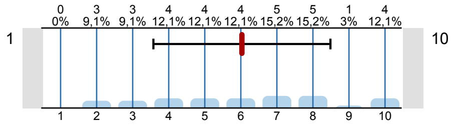

n=33
av.=6
dev.=2,5

Haben Sie Anmerkungen?

- .
- Aus meiner Sicht besteht das Hauptproblem darin, dass bei Eintritt (whs. Abhängig vom Bereich, z. B. Intensivmedizin) alle eigenen Medikamente gestoppt werden. Was oftmals auch Sinn macht - das Problem ist jedoch später auf der Abteilung, dass das Interesse der Behandelnden Ärzte die Medikamentenliste für den Patienten zu straffen und mit ihm anzuschauen, wie die Compliance verbessert werden könnte (z. B. alle Medis am Morgen, dann ist es für den Tag erledigt, nicht erfolgt.
- Dies ist neben der beruflichen Erfahrung auch von der Persönlichkeitsstruktur, des Lernverhaltens der Fachperson abhängig? Wissen wann und wie Support geholt werden kann.
- Hängt stark von der Selbstreflexionsfähigkeit der Aufnehmenden ab, bzw. von der Hierarchie und Erreichbarkeit erfahrener Ärzt*innen.
- Wenn Medikationsverordnung NICHT von erfahrenem Arzt und/oder Pflege kontrolliert und hinterfragt wird- hohes Risiko

Risikocluster: **Herausforderung bei Medikamenten** (Polypharmazie (definiert als mehr als 5 Medikamente), Generika vs. Originator, Hochrisikomedikamente, Wechselwirkungen von Medikamenten).

Wie schätzen Sie die **Eintrittswahrscheinlichkeit** von diesem Risikocluster im Krankenhaus ein? Kreuzen Sie bitte an:

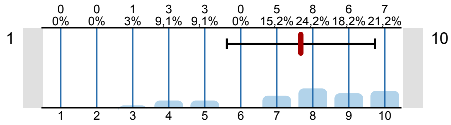

n=33
av.=7,7
dev.=2,1

Wie schätzen Sie die **Auswirkungen** dieses Risikoclusters für Patient*innen im Krankenhaus ein? Kreuzen Sie bitte an:

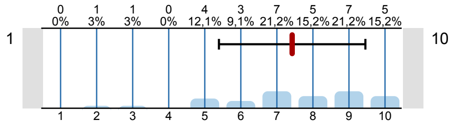

n=33
av.=7,4
dev.=2

Haben Sie Anmerkungen?

- .
- Aufmerksamkeit diesem Thema gegenüber ist gestiegen, Patienten in meinem Bereich nehmen generell viele Medikamente ein
- Polypragmasie (als Zeichen von Hilflosigkeit) ist eines DER Probleme in Risikopopulationen (Geriatrie, Psychiatrie, etc.).
- Wobei hier mMn das Risiko nicht aufgrund der Polypharmazie bzw. der Medikamente an sich besteht, sondern der Weigerung/Nicht-Benutzung von Medikamenten-Katalogen und Interaktionsprüfungen/Austauschpräparatelisten durch die Anwender*innen.

Risikocluster: **Allergiefehler** - Allergien werden nicht erhoben, nicht oder falsch dokumentiert oder nicht berücksichtigt, keine Angabe einer Allergie durch Patient*innen.

Wie schätzen Sie die **Eintrittswahrscheinlichkeit** von diesem Risikocluster im Krankenhaus ein? Kreuzen Sie bitte an:

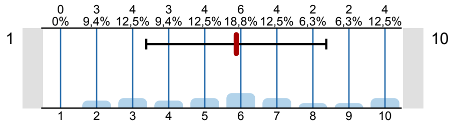

n=32
av.=5,9
dev.=2,5

Wie schätzen Sie die **Auswirkungen** dieses Risikoclusters für Patient*innen im Krankenhaus ein? Kreuzen Sie bitte an:

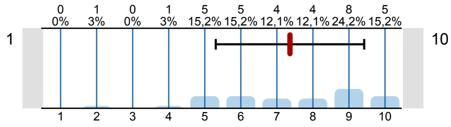

n=33
av.=7,4
dev.=2,1

Haben Sie Anmerkungen?

- .
- Es besteht auch das umgekehrte Problem: Eine ungenaue Definition, was eine Allergie ist. Durch vermutete Allergien, die mit den Diagnosen mitgeschleppt werden, bekommen Patienten ev. Therapien nicht (Penicillin, Aspirin, etc.)
- Ist abhängig ob die Allergien bekannt sind, Allergiepass wird bei uns beim elektiven Eintritt ins Spital verlangt.
- Wird sehr genau erhoben durch Ärzte und Pflege

Kapitel: Verordnung

Risikocluster: **Allgemeine Fehler in der Verordnung** (z.B. falsches Medikament, falsche Dosis, unvollständige Verordnung und andere Fehlertypen wie Auslassungsfehler, Übertragungsfehler, Duplizierungsfehler).

Wie schätzen Sie die **Eintrittswahrscheinlichkeit** von diesem Risikocluster im Krankenhaus ein? Kreuzen Sie bitte an:

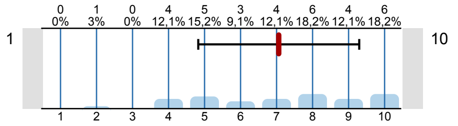

n=33
av.=7,1
dev.=2,2

Wie schätzen Sie die **Auswirkungen** dieses Risikoclusters für Patient*innen im Krankenhaus ein? Kreuzen Sie bitte an:

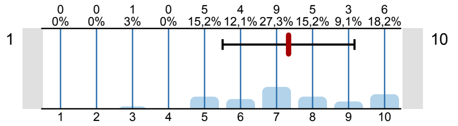

n=33
av.=7,3
dev.=1,8

Haben Sie Anmerkungen?

- .
- Abhängig von der Dokumentation, ob dies elektronisch oder auf Papiergeschieht. Bei uns wird elektronisch dokumentiert, mit eingegebenen Grenzen und in der Regel werden Medikamentenverordnungen mit Oberärzten /Oberärztinnen abgesprochen.
- Bei elektronischer Dokumentation nicht selten, weil der Patient nicht am Patientenbett, sondern aus dem Arzt-Büro visitiert wird.
- Detliche Verbesserung durch elektronische Fieberkurve!
- Pflegepersonen weisen auf unvollständige Verordnungen hin
- kommt nahezu täglich vor, Pflege kontrolliert sehr genau und weist auf Verordnungsfehler hin - hoher Zeitaufwand
- solltet ihr hier einen Hinweis bekommen - eine Anmerkungen zu dieser Frage haben sich durch das Risikocluster mit den Fragen 4.7 und 4.8 erübrigt.

Risikocluster: **Unbeabsichtigtes/Beabsichtigtes Übergehen einer elektronischen Warnung bei Verordnung**, Alarmermüdung durch wiederholte, zu viele oder unangemessene Warnungen.

Wie schätzen Sie die **Eintrittswahrscheinlichkeit** von diesem Risikocluster im Krankenhaus ein? Kreuzen Sie bitte an:

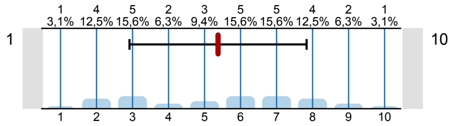

n=32
av.=5,4
dev.=2,5

Wie schätzen Sie die **Auswirkungen** dieses Risikoclusters für Patient*innen im Krankenhaus ein? Kreuzen Sie bitte an:

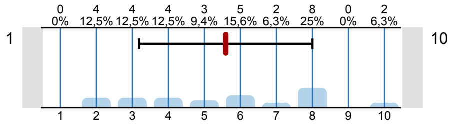

n=32
av.=5,6
dev.=2,4

Haben Sie Anmerkungen?

- Abhängig wie die Sensibilität eingestellt ist- wenn es immer alarmt - wird der Alarm irgendwann nicht mehr gesehen.
- Die Warnungen bei Verordnungen sind nur bei einzelnen besonders risikobehafteten Präparaten sinnvoll.
  Sobald mehrere Medikamente verschrieben werden müssen oder schwere Grunderkrankungen, wie Niereninsuff. oder Leberinsuffizienz vorliegen, ist der Wert solcher Warnsysteme derzeit gering
- Oftmals ist bei Fehlermeldungen mittels Pop-Ups die Assoziation mit einem technischen Gebrechen des Programmes vorhanden. (z. B. Eingabe unvollständig, mit Server nicht verbunden, etc.) Daher glaube ich, dass es ein Risiko darstellt, Warnungen zu Verordnungen und zu technischen Hinweisen gleich zu designen. Idealerweise werden verschiedene Systeme verwendet (z.B. Pop-Ups für Technische Fehlermeldungen und ein Warnscreen mit Checkbox für Verordnungswarnungen)
- derzeit noch keine eFK, Annahme
- kann nicht beurteilt werden

Risikocluster: **Schwierigkeiten mit der handschriftlichen Verordnung** (z.B. unvollständige Verordnung, Unleserlichkeit der Verordnung, Verordnung mit Bleistift oder „nicht wasserfestem Stift“, Verwendung von Korrekturlack).

Wie schätzen Sie die **Eintrittswahrscheinlichkeit** von diesem Risikocluster im Krankenhaus ein? Kreuzen Sie bitte an:

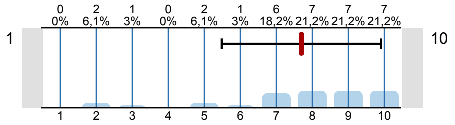

n=33
av.=7,7
dev.=2,2

Wie schätzen Sie die **Auswirkungen** dieses Risikoclusters für Patient*innen im Krankenhaus ein? Kreuzen Sie bitte an:

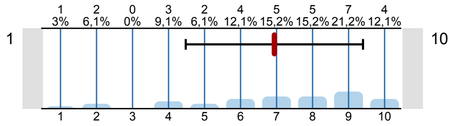

n=33
av.=6,9
dev.=2,5

Haben Sie Anmerkungen?

- .
- Durch eFK verbesserbar
- In unserem Krankenhaus erfolgen keine handschriftlichen Verordnungen, nur elektronische Verordnungen werden ausgeführt
- Manchmal lassen sich Unklarheiten bei handschriftlichen Verordnungen nur durch anordnende Person beseitigen, v.a. wenn die ausführende Person mit der Handschrift nicht vertraut ist.
- Pflege fragt nach, wenn etwas nicht gelesen werden kann. Fehler passieren so selten.
- kommt nahezu täglich vor, Pflege kontrolliert sehr genau und weist auf Verordnungsfehler, nicht Lesbarkeit usw. hin - hoher Zeitaufwand

Risikocluster: **Fehler und Herausforderungen in der elektronischen Verordnung** (z.B. mangelnde Benutzerfreundlichkeit, Fehler in automatisierten Prozessen, falscher Gebrauch, fehlerhafte/problematische Standardeinstellungen und Features; zusätzlicher Bedarf an Papierdokumenten; Technikausfall).

Wie schätzen Sie die **Eintrittswahrscheinlichkeit** von diesem Risikocluster im Krankenhaus ein? Kreuzen Sie bitte an:

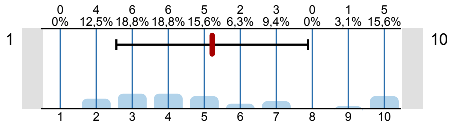

n=32
av.=5,2
dev.=2,7

Wie schätzen Sie die **Auswirkungen** dieses Risikoclusters für Patient*innen im Krankenhaus ein? Kreuzen Sie bitte an:

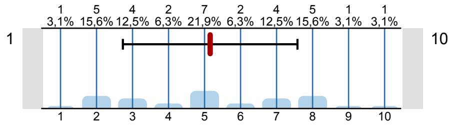

n=32
av.=5,2
dev.=2,4

Haben Sie Anmerkungen?

- .
- Noch keine eFK, Annahme
- Wenn das Anwendungssystem bereits gut eingeführt ist, ansonsten tendenziell höheres Risiko (8/8) in der Implementierungs- und Refreezingphase.
- kann nicht beurteilt werden

Risikocluster: **Herausforderungen in der Verordnung von komplexen Medikamenten/Hochrisikomedikamenten** (z.B. Polypharmazie, mangelnde Kontrolle der Arzneimittelwechselwirkungen) durch Mangel an klinisch-pharmakologischem Wissen (z.B. irrationale, unangemessene und ineffektive Verordnung) und/oder durch fehlende Verordnungsschemata oder Nicht-Verwendung vorhandener Verordnungsschemata.

Wie schätzen Sie die **Eintrittswahrscheinlichkeit** von diesem Risikocluster im Krankenhaus ein? Kreuzen Sie bitte an:

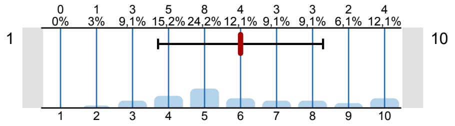

n=33
av.=6
dev.=2,3

Wie schätzen Sie die **Auswirkungen** dieses Risikoclusters für Patient*innen im Krankenhaus ein? Kreuzen Sie bitte an:

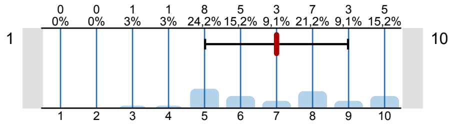

n=33
av.=7
dev.=2

Haben Sie Anmerkungen?

- .
- Ein hochvariabler Risikocluster (Ausbildungsstand, Arbeitsteilung, Abteilungskultur, etc.)

- In unserem Krankenhaus enge Zusammenarbeit mit der Pharmazie
- Schätze ich grundsätzlich hoch ein - wir arbeiten mit einem klinischen Pharmakologen, genau um diese Probleme zu vermeiden
- kommt sicher häufig vor
  Pharmazeut bei Visite mehrmals pro Woche wäre hilfreich

Risikocluster: **Falsche/r Patient*in bei der Verordnung** (z.B. Verwechslung der Identität, gleicher Name der Patient*innen).

Wie schätzen Sie die **Eintrittswahrscheinlichkeit** von diesem Risikocluster im Krankenhaus ein? Kreuzen Sie bitte an:

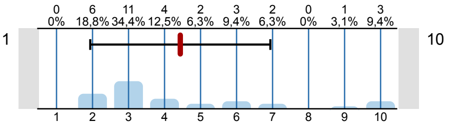

n=32
av.=4,4
dev.=2,5

Wie schätzen Sie die **Auswirkungen** dieses Risikoclusters für Patient*innen im Krankenhaus ein? Kreuzen Sie bitte an:

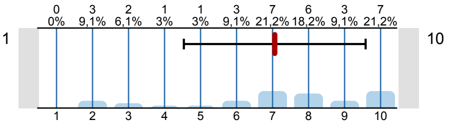

n=33
av.=7,1
dev.=2,5

Haben Sie Anmerkungen?

- .
- Geordnete Workflows haben Risiko reduziert
- In gewissen Abteilungen werden Medikamente usw. gescannt - künftig soll dies flächendeckend sein
- Namensgleichheit kommt nicht oft vor, wird immer darauf hingewiesen
  Kann vorkommen- eher sehr selten

Kapitel: Überprüfung

Risikocluster: **Fehlende Überprüfung/Unterstützung bei komplexen Verordnungen von (klinischen) Pharmazeut*innen** (z.B. Hochrisikomedikamente, Polypharmazie, komplexe Indikationen und Diagnosen).

Wie schätzen Sie die **Eintrittswahrscheinlichkeit** von diesem Risikocluster im Krankenhaus ein? Kreuzen Sie bitte an:

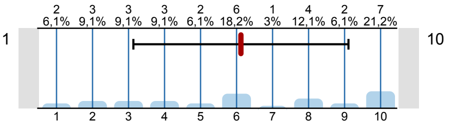

n=33
av.=6,1
dev.=3

Wie schätzen Sie die **Auswirkungen** dieses Risikoclusters für Patient*innen im Krankenhaus ein? Kreuzen Sie bitte an:

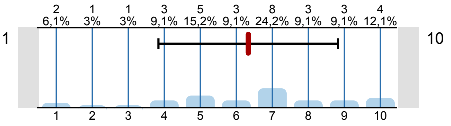

n=33
av.=6,3
dev.=2,5

Haben Sie Anmerkungen?

- .
- Die Eintrittswahrscheinlichkeit hängt hier natürlich extrem von den lokal und national vorhandenen Strukturen und Ressourcen ab.
- Enge Zusammenarbeiten, regelmässige Visiten mit den Pharmazeuten in unserem Krankenhaus
- International längst Standard!

Kapitel: Vorbereitung/Dispensierung

Risikocluster: **Fehler bei der Vorbereitung/Dispensierung von Medikamenten** (z.B. Fehler bei der Teilung der Tabletten, falsches Medikament, falsche Dosis, falsche Berechnung, fehlende oder falsche Änderung des angeordneten Medikaments im Dispenser, fehlende oder falsche Dokumentation, fehlende/falsche/unklare Beschriftung/Kennzeichnung vorbereiteter Medikamente).

Wie schätzen Sie die **Eintrittswahrscheinlichkeit** von diesem Risikocluster im Krankenhaus ein? Kreuzen Sie bitte an:

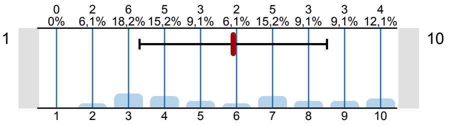

n=33
av.=5,9
dev.=2,6

Wie schätzen Sie die **Auswirkungen** dieses Risikoclusters für Patient*innen im Krankenhaus ein? Kreuzen Sie bitte an:

1 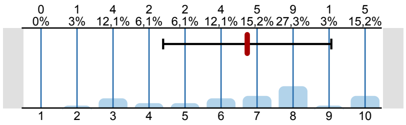

10

n=33
av.=6,7
dev.=2,3

Haben Sie Anmerkungen?

- .
- 4- Augenprinzip, wenn möglich (eher selten machbar in den meisten Bereichen)- Kontrolle der dispensierten Medikamente
- Rascher Wechsel von Ersatzpräparaten kann das Risiko massiv steigern
- Wir haben auf unserer Abteilung häufig ähnliche Dosierungen - und eine begrenzte Anzahl an Medikamenten - gezielt definierter Medikamentensatz

Risikocluster: **Verwechslung von Medikamenten** (z.B. Fehler mit ähnlich aussehenden Medikamenten, Fehler mit ähnlich klingenden Medikamentennamen, Verwechslung von Medikamentennamen / -verpackungen).

Wie schätzen Sie die **Eintrittswahrscheinlichkeit** von diesem Risikocluster im Krankenhaus ein? Kreuzen Sie bitte an:

1 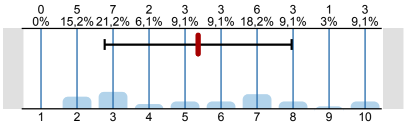

10

n=33
av.=5,4
dev.=2,6

Wie schätzen Sie die **Auswirkungen** dieses Risikoclusters für Patient*innen im Krankenhaus ein? Kreuzen Sie bitte an:

1 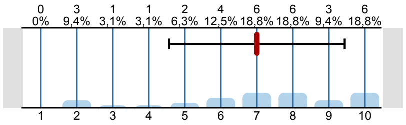

10

n=32
av.=7
dev.=2,4

Haben Sie Anmerkungen?

- .
- 4- Augenprinzip, wenn möglich (eher selten machbar in den meisten Bereichen- Personalaufwand)- Kontrolle der dispensierten Medikamente später durch Kollegen
- 4-Augenprinzip ist etabliert
- Die immer häufigere Verwendung von Generika zwingt einen die Medikamente sehr gut zu kontrollieren
- Prävention durch gezielte Etikettierung - und einen definierten Medikamentesatz

Risikocluster: **Keine Anwendung von Richtlinien und/oder Standards zur sicheren Vorbereitung/Dispensierung** (z.B. 4-Augen-Prinzip oder Readback-Methode).

Wie schätzen Sie die **Eintrittswahrscheinlichkeit** von diesem Risikocluster im Krankenhaus ein? Kreuzen Sie bitte an:

1 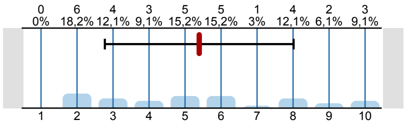

10

n=33
av.=5,4
dev.=2,6

Wie schätzen Sie die **Auswirkungen** dieses Risikoclusters für Patient*innen im Krankenhaus ein? Kreuzen Sie bitte an:

1 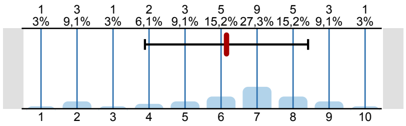

10

n=33
av.=6,2
dev.=2,3

Haben Sie Anmerkungen?

- .
- 4- Augenprinzip, wenn möglich (eher selten machbar in den meisten Bereichen)- Kontrolle der dispensierten Medikamente später durch Kollegen
- Da ich die genannten Verfahren für relativ ineffektiv halte, schätze ich auch die Auswirkungen auf das Fehlen der Verfahren relativ gering ein. Das Auslassen einer unwirksamen Präventionsmassnahme richtet vermutlich relativ wenig Schaden an...
- Wir haben verschiedene Standards aber auch Kompatibilitätsliste usw.

Risikocluster: **Falsche/r Patient*in bei Vorbereitung/Dispensierung der Medikamente** (z.B. Vorbereitung eines Medikaments für den/der falsche/n Patient*in).

Wie schätzen Sie die **Eintrittswahrscheinlichkeit** von diesem Risikocluster im Krankenhaus ein? Kreuzen Sie bitte an:

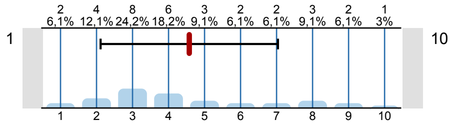

n=33
av.=4,6
dev.=2,5

Wie schätzen Sie die **Auswirkungen** dieses Risikoclusters für Patient*innen im Krankenhaus ein? Kreuzen Sie bitte an:

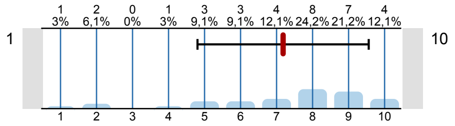

n=33
av.=7,2
dev.=2,4

Haben Sie Anmerkungen?

- .
- 4-Augenprinzip ist etabliert
- Pflegefachpersonen haben einen bis zwei Patienten, bei denen sie die Medikamente vorbereiten und verabreichen.

Risikocluster: **Schwierigkeiten bei Lieferung und Lagerung von Medikamenten** (z.B. verspätete oder falsche Zustellungen, keine Informationen hinsichtlich Originator vs. Generika, keine sichere Lagerung der Dispenser/Medikamente).

Wie schätzen Sie die **Eintrittswahrscheinlichkeit** von diesem Risikocluster im Krankenhaus ein? Kreuzen Sie bitte an:

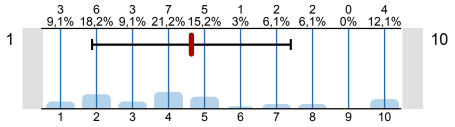

n=33
av.=4,6
dev.=2,8

Wie schätzen Sie die **Auswirkungen** dieses Risikoclusters für Patient*innen im Krankenhaus ein? Kreuzen Sie bitte an:

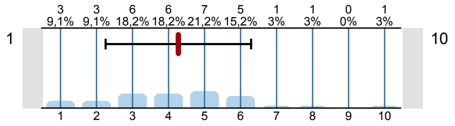

n=33
av.=4,3
dev.=2

Haben Sie Anmerkungen?

- .
- Rascher Wechsel von Ersatzpräparaten kann das Risiko massiv steigern
- viele Generika, viele Durchlaufartikel, keine tägliche Bestellung von Apo ad Firmen usw...

Risikocluster: **Mangelnde Kommunikation/Missverständnisse in der Kommunikation** (z.B. Fehler bei telefonischen Anordnungen, Missverständnisse in Bezug auf Medikamentennamen, Dosierung, Intervall, Dosierungsform, Patient*in).

Wie schätzen Sie die **Eintrittswahrscheinlichkeit** von diesem Risikocluster im Krankenhaus ein? Kreuzen Sie bitte an:

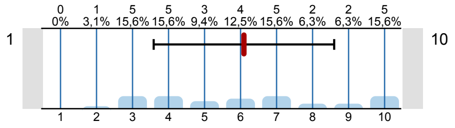

n=32
av.=6,1
dev.=2,5

Wie schätzen Sie die **Auswirkungen** dieses Risikoclusters für Patient*innen im Krankenhaus ein? Kreuzen Sie bitte an:

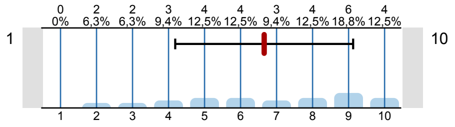

n=32
av.=6,7
dev.=2,5

Haben Sie Anmerkungen?

- Abhängig von Erfahrung der Pflege, ob nachgefragt wird, weil z. B. Dosierung zu hoch ist oder fehlt...
- Beispiele aus der klinischen Praxis: Suggammadex wurde mit Succinylcholin verwechselt, da "Suggi" gesagt wurde. Esmeron wurde mit Esmolol verwechselt, da es offenbar falsch vom Gegenüber verstanden worden ist.
- Durch eFK behoben

Risikocluster: **Fehler bei der Vorbereitung intravenöser Medikamente** (z.B. falsches Medikament, falsche Verdünnungsmittellösung, falsche Kennzeichnung, falsche Dosis, bakterielle Kontamination, Inkompatibilität oder Instabilität).

Wie schätzen Sie die **Eintrittswahrscheinlichkeit** von diesem Risikocluster im Krankenhaus ein? Kreuzen Sie bitte an:

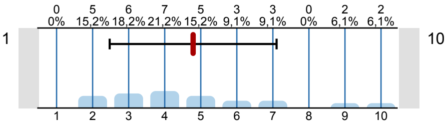

n=33
av.=4,8
dev.=2,3

Wie schätzen Sie die **Auswirkungen** dieses Risikoclusters für Patient*innen im Krankenhaus ein? Kreuzen Sie bitte an:

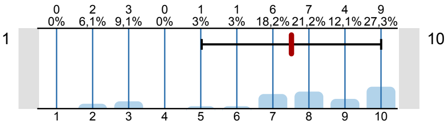

n=33
av.=7,5
dev.=2,5

Haben Sie Anmerkungen?

- .
- Unsere Abteilung besitzt Kompatibilitätslisten im Dokumentationssystem, Verdünnungsvorgaben usw.
- in meinem Bereich viel iv- Medikation, klare Vorgehensweisen, Überprüfung der vorbereiteten Medika durch andere Pflegeperson

Kapitel: Verabreichung

Risikocluster: **Fehler bei der Verabreichung von Medikamenten** (z.B. falsches Medikament, falsche Dosierung, falscher Applikationsweg, Verwechslung von ähnlich aussehenden oder ähnlich klingenden Medikamenten, falsche Zeit der Verabreichung, nicht autorisierte Arzneimittel, Auslassungsfehler, fehlerhafte Überprüfungsaktivitäten, Schwierigkeiten mit Infusionsgeräten, Verwechslung der Verpackung der Medikamente, falsche Kennzeichnung des Medikaments auf der Verpackung).

Wie schätzen Sie die **Eintrittswahrscheinlichkeit** von diesem Risikocluster im Krankenhaus ein? Kreuzen Sie bitte an:

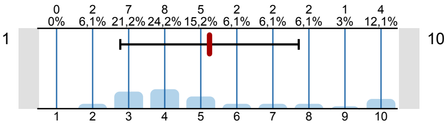

n=33
av.=5,2
dev.=2,5

Wie schätzen Sie die **Auswirkungen** dieses Risikoclusters für Patient*innen im Krankenhaus ein? Kreuzen Sie bitte an:

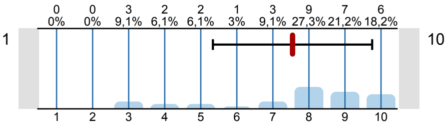

n=33
av.=7,5
dev.=2,2

Haben Sie Anmerkungen?

- .
- Wir verabreichen bei uns aufgrund der Station die meisten Medikamente i/V- ist sicherlich abhängig von welcher Abteilung gesprochen wird.

Risikocluster: **Fehlende Dokumentation und Kommunikation über Einnahme von Medikamenten, fehlende Einnahme, Wechselwirkung** (z.B. Allergien und Arzneimittel-Wechselwirkungen).

Wie schätzen Sie die **Eintrittswahrscheinlichkeit** von diesem Risikocluster im Krankenhaus ein? Kreuzen Sie bitte an:

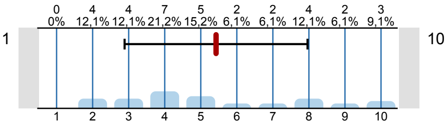

n=33
av.=5,4
dev.=2,5

Wie schätzen Sie die **Auswirkungen** dieses Risikoclusters für Patient*innen im Krankenhaus ein? Kreuzen Sie bitte an:

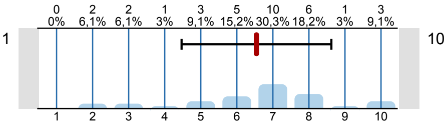

n=33
av.=6,5
dev.=2,1

Haben Sie Anmerkungen?

- .
- Passiert whs. ehre bei handschriftlicher Dokumentation
- Zuwenig diplomiertes Personal erhöht das Risiko
- wird durch Pflege sehr genau dokumentiert

Risikocluster: **Falsche Patient*innen-Identifikation bei der Verabreichung** (z.B. falsche Identifizierung der Patient*innen, Medikament wird dem/der falschen Patient*in verabreicht).

Wie schätzen Sie die **Eintrittswahrscheinlichkeit** von diesem Risikocluster im Krankenhaus ein? Kreuzen Sie bitte an:

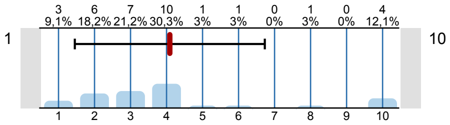

n=33
av.=4,1
dev.=2,6

Wie schätzen Sie die **Auswirkungen** dieses Risikoclusters für Patient*innen im Krankenhaus ein? Kreuzen Sie bitte an:

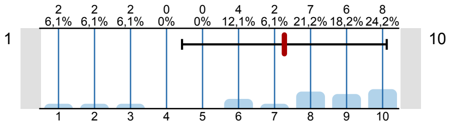

n=33
av.=7,3
dev.=2,8

Haben Sie Anmerkungen?

- Auswirkung hängt von Art des Medikaments ab
- Das standardmäßige Schauen auf die Identifikationsarmbänder wird von PatientInnen als äußerst unprofessionell empfunden.
- Pflegefachpersonen betreuen bei uns 1-2 Patienten - sicherlich abhängig von der Anzahl zu betreuenden Patienten und der Qualifikation der Fachperson.
- Pflegegruppen reduzieren das Risiko

Risikocluster: **Verabreichung von Medikamenten an Patient*innen mit kognitiver Beeinträchtigung oder fehlender Compliance** (z. B. fehlende oder falsche Kontrolle und Überwachung der Medikamenteneinnahme, fehlende oder falsche Dokumentation der Verabreichung).

Wie schätzen Sie die **Eintrittswahrscheinlichkeit** von diesem Risikocluster im Krankenhaus ein? Kreuzen Sie bitte an:

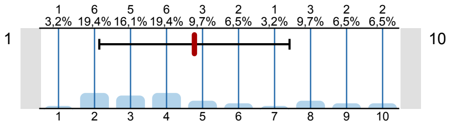

n=31
av.=4,8
dev.=2,6

Wie schätzen Sie die **Auswirkungen** dieses Risikoclusters für Patient*innen im Krankenhaus ein? Kreuzen Sie bitte an:

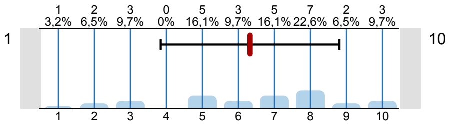

n=31
av.=6,3
dev.=2,5

Haben Sie Anmerkungen?

- .
- Diese Patienten haben eine kontrollierte Medikaeingabe
- keine Angabe möglich
- kontrollierte Medikamenteneinnahme, sobald einmal bemerkt wird, dass Pat. Medika nicht nimmt

Risikocluster: **Probleme mit Infusionspumpen** (z.B. falsches Handling, falsche Einstellung, unkalibrierte/ungeeichte Pumpen, unterschiedliche Pumpeneigenschaften, Infusomaten ohne Verriegelung, fehlende Spülung, fehlende einheitliche Standards).

Wie schätzen Sie die **Eintrittswahrscheinlichkeit** von diesem Risikocluster im Krankenhaus ein? Kreuzen Sie bitte an:

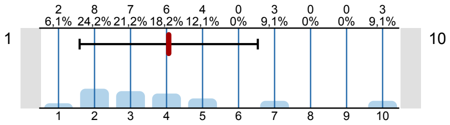

n=33
av.=4,1
dev.=2,5

Wie schätzen Sie die **Auswirkungen** dieses Risikoclusters für Patient*innen im Krankenhaus ein? Kreuzen Sie bitte an:

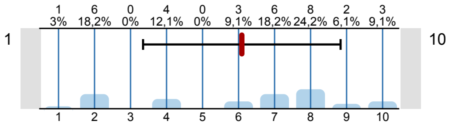

n=33
av.=6,1
dev.=2,7

Haben Sie Anmerkungen?

- .
- Arbeit mit den technischen Hilfsmitteln ist bei uns Routine
- Grundsätzlich sollten die Pumpten so konfiguriert sein, dass kaum Fehler möglich sind - unsere Erfahrungen sind positi
- Zuwenig diplomiertes Personal erhöht das Risiko
- alle MA sind eingeschult, immer ein 2. MA im Dienst, der Pumpen bedienen kann
  einheitliche Pumpen (ein Fabrikat)

Risikocluster: **Fehler im Zusammenhang mit der Barcode-Technologie** (z.B. fehlende/fehlerhafte Barcodes, nicht miteinander kommunizierende Systeme).

Wie schätzen Sie die **Eintrittswahrscheinlichkeit** von diesem Risikocluster im Krankenhaus ein? Kreuzen Sie bitte an:

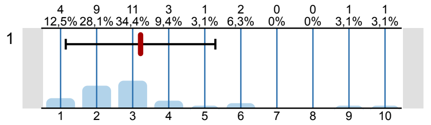

n=32
av.=3,2
dev.=2,1

Wie schätzen Sie die **Auswirkungen** dieses Risikoclusters für Patient*innen im Krankenhaus ein? Kreuzen Sie bitte an:

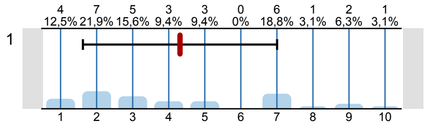

n=32
av.=4,3
dev.=2,7

Haben Sie Anmerkungen?

- .
- Systeme werden im Vorfeld normalerweise abgeglichen
- Wir arbeiten nicht damit - ich hätte gerne "nicht zutreffend" angekreuzt
- für mich nicht beurteilbar

Kapitel: Überwachung

Risikocluster: **Fehlerhafte Weiterverordnung von Medikamenten** (z.B. ohne Überprüfung der Reaktionen/Interaktionen, fehlende oder falsche Verordnung oder fortgesetzte Verordnung, fehlende oder falsche Beschriftung, fehlende Weiterverordnung vor Wochenenden oder Feiertagen, die Weiterverordnung von Medikamenten wurde nicht gestoppt).

Wie schätzen Sie die **Eintrittswahrscheinlichkeit** von diesem Risikocluster im Krankenhaus ein? Kreuzen Sie bitte an:

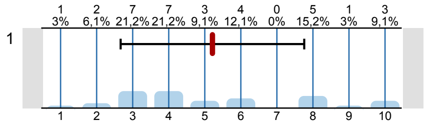

n=33
av.=5,2
dev.=2,5

Wie schätzen Sie die **Auswirkungen** dieses Risikoclusters für Patient*innen im Krankenhaus ein? Kreuzen Sie bitte an:

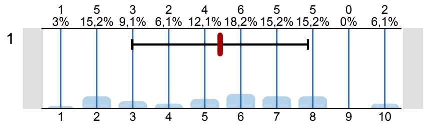

n=33
av.=5,4
dev.=2,4

Haben Sie Anmerkungen?

- .
- Durch eFK verbessert
- V.a. am OP-Tag werden Medika nicht eindeutig pausiert bzw. danach nicht wieder korrekt eingeleitet.
- Wird von Pflege sehr genau kontrolliert, da dies sehr oft vorkommt- hoher Zeitaufwand

Risikocluster: **Mangelhafte Kommunikation zwischen den Mitarbeiter*innen** (z.B. zwischen Pflegepersonen oder Ärzt*innen hinsichtlich Medikation wie Wirkung, Nebenwirkung, Wechselwirkung, Anordnung, Änderungen sowie Weitergabe von falschen Informationen).

Wie schätzen Sie die **Eintrittswahrscheinlichkeit** von diesem Risikocluster im Krankenhaus ein? Kreuzen Sie bitte an:

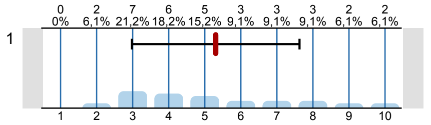

n=33
av.=5,3
dev.=2,3

Wie schätzen Sie die **Auswirkungen** dieses Risikoclusters für Patient*innen im Krankenhaus ein? Kreuzen Sie bitte an:

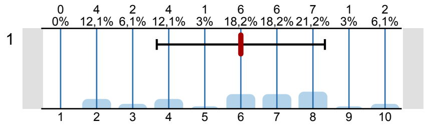

n=33
av.=6
dev.=2,3

Haben Sie Anmerkungen?

- .
- Durch eFK verbessert
- Personenabhängig, meist gute Kommunikation

Kapitel: Entlassung

Risikocluster: **Mangelnde Kommunikation/Information zwischen Krankenhaus und niedergelassenen Ärzt*innen, Pflegediensten und anderen Gesundheitsdienstanbieter*innen**, hinsichtlich Medikationsbedarf bei Entlassung; elektronische Kommunikation, Schnittstellenproblematik.

Wie schätzen Sie die **Eintrittswahrscheinlichkeit** von diesem Risikocluster im Krankenhaus ein? Kreuzen Sie bitte an:

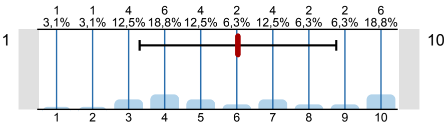

n=32
av.=6
dev.=2,7

Wie schätzen Sie die **Auswirkungen** dieses Risikoclusters für Patient*innen im Krankenhaus ein? Kreuzen Sie bitte an:

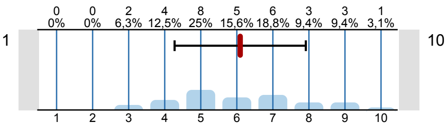

n=32
av.=6,1
dev.=1,8

Haben Sie Anmerkungen?

- .
- Gleiche Problematik wie eingangs mit e-Medikation (nur halt jetzt in die andere Richtung); tlw. unreflektierte Übernahme der KH-Medikation in den Arztbrief (?);
- Jeder Patient bekommt einen Arztbrief mit Medikation mit, bei Transferierungen oder Hauskrankenpflege auch Pflege/Transferbericht
- keine Angabe möglich

Risikocluster: **Fehlende Kommunikation/Information mit Patient*innen und Angehörigen** (z.B. Medikationsbedarf, Medikamente werden nicht erklärt).

Wie schätzen Sie die **Eintrittswahrscheinlichkeit** von diesem Risikocluster im Krankenhaus ein? Kreuzen Sie bitte an:

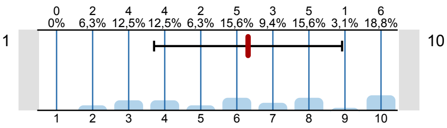

n=32
av.=6,3
dev.=2,6

Wie schätzen Sie die **Auswirkungen** dieses Risikoclusters für Patient*innen im Krankenhaus ein? Kreuzen Sie bitte an:

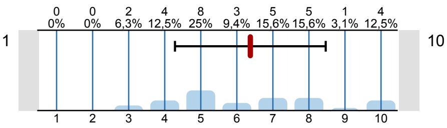

n=32
av.=6,4
dev.=2,1

Haben Sie Anmerkungen?

- Die Eintrittswahrscheinlichkeit ist meiner Einschätzung nach v.a. deswegen so hoch, da sich viele PatientInnen nicht an die Medikationsaufklärung erinnern können, und nicht weil keine Aufklärung durchgeführt worden ist.
- Sehr unterschiedlich, in meinem Bereich flächendeckend umgesetzt (Pat. ist geschult, informiert und hat Medika für mind. 2 Tage bzw. Rezepte bei Entlassung)
- keine Angabe möglich

Risikocluster: **Fehlende Verfügbarkeit von Medikamenten im niedergelassenen Bereich nach einem stationären Aufenthalt** (z.B. Originalpräparate vs. Generika).

Wie schätzen Sie die **Eintrittswahrscheinlichkeit** von diesem Risikocluster im Krankenhaus ein? Kreuzen Sie bitte an:

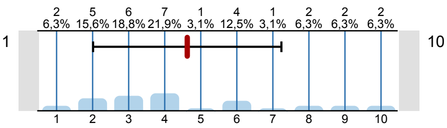

n=32
av.=4,6
dev.=2,6

Wie schätzen Sie die **Auswirkungen** dieses Risikoclusters für Patient*innen im Krankenhaus ein? Kreuzen Sie bitte an:

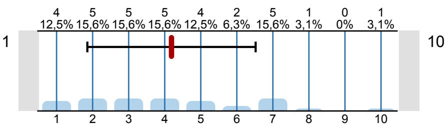

n=32
av.=4,2
dev.=2,3

Haben Sie Anmerkungen?

- .
- Generika sind weniger das Problem, sondern die fehlende chefärztliche Bewilligung (diese ging früher schneller durch, als jetzt elektronisch...??)

- Verschreibungsrichtlinien müssen natürlich eingehalten werden!
- keine Angabe möglich
- sehr unterschiedlich, in meinem Bereich erhalten Pat. neue Medikamenten für 2 Tage mit nach Hause bzw. bekommen ein Rezept (seltener)

Risikocluster: **Mangelhafte/Fehlerhafte schriftliche Entlassungsinformation** („Arztbrief“) (z.B. fehlende/unvollständige Verordnung der Medikamente, empfohlene Medikamente sind unvollständig, der Entlassungsbrief erreicht Patient*in, Hausarzt*in, Facharzt*in, Pflegeheim nicht oder verspätet).

Wie schätzen Sie die **Eintrittswahrscheinlichkeit** von diesem Risikocluster im Krankenhaus ein? Kreuzen Sie bitte an:

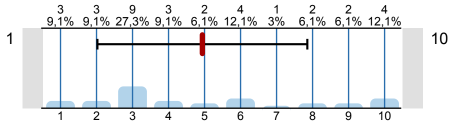

n=33
av.=4,9
dev.=2,9

Wie schätzen Sie die **Auswirkungen** dieses Risikoclusters für Patient*innen im Krankenhaus ein? Kreuzen Sie bitte an:

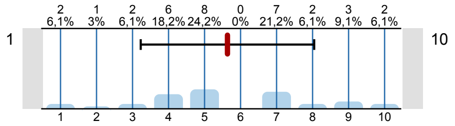

n=33
av.=5,6
dev.=2,4

Haben Sie Anmerkungen?

- .
- Entlassungen mit Arztbrief sind bereits Standard
- In meinem Bereich Entlassung nur mit Arztbrief und vereinbarten Ambulanzterminen
- siehe 6.3

Risikocluster: **Fehlendes Entlassungs- Assessment** hinsichtlich der weiteren (pflegerischen) Bedürfnisse von Patient*innen (z.B. Patient*innen sind nicht in der Lage Medikamente selbst zu organisieren, haben Probleme Medikamente zu bezahlen, wohnen fernab von Hausärzt*innen/Apotheken in einer abgelegenen Umgebung).

Wie schätzen Sie die **Eintrittswahrscheinlichkeit** von diesem Risikocluster im Krankenhaus ein? Kreuzen Sie bitte an:

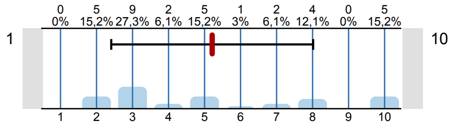

n=33
av.=5,2
dev.=2,8

Wie schätzen Sie die **Auswirkungen** dieses Risikoclusters für Patient*innen im Krankenhaus ein? Kreuzen Sie bitte an:

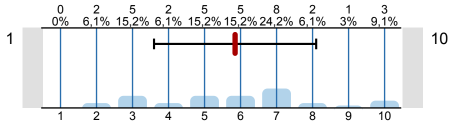

n=33
av.=5,8
dev.=2,3

Haben Sie Anmerkungen?

- Wird derzeit nicht standardmäßig erfragt und auch nicht standardmäßig in den Arztbrief geschrieben.
- Zeitmangel
- sehr unterschiedlich, in meinem Bereich ist das pflegerische Entlassungsmanagement sehr gut umgesetzt, Sozialarbeiter ist etabliert, Entlassungsberatungen zu unterschiedlichsten Themen sind etabliert, Angehörige werden immer einbezogen

Kapitel: Gesundheitspersonal (Kompetenz)

Risikocluster: **Problematische Umgebung während der einzelnen Schritte des Medikationsprozesses** (z.B. Lärm, schlechte Beleuchtung, Notfälle, chaotische Arbeitsumgebung, Unterbrechung/Ablenkung und hohe Arbeitsbelastung des Personals z.B. durch Unterbesetzung, schlechte Ausstattung der Station).

Wie schätzen Sie die **Eintrittswahrscheinlichkeit** von diesem Risikocluster im Krankenhaus ein? Kreuzen Sie bitte an:

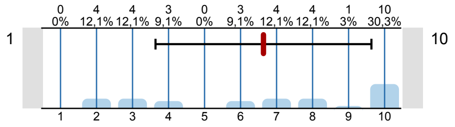

n=33
av.=6,6
dev.=3

Wie schätzen Sie die **Auswirkungen** dieses Risikoclusters für Patient*innen im Krankenhaus ein? Kreuzen Sie bitte an:

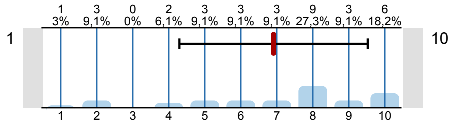

n=33
av.=6,9
dev.=2,6

Haben Sie Anmerkungen?

- .
- trifft sicher auf viele Bereiche zu

Risikocluster: **Wissensbasierte Fehler und mangelnde Ausbildung/Erfahrung** (z.B. fehlendes Wissen, Mangel an qualifiziertem Personal, Arbeit mit unerfahrenen oder neuen Mitarbeiter*innen, häufiger Personalwechsel sowie fehlende Ausbildung/Einschulung der am Medikationsprozess beteiligten Personen).

Wie schätzen Sie die **Eintrittswahrscheinlichkeit** von diesem Risikocluster im Krankenhaus ein? Kreuzen Sie bitte an:

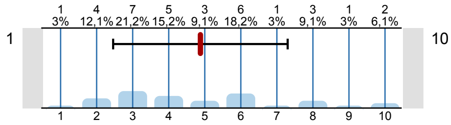

n=33
av.=4,9
dev.=2,4

Wie schätzen Sie die **Auswirkungen** dieses Risikoclusters für Patient*innen im Krankenhaus ein? Kreuzen Sie bitte an:

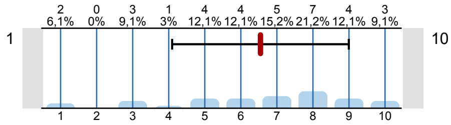

n=33
av.=6,5
dev.=2,5

Haben Sie Anmerkungen?

- .

Risikocluster: **Mangelndes Einhalten von Vorgaben/Richtlinien** hinsichtlich des Medikationsprozesses von Mitarbeiter*innen sowie Fehlen von Richtlinien und Standards im Krankenhaus zum Medikationsprozess.

Wie schätzen Sie die **Eintrittswahrscheinlichkeit** von diesem Risikocluster im Krankenhaus ein? Kreuzen Sie bitte an:

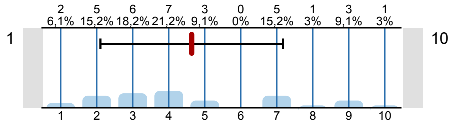

n=33
av.=4,6
dev.=2,5

Wie schätzen Sie die **Auswirkungen** dieses Risikoclusters für Patient*innen im Krankenhaus ein? Kreuzen Sie bitte an:

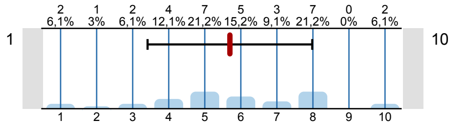

n=33
av.=5,7
dev.=2,3

Haben Sie Anmerkungen?

- .
- 4- Augenprinzip und Read back- Methode nicht umsetzbar- hohe Arbeitsdichte und nicht ausreichend Pflegepersonal

Risikocluster: **Mangelnde Gesundheit der Mitarbeiter*innen** (z.B. Müdigkeit, körperliche Erschöpfung, Stress) sowie **problematische Persönlichkeiten** (z.B. mangelnde Fehlerwahrnehmung und Gewissenhaftigkeit, Selbstgefälligkeit)

Wie schätzen Sie die **Eintrittswahrscheinlichkeit** von diesem Risikocluster im Krankenhaus ein? Kreuzen Sie bitte an:

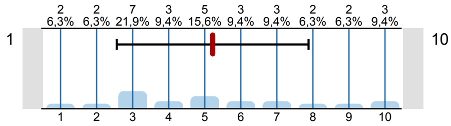

n=32
av.=5,2
dev.=2,7

Wie schätzen Sie die **Auswirkungen** dieses Risikoclusters für Patient*innen im Krankenhaus ein? Kreuzen Sie bitte an:

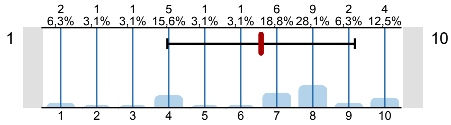

n=32
av.=6,6
dev.=2,6

Haben Sie Anmerkungen?

- .
- keine Angabe möglich

Risikocluster: **Allgemeine Kommunikationsschwierigkeiten zwischen dem Gesundheitspersonal** über den gesamten Medikationsprozess, Mängel in der Kommunikationsstruktur/im Informationsfluss, mangelnde **„Speaking up" Kultur** (Äußern von z.B. Sicherheitsmängel gegenüber anderen Mitarbeiter*innen), **schlechte lokale Arbeitskultur** sowie mangelnde Supervision/Begleitung durch leitende Kolleg*innen.

Wie schätzen Sie die **Eintrittswahrscheinlichkeit** von diesem Risikocluster im Krankenhaus ein? Kreuzen Sie bitte an:

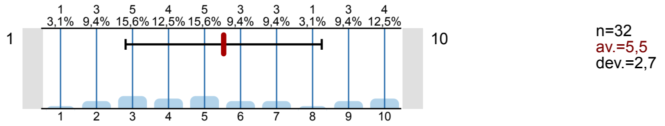

Wie schätzen Sie die **Auswirkungen** dieses Risikoclusters für Patient*innen im Krankenhaus ein? Kreuzen Sie bitte an:

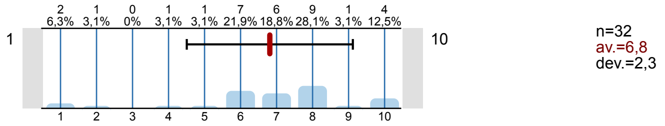

Haben Sie Anmerkungen?

- Dieser Risikocluster ist sehr stark von dem Team und dem Klima der jeweiligen Station abhängig.
- keine Angabe möglich
- sehr unterschiedlich wie Fehlerkultur gelebt wird- Personenabhängig aber generell Verbesserung

Kapitel: Patient*innen und Angehörige

Risikocluster: **Compliance von pflegenden Angehörigen/Verwandten in Bezug auf die Medikation** (z.B. pflegende Angehörige verstehen die Informationen zur Medikation nicht; Angehörige bringen ohne Rücksprache zusätzliche Medikamente mit; Angehörige verabreichen Medikamente ohne Rücksprache).

Wie schätzen Sie die **Eintrittswahrscheinlichkeit** von diesem Risikocluster im Krankenhaus ein? Kreuzen Sie bitte an:

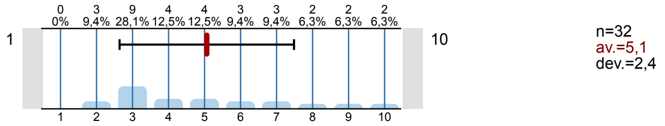

Wie schätzen Sie die **Auswirkungen** dieses Risikoclusters für Patient*innen im Krankenhaus ein? Kreuzen Sie bitte an:

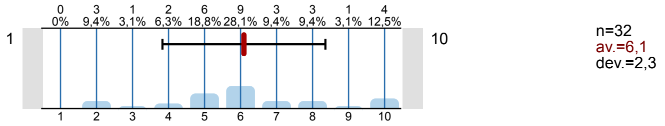

Haben Sie Anmerkungen?

- .
- keine Angabe möglich
- sehr selten der Fall

Risikocluster: **Risikofaktoren rund um die Patient*innen** (z.B. fehlende Compliance, mangelnde Gesundheitskompetenz, mangelndes Wissen zu den eigenen Medikamenten, fehlerhafte Einnahme von Medikamenten).

Wie schätzen Sie die **Eintrittswahrscheinlichkeit** von diesem Risikocluster im Krankenhaus ein? Kreuzen Sie bitte an:

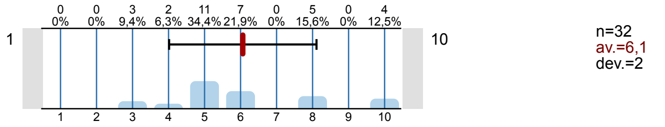

Wie schätzen Sie die **Auswirkungen** dieses Risikoclusters für Patient*innen im Krankenhaus ein? Kreuzen Sie bitte an:

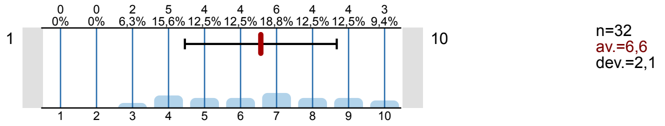

Haben Sie Anmerkungen?

- .
- keine Angabe möglich

Kapitel: Digitaler Prozess und IT-Sicherheit

Risikocluster: **Fehlende/mangelhafte Hardware** (z.B. fehlende Ausstattung zur Nutzung von elektronischen Systemen, veraltete Geräte, schlechte WLAN- Technologie).

Wie schätzen Sie die **Eintrittswahrscheinlichkeit** von diesem Risikocluster im Krankenhaus ein? Kreuzen Sie bitte an:

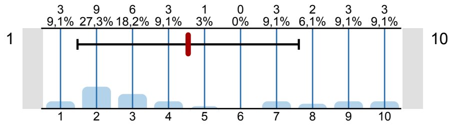

n=33
av.=4,5
dev.=3,1

Wie schätzen Sie die **Auswirkungen** dieses Risikoclusters für Patient*innen im Krankenhaus ein? Kreuzen Sie bitte an:

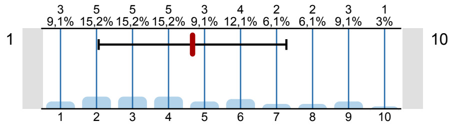

n=33
av.=4,7
dev.=2,6

Haben Sie Anmerkungen?

- .
- Nur weil es langsamer/umständlicher geht, heißt es noch lange nicht, dass das Ergebnis schlechter ist - Fehler weniger eindeutig hardwareseitig, meist ein UX-Problem oder User*innen-Problem, weil die mangelhafte Hardware zu unsorgfältigem Arbeiten (Workarounds, Abkürzungen...) verleitet.
- Ohne technische Ausstattung kein gesicherter Prozess!
- Performance eFK viel zu langsam!!!!

Risikocluster: **Mangelhafte Software** (inkludiert die gesamte Gesundheitsinformationstechnologie im Krankenhaus), **fehlende/ schlecht gestaltete Schnittstellen zwischen Programmen, die im Medikationsprozess verwendet werden** (z.B. bei Aufnahme, Verordnung, Bestellung, Bestand, Entlassung), schlechte Benutzerfreundlichkeit, unvorhergesehene Interaktion von Systemen, Softwarefehler, fehlende Sicherheit.

Wie schätzen Sie die **Eintrittswahrscheinlichkeit** von diesem Risikocluster im Krankenhaus ein? Kreuzen Sie bitte an:

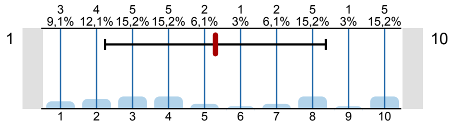

n=33
av.=5,3
dev.=3,1

Wie schätzen Sie die **Auswirkungen** dieses Risikoclusters für Patient*innen im Krankenhaus ein? Kreuzen Sie bitte an:

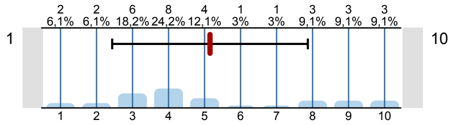

n=33
av.=5,2
dev.=2,7

Haben Sie Anmerkungen?

- .
- Die Schnittstelle zwischen Apotheke (Einkauf) und der Abgleich mit der Medikationsdatenbank gehört dringenst reformiert. Die ‚Pflege ‘ der Medikationsdatenbank gehört ebenfalls professionalisiert und standardisiert!!!!!‘

Risikocluster: **Mängel in der Implementierung von Gesundheitsinformationstechnologien** (z.B. schlechte Implementierungspläne, Ausbildung und Stärkung der IT-Kompetenzen von Gesundheitspersonal sind nicht erfolgt).

Wie schätzen Sie die **Eintrittswahrscheinlichkeit** von diesem Risikocluster im Krankenhaus ein? Kreuzen Sie bitte an:

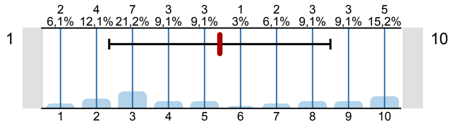

n=33
av.=5,4
dev.=3,1

Wie schätzen Sie die **Auswirkungen** dieses Risikoclusters für Patient*innen im Krankenhaus ein? Kreuzen Sie bitte an:

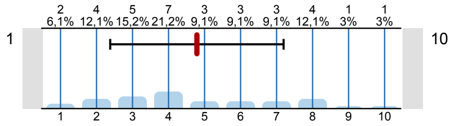

n=33
av.=4,8
dev.=2,4

Haben Sie Anmerkungen?

- v.a. nicht Technik-versierte KollegInnen tun sich mit digitalen Geräten schwer und/oder benötigen dadurch länger. (z.B. Schreiben mit einer Tastatur)

Allgemeine Kommentare

Möchten Sie uns noch etwas ergänzend mitteilen? Fehlt Ihnen ein Risikocluster?

- .
- Der Fragebogen listet zu einem erheblichen Teil Ursachen für Risiken in der Medikation und nicht die Risiken selbst. Die Bewertung der Eintrittswahrscheinlichkeit und insbesondere der Auswirkungen, die zur Einschätzung von Risiken verwendet wird, ist für Ursachen ungeeignet! Die Vermischung von Ursachen und Risiken unter dem Begriff Cluster macht eine sinnvolle Bewertung so gut wie unmöglich - überdenken Sie bitte die Methode!
- Interklinische Schnittstellen
- Nein, ich finde die Darstellung der Cluster aus meiner Sicht ganz vollständig.
- Sehr, sehr wichtiges Projekt!!!

Vielen Dank für Ihre Mitarbeit!
