# Supplementary material for: Risks in the analogue and digitally-supported medication process and potential solutions to increase patient safety in the hospital: A mixed methods study
Source: PLoS One. 2024 Feb 27;19(2):e0297491. doi: 10.1371/journal.pone.0297491 (PMC10898776; doi:10.1371/journal.pone.0297491)
Supplement: S2 File — Befragung. (PDF) [file pone.0297491.s003.pdf]

Wissenschaftliche Projekte

MeDi-Pro 2. Befragung ()
No. of responses = 28

Legend

Question text

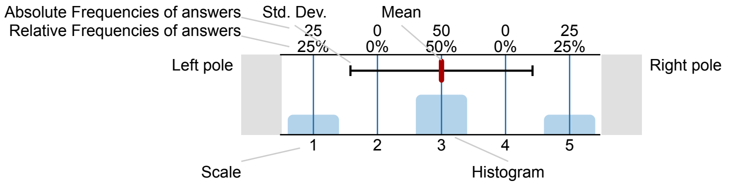

n=No. of responses
av.=Mean
dev.=Std. Dev.
ab.=Abstention

Teil 1: Fragen zur Person

Geschlecht

n=28

Weiblich	64.3%
Männlich	35.7%
Divers	0%
Keine Angabe	0%

Alter

n=28

18-30 Jahre	17.9%
31-40 Jahre	21.4%
41-50 Jahre	25%
51-60 Jahre	32.1%
≥ 61 Jahre	3.6%

Beruf (in dem Sie vorwiegend arbeiten)

n=28

Pflegeperson	32.1%
Arzt/Ärztin	28.6%
Qualitätsmanagement/Risikomanagement	25%
Management	7.1%
Sonstiges:	7.1%

Berufserfahrung

n=28

0-2 Jahre	7.1%
3-6 Jahre	10.7%
7-10 Jahre	14.3%
11-20 Jahre	25%
>20 Jahre	42.9%

Code zur Wiedererkennung in der 2. Delpi-Befragung

Geburtsjahr Mutter z.B. 1955

- 1920
- 1922
- 1930

- 1932
- 1936
- 1938
- 1939
- 1940
- 1941 (2 Counts)
- 1944 (2 Counts)
- 1948
- 1951
- 1952
- 1953
- 1954
- 1955
- 1956
- 1959
- 1963 (2 Counts)
- 1965
- 1966
- 1967
- 1969

---

1. Buchstabe Vorname Mutter

- A
- B (2 Counts)
- C (2 Counts)
- E (2 Counts)
- G
- H (3 Counts)
- I (3 Counts)
- R (4 Counts)
- S (2 Counts)
- W (2 Counts)
- a
- c
- h (2 Counts)
- m

---

1. Buchstabe Vorname Vater

- A (4 Counts)
- F (4 Counts)

- G (3 Counts)
- H (2 Counts)
- J (2 Counts)
- K
- R (5 Counts)
- S
- a
- e
- g
- k
- w

Allgemeine Informationen

Wir ersuchen Sie, die Top 15 Risikocluster hinsichtlich Potential von Lösungen in der Stärkung der Mitarbeiter*innen-Kompetenz sowie Potential von Lösungen im Bereich Digitalisierung zu bewerten. Zur Bewertung der Lösungspotentiale der Risikocluster steht Ihnen wieder eine Skala von 1-10 zur Verfügung, wobei 1 für ein sehr geringes und 10 für ein sehr hohes Lösungspotential stehen.

Bei jedem Risikocluster haben Sie auch die Möglichkeit, einen konkreten Lösungsvorschlag in Form eines Kommentars abzugeben.

Ziel der Befragung ist es, mögliche Lösungsansätze für Risiken im Medikationsprozess zu identifizieren, die in der Zukunft einen Einsatz in der Praxis finden können.

Top Risiken - Aufnahme

Aufnahme: Unzureichende Kommunikation über die verordneten Medikamente zwischen dem niedergelassenen Bereich und dem Krankenhaus.

Wie hoch schätzen Sie das Potential einer Lösung durch die **Stärkung der Mitarbeiter*innen-Kompetenz** (z.B. Aus- und Weiterbildung, etc.) für diesen Risikocluster im Krankenhaus ein?

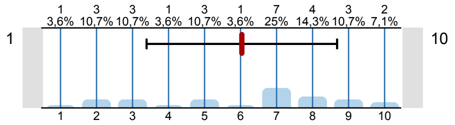

n=28
av.=6
dev.=2,6

Wie hoch schätzen Sie das Potential einer Lösung im **Bereich Digitalisierung** (z.B. neue Soft- und Hardwarelösungen, etc.) für diesen Risikocluster im Krankenhaus ein?

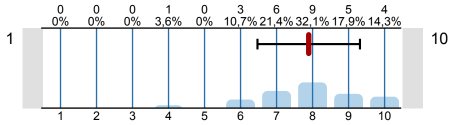

n=28
av.=7,9
dev.=1,4

Haben Sie einen konkreten Lösungsvorschlag für eines der genannten Risiken aus diesem Risikocluster?

- Aktuelle Digitalisierung schlecht umgesetzt und nur wenig praktikabel (ELGA); Neuentwicklung notwendig
- Eingabe der Dauermedikation in die ELGA durch den Hausarzt
- Elektronische "Liste" der verschriebenen Medikamente
- In ELGA auch Dosierungen eingeben, verpflichtende Eingabe alle Medika in ELGA durch verschreibende ÄrztInnen
- Sensibilisierung der MA auf Kommunikation mit externen Bereichen; zusätzliche Erleichterung der Erkennung möglicher Risikofaktoren und der verordneten Medikamente durch eine digitales Tool
- Vernetzung Hausarzt- KH stärken, ELGA ist ein erster Schritt

Aufnahme: Unvollständige Medikationsliste bei Aufnahme mit Diskrepanzen in der Medikamentenhistorie (z.B. unterschiedliche Listen von Patient*in, Hausarzt*in, Facharzt*in, elektronischer Medikamentenerfassung).

Wie hoch schätzen Sie das Potential einer Lösung durch die **Stärkung der Mitarbeiter*innen-Kompetenz** (z.B. Aus- und Weiterbildung, etc.) für diesen Risikocluster im Krankenhaus ein?

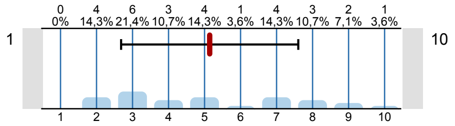

n=28
av.=5,1
dev.=2,5

Wie hoch schätzen Sie das Potential einer Lösung im **Bereich Digitalisierung** (z.B. neue Soft- und Hardwarelösungen, etc.) für diesen Risikocluster im Krankenhaus ein?

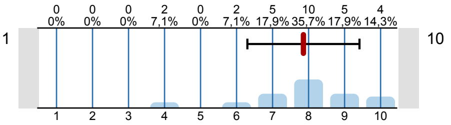

n=28
av.=7,9
dev.=1,6

Haben Sie einen konkreten Lösungsvorschlag für eines der genannten Risiken aus diesem Risikocluster?

- Durch eFK werden sich wahrscheinlich Verbesserungen ergeben, tws Lösungsansätze bereits vorhanden, aktuelle Digitalisierung schlecht umgesetzt und nur wenig praktikabel (ELGA); Neuentwicklung notwendig
- ELGA? Verpflichtend für solche Daten?
- Elektronische "Liste" der verschriebenen Medikamente mit Gültigkeitsdatum
- Medikationsplan mit QR-Code zum Einscannen in Krankenhausinformationssystem
- Übertragung der Entlassungs-eMedikation KH in ELGA

Aufnahme: Herausforderung bei Medikamenten (Polypharmazie (definiert als mehr als 5 Medikamente), Generika vs. Originator, Hochrisikomedikamente, Wechselwirkungen von Medikamenten).

Wie hoch schätzen Sie das Potential einer Lösung durch die **Stärkung der Mitarbeiter*innen-Kompetenz** (z.B. Aus- und Weiterbildung, etc.) für diesen Risikocluster im Krankenhaus ein?

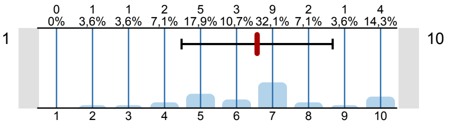

n=28
av.=6,6
dev.=2,1

Wie hoch schätzen Sie das Potential einer Lösung im **Bereich Digitalisierung** (z.B. neue Soft- und Hardwarelösungen, etc.) für diesen Risikocluster im Krankenhaus ein?

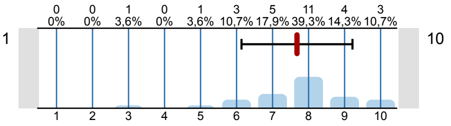

n=28
av.=7,7
dev.=1,5

Haben Sie einen konkreten Lösungsvorschlag für eines der genannten Risiken aus diesem Risikocluster?

- Automatische Überprüfung von Wechselwirkungen mit "intelligenten" Algorithmen, nur Warnung vor Wechselwirkungen nicht ausreichend
- Jeder Patient mit mehr als 5 Medikamenten könnte eine Evaluierung seiner Medikation bekommen
- Pharmazeut vor Ort, der beratend tätig ist und weiß, welche Medikamente gerade vorhanden sind
- Softwarelösung - die im Hintergrund zu der elektronischen Medikamentenliste mitläuft!
- automatisierte Darstellung von potentiellen Risiken (Doppelverordnung, Falschdosierung..)
- digitale Warnsysteme, vermehrte Medikamentenschulungen der Ärzte,

Aufnahme: Allergiefehler - Allergien werden nicht erhoben, nicht oder falsch dokumentiert oder nicht berücksichtigt, keine Angabe einer Allergie durch Patient*innen.

Wie hoch schätzen Sie das Potential einer Lösung durch die **Stärkung der Mitarbeiter*innen-Kompetenz** (z.B. Aus- und Weiterbildung, etc.) für diesen Risikocluster im Krankenhaus ein?

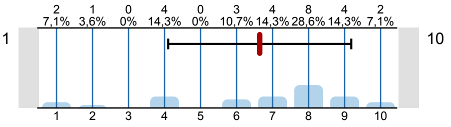

n=28
av.=6,6
dev.=2,5

Wie hoch schätzen Sie das Potential einer Lösung im **Bereich Digitalisierung** (z.B. neue Soft- und Hardwarelösungen, etc.) für diesen Risikocluster im Krankenhaus ein?

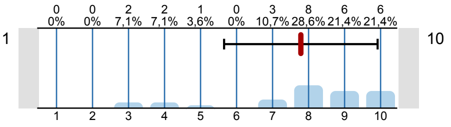

n=28
av.=7,8
dev.=2,1

Haben Sie einen konkreten Lösungsvorschlag für eines der genannten Risiken aus diesem Risikocluster?

- Allergien werden in die ELGA durch den Hausarzt eingegeben
- Elektronische "Liste" der verschriebenen Medikamente und Allergien
- Wenn Briefe nur freigegeben werden können wenn im Feld Allergien ein Eintrag oder Hinweis "keine Allergie" erfolgt ist
- bei eMedikation: Verordnung von Medis bei Allergie- lässt System nicht einfach zu- gut sichtbare Warnung
- digitale Abfragen und Warnsysteme, vermehrte Medikamentenschulungen des Personals

- zentrale Datenerhebung z.B durch Allgemeinmedizin und zentrale Anpassung der Daten

Top Risiken - Verordnung

Verordnung: Allgemeine Fehler in der Verordnung (z.B. falsches Medikament, falsche Dosis, unvollständige Verordnung und andere Fehlertypen wie Auslassungsfehler, Übertragungsfehler, Duplizierungsfehler).

Wie hoch schätzen Sie das Potential einer Lösung durch die **Stärkung der Mitarbeiter*innen-Kompetenz** (z.B. Aus- und Weiterbildung, etc.) für diesen Risikocluster im Krankenhaus ein?

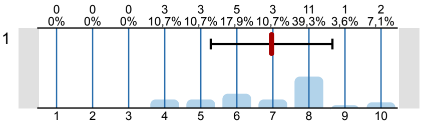

n=28
av.=7
dev.=1,7

Wie hoch schätzen Sie das Potential einer Lösung im **Bereich Digitalisierung** (z.B. neue Soft- und Hardwarelösungen, etc.) für diesen Risikocluster im Krankenhaus ein?

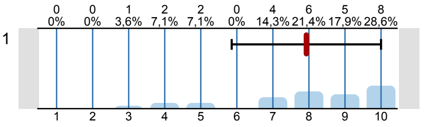

n=28
av.=7,9
dev.=2,1

Haben Sie einen konkreten Lösungsvorschlag für eines der genannten Risiken aus diesem Risikocluster?

- Eine gute Software für eine E-Fieberkurve und Möglichkeit der Medikamenteneingabe im ambulanten Arztbrief über Medis wäre hilfreich.
  Leider können jetzt die Medikamentenlisten andere Kliniken nicht kopiert und eingesetzt werden z.B. die Medika der Hämatologie sind in den Arztbriefen sehr übersichtlich dargestellt können aber nicht kopiert und im eigenen Arztbrief eingesetzt werden.
- Medikament muss in der digitalen Fieberkurve in der eichtigen Dosis angeklickt werden
- Medikamentenverordnungen von Hausärzten sollten von Fachärzten überprüft werden oder bestimmte Medikamente nur von Fachärzten verordnet werden, Schulungen des Personals im KH macht nur teilweise Sinn, da nicht alle Medikamente die extern verschrieben wurden im KH nachträglich bei allen Patienten überarbeitet und umgestellt werden können, diese Verantwortung sollte bei den externen wahrgenommen werden
- e Medikation: System zeigt unvollständige Verordnung, Auslassungen usw. an

Verordnung: Schwierigkeiten mit der handschriftlichen Verordnung (z.B. unvollständige Verordnung, Unleserlichkeit der Verordnung, Verordnung mit Bleistift oder „nicht wasserfestem Stift“, Verwendung von Korrekturlack).

Wie hoch schätzen Sie das Potential einer Lösung durch die **Stärkung der Mitarbeiter*innen-Kompetenz** (z.B. Aus- und Weiterbildung, etc.) für diesen Risikocluster im Krankenhaus ein?

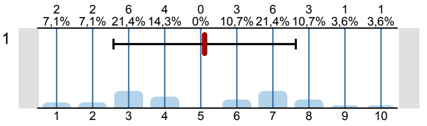

n=28
av.=5,1
dev.=2,5

Wie hoch schätzen Sie das Potential einer Lösung im **Bereich Digitalisierung** (z.B. neue Soft- und Hardwarelösungen, etc.) für diesen Risikocluster im Krankenhaus ein?

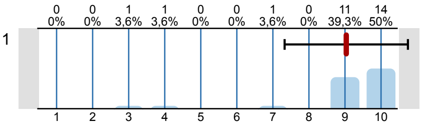

n=28
av.=9
dev.=1,7

Haben Sie einen konkreten Lösungsvorschlag für eines der genannten Risiken aus diesem Risikocluster?

- Implementierung der elektronischen Fieberkurve
- Lösung mit eFK vorhalnden
- digitale Fieberkurve lößt dieses Problem
- elektronische Unterschrift

Verordnung: Herausforderungen in der Verordnung von komplexen Medikamenten/Hochrisikomedikamenten (z.B. Polypharmazie, mangelnde Kontrolle der Arzneimittelwechselwirkungen) durch Mangel an klinisch-pharmakologischem Wissen (z.B. irrationale, unangemessene und ineffektive Verordnung) und/oder durch fehlende Verordnungsschemata oder Nicht-Verwendung vorhandener Verordnungsschemata.

Wie hoch schätzen Sie das Potential einer Lösung im **Bereich Digitalisierung** (z.B. neue Soft- und Hardwarelösungen, etc.) für diesen Risikocluster im Krankenhaus ein?

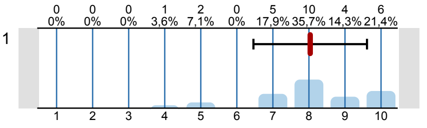

n=28
av.=8
dev.=1,6

Wie hoch schätzen Sie das Potential einer Lösung durch die **Stärkung der Mitarbeiter*innen-Kompetenz** (z.B. Aus- und Weiterbildung, etc.) für diesen Risikocluster im Krankenhaus ein?

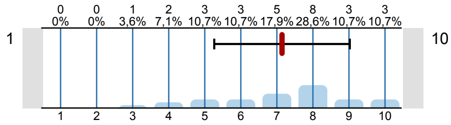

n=28
av.=7,1
dev.=1,9

Haben Sie einen konkreten Lösungsvorschlag für eines der genannten Risiken aus diesem Risikocluster?

- 4.8 Pharmazeut berät bei Polypharmazie- interaktives Gespräch und Diskussion manchmal nötig
  4.7 eMedikation: Verordnungsschemata sind hinterlegt, System warnt bei Fehlverordnungen..
- Fachinformation könnte mit einem Klick in der digitalen Fieberkurve aufgerufen werden
- Nicht jede Kontraindikation oder jeder Warnhinweis ist zu beachten. Oft liegt es an fehlenden STudien und Sicherheitsdenken (finanzielle Sichaheit im Schadensfall) der Pharmafirmen, dass Warnhinweise ausgestellt werden. Würde man nur mittels Computerprogramm die Warnhinweise beachten, können keine Kinder behandelt werden, da die viele häufig eingesetzte Medikament Off Level sind. Die medizinische Expertise kann durch einen Computer hier nur ergänzt werden. Die Entscheidung und Verantwortung liegt immer noch beim Arzt.
- Software-Lösung
- ganzheitliches Patientendenken bei der Medikamentenverordnung durch Ärzte fördern (nicht nur fachspezifisch), elektronische Warnhinweise
- siehe oben

Top Risiken - Überprüfung

Überprüfung: Fehlende Überprüfung/Unterstützung bei komplexen Verordnungen von (klinischen) Pharmazeut*innen (z.B. Hochrisikomedikamente, Polypharmazie, komplexe Indikationen und Diagnosen).

Wie hoch schätzen Sie das Potential einer Lösung durch die **Stärkung der Mitarbeiter*innen-Kompetenz** (z.B. Aus- und Weiterbildung, etc.) für diesen Risikocluster im Krankenhaus ein?

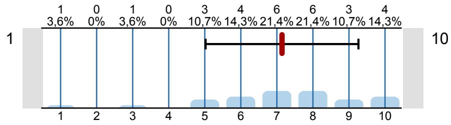

n=28
av.=7,1
dev.=2,1

Wie hoch schätzen Sie das Potential einer Lösung im **Bereich Digitalisierung** (z.B. neue Soft- und Hardwarelösungen, etc.) für diesen Risikocluster im Krankenhaus ein?

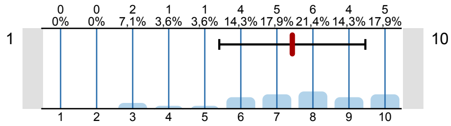

n=28
av.=7,4
dev.=2

Haben Sie einen konkreten Lösungsvorschlag für eines der genannten Risiken aus diesem Risikocluster?

- automatische Abfrage bei Patienten mit >5 Medikamenten, ob eine klinisch-pharmakologidche Prüfung angeordnet werden soll
- siehe vorne, Pharmazeuten sollten nicht in die Therapie von Ärzten eingreifen, es gibt nur sehr wenige Red Flags (z.b. tägliche MTX Einnahme) bei solchen Empfehlungen wäre ein Computer/Pharmazeut hilfreich. Es gibt aber nur sehr wenige schwere Kombinations- und Dosierungsfehler und hier muss die medizinische Ausbildung und Fortbildung eingreifen. Ärzte zusammen mit anderen Spezialisten Pharmazeuten... sollten gemeinsam festlegen was tatsächlich Red Flags sind. Nicht jeder kleine Warnhinweis ist auch therapeutisch relevant.

Top Risiken - Dispensieren

Dispensieren: Fehler bei der Vorbereitung/Dispensierung von Medikamenten (z.B. Fehler bei der Teilung der Tabletten, falsches Medikament, falsche Dosis, falsche Berechnung, fehlende oder falsche Änderung des angeordneten Medikaments im Dispenser, fehlende oder falsche Dokumentation, fehlende/falsche/unklare Beschriftung/Kennzeichnung vorbereiteter Medikamente).

Wie hoch schätzen Sie das Potential einer Lösung durch die **Stärkung der Mitarbeiter*innen-Kompetenz** (z.B. Aus- und Weiterbildung, etc.) für diesen Risikocluster im Krankenhaus ein?

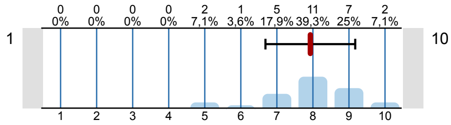

n=28
av.=7,9
dev.=1,2

Wie hoch schätzen Sie das Potential einer Lösung im **Bereich Digitalisierung** (z.B. neue Soft- und Hardwarelösungen, etc.) für diesen Risikocluster im Krankenhaus ein?

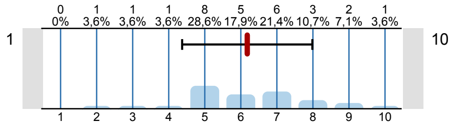

n=28
av.=6,2
dev.=1,8

Haben Sie einen konkreten Lösungsvorschlag für eines der genannten Risiken aus diesem Risikocluster?

- 4 Augenprinzip und ruhige Umgebung! Lesbare e-Fieberkurven sind aber sicher von Vorteil.
- Dispensierung auslagern- Medikation wird dispensiert zugestellt- nur Änderungen oder Neuanordnungen müssen dispensiert werden

(Fehlerminimierung und mehr Zeitressource für PatientInnenbetreuung)

- Sensibilisierung der MA wichtig, elektronische Hilfe der Fehlerminimierung: die elektronische Fieberkurve

Dispensieren: Verwechslung von Medikamenten (z.B. Fehler mit ähnlich aussehenden Medikamenten, Fehler mit ähnlich klingenden Medikamentennamen, Verwechslung von Medikamentennamen / -verpackungen).

Wie hoch schätzen Sie das Potential einer Lösung durch die **Stärkung der Mitarbeiter*innen-Kompetenz** (z.B. Aus- und Weiterbildung, etc.) für diesen Risikocluster im Krankenhaus ein?

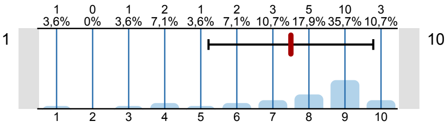

n=28
av.=7,5
dev.=2,3

Wie hoch schätzen Sie das Potential einer Lösung im **Bereich Digitalisierung** (z.B. neue Soft- und Hardwarelösungen, etc.) für diesen Risikocluster im Krankenhaus ein?

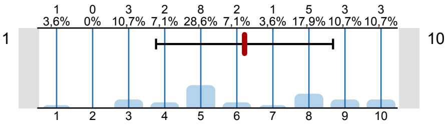

n=28
av.=6,2
dev.=2,5

Haben Sie einen konkreten Lösungsvorschlag für eines der genannten Risiken aus diesem Risikocluster?

- Dispensierung auslagern- Medikation wird dispensiert zugestellt- nur Änderungen oder Neuanordnungen müssen dispensiert werden (Fehlerminimierung und mehr Zeitressource für PatientInnenbetreuung)
- Look-a-likes aus dem Sortiment nehmen. Mitarbeiter trainieren, wenn sie Look-a-likes sehen, dass sie diese sofort melden/CIRSen bevor es zu einem Vorfall kommt.
- Sensibilisierung der MA
- Unit Dose
- Wenn alle Medika über Barcodescanner eingeschachtelt werden, gibt es sicher weniger Verwechslungen.

Dispensieren: Mangelnde Kommunikation/Missverständnisse in der Kommunikation (z.B. Fehler bei telefonischen Anordnungen, Missverständnisse in Bezug auf Medikamentennamen, Dosierung, Intervall, Dosierungsform, Patient*in).

Wie hoch schätzen Sie das Potential einer Lösung durch die **Stärkung der Mitarbeiter*innen-Kompetenz** (z.B. Aus- und Weiterbildung, etc.) für diesen Risikocluster im Krankenhaus ein?

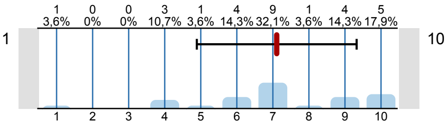

n=28
av.=7,1
dev.=2,2

Wie hoch schätzen Sie das Potential einer Lösung im **Bereich Digitalisierung** (z.B. neue Soft- und Hardwarelösungen, etc.) für diesen Risikocluster im Krankenhaus ein?

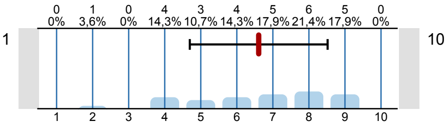

n=28
av.=6,6
dev.=1,9

Haben Sie einen konkreten Lösungsvorschlag für eines der genannten Risiken aus diesem Risikocluster?

- Sensibilisierung der MA, Freigabe der Medika durch den Arzt (elektronische FK)

Top Risiken - Verabreichung

Verabreichung: Fehler bei der Verabreichung von Medikamenten (z.B. falsches Medikament, falsche Dosierung, falscher Applikationsweg, Verwechslung von ähnlich aussehenden oder ähnlich klingenden Medikamenten, falsche Zeit der Verabreichung, nicht autorisierte Arzneimittel, Auslassungsfehler, fehlerhafte Überprüfungsaktivitäten, Schwierigkeiten mit Infusionsgeräten, Verwechslung der Verpackung der Medikamente, falsche Kennzeichnung des Medikaments auf der Verpackung).

Wie hoch schätzen Sie das Potential einer Lösung durch die **Stärkung der Mitarbeiter*innen-Kompetenz** (z.B. Aus- und Weiterbildung, etc.) für diesen Risikocluster im Krankenhaus ein?

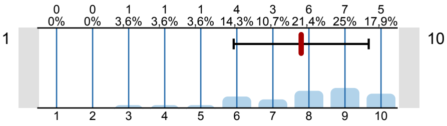

n=28
av.=7,8
dev.=1,9

Wie hoch schätzen Sie das Potential einer Lösung im **Bereich Digitalisierung** (z.B. neue Soft- und Hardwarelösungen, etc.) für diesen Risikocluster im Krankenhaus ein?

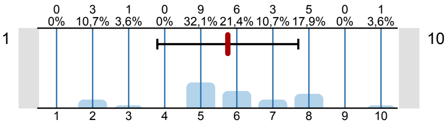

n=28
av.=5,8
dev.=2

Haben Sie einen konkreten Lösungsvorschlag für eines der genannten Risiken aus diesem Risikocluster?

- Elektronisches Warnzeichen bei "elektronischer" Überdosierung

- Sensibilisierung der MA, Bestätigung/Kontrolle eFK
- bei Infusionstherapie: ev. Scan Patientenidentifikationsband und Infusionsetikette zum Abgleich- richtiger Patient/ richtiger Zeitpunkt/ richtiges Medikament
- ein Barcode-Scan-System mit dem Armband könnte überlegt werden. Dies würde wahrscheinlich aber von den Patienten als unpersönlich empfunden werden.

Top Risiken - Entlassung

Entlassung: Fehlende Kommunikation/Information mit Patient*innen und Angehörigen (z.B. Medikationsbedarf, Medikamente werden nicht erklärt).

Wie hoch schätzen Sie das Potential einer Lösung durch die **Stärkung der Mitarbeiter*innen-Kompetenz** (z.B. Aus- und Weiterbildung, etc.) für diesen Risikocluster im Krankenhaus ein?

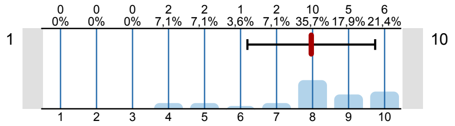

n=28
av.=8
dev.=1,8

Wie hoch schätzen Sie das Potential einer Lösung im **Bereich Digitalisierung** (z.B. neue Soft- und Hardwarelösungen, etc.) für diesen Risikocluster im Krankenhaus ein?

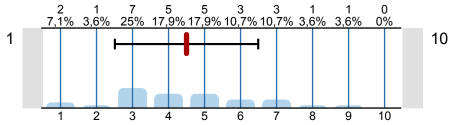

n=28
av.=4,5
dev.=2

Haben Sie einen konkreten Lösungsvorschlag für eines der genannten Risiken aus diesem Risikocluster?

- Digitale Verordnung (intra- und extramural)
- ELGA könnte viel hilfreicher sein, wenn ordentlich befüllt.
- Eingabe der Arztbriefe in die ELGA. Im Arztbrief fett markieren, wenn ein Medikament neu eingeleitet/beendet/veräbdert worden ist
- bei speziellen Medikamenten wie z. B. Immunsuppression- MedikamentenschulungsAPP zum Download für Patientinnen und Personal

Top Risiken - Kompetenzen

Kompetenzen: Problematische Umgebung während der einzelnen Schritte des Medikationsprozesses (z.B. Lärm, schlechte Beleuchtung, Notfälle, chaotische Arbeitsumgebung, Unterbrechung/Ablenkung und hohe Arbeitsbelastung des Personals z.B. durch Unterbesetzung, schlechte Ausstattung der Station).

Wie hoch schätzen Sie das Potential einer Lösung durch die **Stärkung der Mitarbeiter*innen-Kompetenz** (z.B. Aus- und Weiterbildung, etc.) für diesen Risikocluster im Krankenhaus ein?

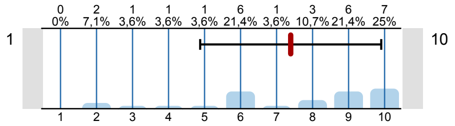

n=28
av.=7,4
dev.=2,5

Wie hoch schätzen Sie das Potential einer Lösung im **Bereich Digitalisierung** (z.B. neue Soft- und Hardwarelösungen, etc.) für diesen Risikocluster im Krankenhaus ein?

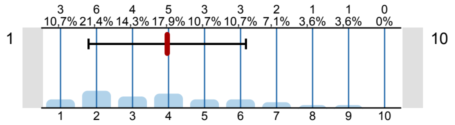

n=28
av.=4
dev.=2,2

Haben Sie einen konkreten Lösungsvorschlag für eines der genannten Risiken aus diesem Risikocluster?

- Umgebung anpassen, MA schulen/sensibilisieren
- Wenn Mitarbeiter Medikamente bereit stellen, so sollten sie nur für diese Aufgabe freigespielt werden. Ich habe einmal in Deutschland gesehen, dass Mitarbeiter sogar Warnwesten trugen... als Erkennung... hinten stand sogar irgendwas in der Richtung drauf wie... "Sprich mich nur im Notfall an, ich stelle gerade Medikamente bereit"
- Wissen um die fehlerbegünstigenden Faktoren schärfen, Maßnahmen zur Reduktion (geschützter Raum etc)
- mehr Personal. mehr Crisis-Ressource-Management. Verwendung von Checklisten und Ausbildung der Mitarbeiter in der Verwendung von Checklisten.

Top Risiken - Patient*innen und Angehörige

Patient*innen und Angehörige: Risikofaktoren rund um die Patient*innen (z.B. fehlende Compliance, mangelnde Gesundheitskompetenz, mangelndes Wissen zu den eigenen Medikamenten, fehlerhafte Einnahme von Medikamenten).

Wie hoch schätzen Sie das Potential einer Lösung durch die **Stärkung der Mitarbeiter*innen-Kompetenz** (z.B. Aus- und Weiterbildung, etc.) für diesen Risikocluster im Krankenhaus ein?

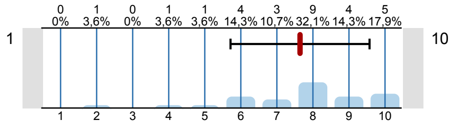

n=28
av.=7,6
dev.=1,9

Wie hoch schätzen Sie das Potential einer Lösung im **Bereich Digitalisierung** (z.B. neue Soft- und Hardwarelösungen, etc.) für diesen Risikocluster im Krankenhaus ein?

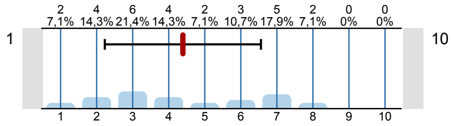

n=28
av.=4,4
dev.=2,2

Haben Sie einen konkreten Lösungsvorschlag für eines der genannten Risiken aus diesem Risikocluster?

- Aufklärung muss erfolgen
- Infoblätter für Patienten in verständlicher Sprache. Medikalisten ausgedruckt für die Brieftasche mitgeben.
- Kommunikationsförderung MA-Patient
- Persönlich einen Zugang zum Patienten finden (Empathie). Herausfinden des Willen des Patienten und nicht auf die sozial erwünschte Antworten vertrauen.
- Schulung MA um gute Patientenedukation durchführen zu können (z. B. sinnvolle Gesprächstechniken wie Motivational Interviewing) MedikamentenAPP für spezielle Medikamentengruppen (z. B.: Immunos, orale Antikoagulation usw.)

Allgemeine Kommentare

Möchten Sie uns etwas ergänzend mitteilen?

- Awareness ist meines Erachtens in diesem Kontext das Schlagwort schlechthin.
- Schulungen der externen Ärzte würde aus meiner Erfahrung nach Sinn machen, da Patienten oft mit sehr langen Medikamentenlisten im KH aufgenommen werden, da jeder Arzt ein neues Medikament verordnet und nicht ganzheitlich der Patient betrachtet und auf Aufhebungen der einzelnen Produkte geachtet wird. Auch die Verordnung von spezielleren Medikamenten sollte nicht für jeden Mediziner möglich sein.

Vielen Dank für Ihre Mitarbeit!
