# Supplementary material for: Risks in the analogue and digitally-supported medication process and potential solutions to increase patient safety in the hospital: A mixed methods study
Source: PLoS One. 2024 Feb 27;19(2):e0297491. doi: 10.1371/journal.pone.0297491 (PMC10898776; doi:10.1371/journal.pone.0297491)
Supplement: S3 File — Survey (English). (PDF) [file pone.0297491.s004.pdf]

Scientific projects

MeDi-Pro 1st survey () No.
of responses = 33

Legend

Question text

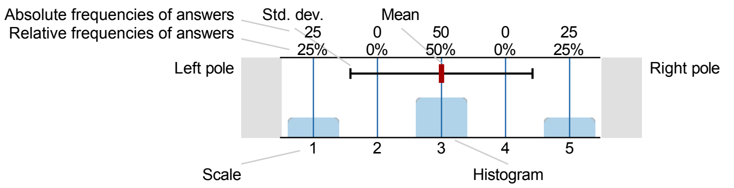

n=No. of responses
av.=Mean
dev.=hour dev.
ab.=abstention

Personal questions

Gender

n=32

Female	56.3%
Male	43.8%
Not specified	0%

Age

n=32

18-30 years	15.6%
31-40 years	18.8%
41-50 years	31.3%
51-60 years	34.4%
≥ 61 years	0%

Profession (in which you mainly work)

n=31

Caregiver	32.3%
Doctor	29%
Quality management/ risk management	22.6%
Management	6.5%
Other:	9.7%

Other:

■ Pharmacist

■ Nursing scientist■

Scientist

Professional experience

n=32

0-2 years	3.1%
3-6 years	12.5%
7-10 years	9.4%
11-20 years	25%
>20 years	50%

Code for recognition in the 2nd Delphi survey Year of birth mother e.g. 1955

- 1922
- 1929 (2 Counts)
- 1932
- 1937
- 1938 (2 Counts)
- 1941 (2 Counts)
- 1942
- 1944 (2 Counts)
- 1947
- 1948
- 1950
- 1951
- 1952
- 1953
- 1954 (2 Counts)
- 1955 (2 Counts)
- 1956
- 1959
- 1963 (2 Counts)
- 1965
- 1966
- 1967
- 1968 (2 Counts)
- 1969
- 1985

1st letter First name Mother■

A (3 Counts)

■ B

■ C (2 counts)

■ D

■ E (2 counts)■

G

■ H (4 Counts)

■ I (3 counts)■

M

■ R (5 counts)

■ S (2 counts)■

T

■ U

■ W (2 counts)

■ b

■ c (2 counts)

■ i

1st letter First name Father■

A (4 Counts)

■ E

■ F (5 counts)

■ G (2 Counts)

■ H (2 Counts)

■ J (4 counts)■

K

■ M (2 Counts)

■ R (5 counts)

■ S

■ W (2 Counts)

■ f (2 counts)■

g

■ r

General information

We ask you to evaluate the risk clusters as best as possible according to probability of occurrence and impact. The risk clusters consist of individual risks that are examples of a group of individual risks.
A scale of 1-10 is available for rating the risk clusters, with 10 representing the highest probability of occurrence and fatal impact and 1 representing no to very low probability of occurrence and low impact for patients. When rating, always take into account the risk that you consider to be the greatest risk for the respective risk cluster, regardless of how you rate the other individual risks in this cluster.

For each question, you also have the opportunity to comment on the risk cluster. This would be, for example, the absence of further risks in this risk cluster. The aim of the survey is to

identify the most relevant risks in the medication process.

Chapter: Recording

Risk cluster: Inadequate communication about prescribed medication between the private practice and the hospital.

How do you estimate the probability of occurrence of this risk cluster in the hospital? Please mark with a cross:

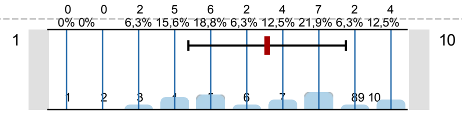

n=32
av.=6.6
dev.=2.2

How do you assess the impact of this risk cluster for patients in hospital? Please mark with a cross:

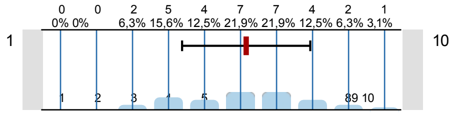

n=32
av.=6.2
dev.=1.8

Do you have any

comments? ■ .

■ ELGA brings improvement, many patients and relatives can provide information about actual medication

■ Healthcare systems in different countries are different - also depending on the respective documentation systems that are available across departments. -Am I making an assessment based on my hospital? what kind of patient am I assuming? an informed patient? or a patient who needs help with medication management?

■ The patient usually has a media list or the medication boxes with them. Unfortunately, there are no dosages in ELGA.

■ Patients sometimes forget to specify medications during the admission process to the hospital, e.g. medication on demand, sleep medication or NMH in the case of paused OAC, if these have not been documented in writing.

- Insufficient regulation of eMedication (mandatory content?) means that information cannot be used. eMedication process via eCard
- currently inadequate. Discontinued medication often not recognizable.
- cannot be assessed

Risk cluster: Incomplete medication list on admission with discrepancies in the medication history (e.g. different lists from patient, GP, specialist, electronic medication recording).

How do you estimate the probability of occurrence of this risk cluster in the hospital? Please mark with a cross:

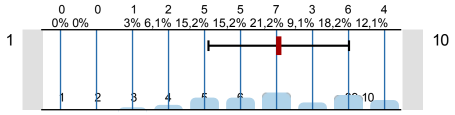

n=33
av.=7.1
dev.=2

How do you assess the impact of this risk cluster for patients in hospital? Please mark with a cross:

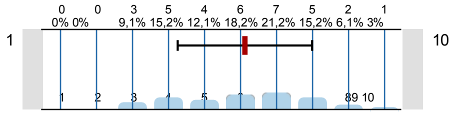

n=33
av.=6.1
dev.=1.9

Do you have any

comments? ■ .

- Lack of documentation, e.g. if patients change the dosage of medication or stop taking it themselves.
- High for patients who are unresponsive and incapacitated. Medium for others, as this should actually be resolved during the medical consultation.
- Is very dependent on the family doctor, the patient's family, etc. - Preparation for admission to hospital also plays an important role - what documents etc. does the patient receive for admission?
- See above
- No standardized digital medical record that is maintained by all specific professional groups

Risk cluster: Patient-related predictors on admission (e.g. lack of compliance, health status, type of admission (acute, elective) or health literacy).

How do you estimate the probability of occurrence of this risk cluster in the hospital? Please mark with a cross:

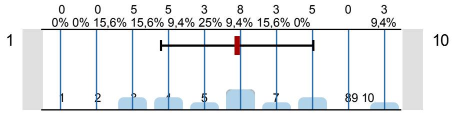

n=32
av.=5.9
dev.=2.1

How do you assess the impact of this risk cluster for patients in hospital? Please mark with a cross:

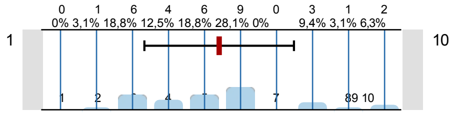

n=32
av.=5.4
dev.=2.1

Do you have any

comments? ■ .

- Risk relevant in combination with the two previous clusters, otherwise clinical professionalism and custodial care should minimize this risk.
- cannot be assessed
- very department-specific - areas with a high number of elderly and very elderly patients have a higher risk

Risk cluster: Low professional experience of the admitting doctor or lack of support from experienced doctors in the hospital.

How do you estimate the probability of occurrence of this risk cluster in the hospital? Please mark with a cross:

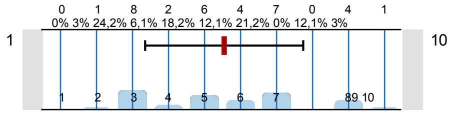

n=33
av.=5.5
dev.=2.2

How do you assess the impact of this risk cluster for patients in hospital? Please mark with a cross:

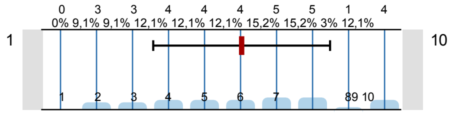

n=33
av.=6
dev.=2.5

Do you have any

comments? ■ .

■ From my point of view, the main problem is that on admission (whs. depending on the area, e.g. intensive care) all the patient's own medication is stopped. Which often makes sense - the problem later on in the ward, however, is that the treating physicians are not interested in streamlining the medication list for the patient and looking with him at how compliance could be improved (e.g. all medication in the morning, then it's done for the day).

■ In addition to professional experience, this also depends on the personality structure and learning behavior of the specialist? Knowing when and how support can be obtained.

■ Depends heavily on the self-reflection ability of the patient, or on the hierarchy and availability of experienced doctors. ■ If medication

prescription is NOT checked and questioned by an experienced doctor and/or nurse - high risk

Risk cluster: challenge with medications (polypharmacy (defined as more than 5 medications), generics vs. originator, high-risk medications, drug interactions).

How do you estimate the probability of occurrence of this risk cluster in the hospital? Please mark with a cross:

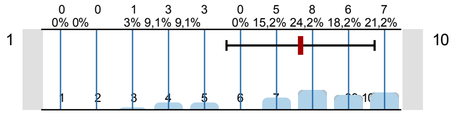

n=33
av.=7.7
dev.=2.1

How do you assess the impact of this risk cluster for patients in hospital? Please mark with a cross:

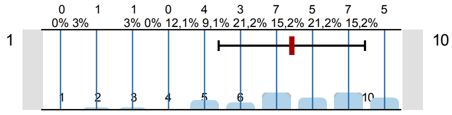

n=33
av.=7.4
dev.=2

Do you have any

comments? ■ .

■ Awareness of this issue has increased, patients in my area generally take a lot of medication■ Polypragmasia (as a sign of

helplessness) is one of THE problems in at-risk populations (geriatrics, psychiatry, etc.).

■ In my opinion, the risk here is not due to polypharmacy or the medication itself, but rather the refusal/non-use of medication catalogs and interaction tests/interchangeable preparation lists by the users.

Risk cluster: Allergy errors - allergies are not recorded, not or incorrectly documented or not taken into account, no indication of an allergy by patients.

How do you estimate the probability of occurrence of this risk cluster in the hospital? Please mark with a cross:

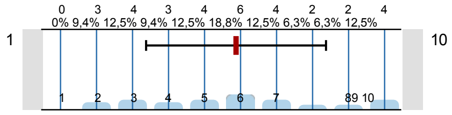

n=32
av.=5.9
dev.=2.5

How do you assess the impact of this risk cluster for patients in hospital? Please mark with a cross:

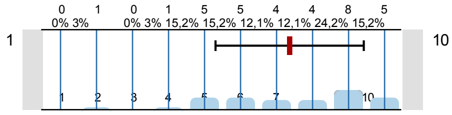

n=33
av.=7.4
dev.=2.1

Do you have any

comments? ■ .

■ There is also the reverse problem: an imprecise definition of what an allergy is. Patients may not receive treatments (penicillin, aspirin, etc.) due to suspected allergies that are dragged along with the diagnoses.

■ Depends on whether the allergies are known, allergy pass is required for elective admission to hospital. ■ Is

recorded very precisely by doctors and nursing staff

Chapter: Regulation

Risk cluster: General errors in the prescription (e.g. wrong medication, wrong dose, incomplete prescription and other types of errors such as omission errors, transmission errors, duplication errors).

How do you estimate the probability of occurrence of this risk cluster in the hospital? Please mark with a cross:

1 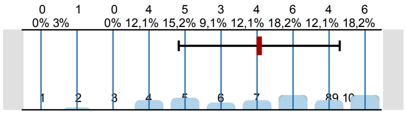

10

n=33
av.=7.1
dev.=2.2

How do you assess the impact of this risk cluster for patients in hospital? Please mark with a cross:

1 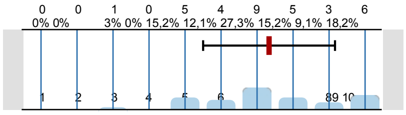

10

n=33
av.=7.3
dev.=1.8

Do you have any

comments? ■ .

■ Depending on the documentation, whether this is done electronically or on paper. We document electronically, with entered limits and, as a rule, medication prescriptions are agreed with senior physicians.

■ This is not uncommon with electronic documentation because the patient is not visited at the bedside but from the doctor's

office. ■ Significant improvement through electronic fever curve!

■ Caregivers point out incomplete prescriptions

■ Occurs almost daily, nursing checks very closely and points out prescription errors - time-consuming

■ you should get a hint here - comments on this question have become superfluous due to the risk cluster with questions 4.7 and 4.8.

Risk cluster: Unintentional/intentional skipping of an electronic warning during regulation, alarm fatigue due to repeated, too many or inappropriate warnings.

How do you estimate the probability of occurrence of this risk cluster in the hospital? Please mark with a cross:

1 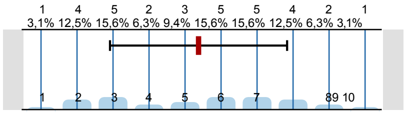

10

n=32
av.=5.4
dev.=2.5

How do you assess the impact of this risk cluster for patients in hospital? Please mark with a cross:

1 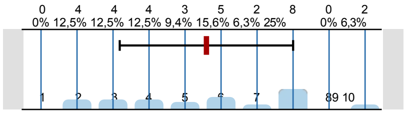

10

n=32
av.=5.6
dev.=2.4

Do you have any comments?

■ Depending on how the sensitivity is set - if it always alarms - the alarm will no longer be seen at some point. ■ The

warnings for prescriptions only make sense for individual particularly risky preparations.
As soon as several medications have to be prescribed or severe underlying diseases such as renal insufficiency or hepatic insufficiency are present, the value of such warning systems is currently low

■ Error messages via pop-ups are often associated with a technical defect in the program. (e.g. input incomplete, not connected to server, etc.) I therefore believe that it is a risk to design warnings about regulations and technical notifications in the same way. Ideally, different systems should be used (e.g. pop-ups for technical error messages and a warning screen with a checkbox for prescription warnings)

■ Currently no eFK, assumption■

cannot be assessed

Risk cluster: Difficulties with handwritten prescriptions (e.g. incomplete prescription, illegibility of prescription, prescription in pencil or "non-waterproof pen", use of correction varnish).

How do you estimate the probability of occurrence of this risk cluster in the hospital? Please mark with a cross:

1 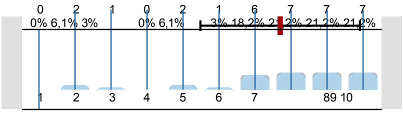 10

n=33
av.=7.7
dev.=2.2

How do you assess the impact of this risk cluster for patients in hospital? Please mark with a cross:

1 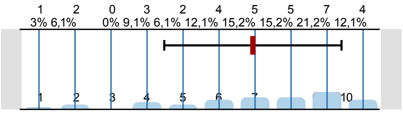 10

n=33
av.=6.9
dev.=2.5

Do you have any

comments? ■ .

■ Improvable through eFK

■ There are no handwritten prescriptions in our hospital, only electronic prescriptions are carried out

■ Sometimes ambiguities in handwritten orders can only be resolved by the person issuing the order, especially if the person carrying out the order is not familiar with handwriting.

■ Care asks if something cannot be read. Mistakes happen so rarely.

■ Occurs almost daily, nursing checks very carefully and points out prescription errors, illegibility, etc. - time-consuming

Risk cluster: Errors and challenges in the electronic regulation (e.g. lack of user-friendliness, errors in automated processes, incorrect use, incorrect/problematic default settings and features; additional need for paper documents; technology failure).

How do you estimate the probability of occurrence of this risk cluster in the hospital? Please mark with a cross:

1 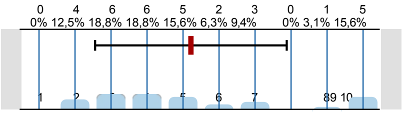 10

n=32
av.=5.2
dev.=2.7

How do you assess the impact of this risk cluster for patients in hospital? Please mark with a cross:

1 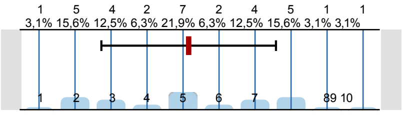 10

n=32
av.=5.2
dev.=2.4

Do you have any

comments? ■ .

■ No eFK yet, assumption

■ If the application system is already well established, otherwise the risk tends to be higher (8/8) in the implementation and refreezing phase.

■ cannot be assessed

Risk cluster: Challenges in prescribing complex medications/high-risk medications (e.g. polypharmacy, lack of control of drug interactions) due to lack of clinical-pharmacological knowledge (e.g. irrational, inappropriate and ineffective prescribing) and/or due to lack of prescribing schemes or non-use of existing prescribing schemes.

How do you estimate the probability of occurrence of this risk cluster in the hospital? Please mark with a cross:

1 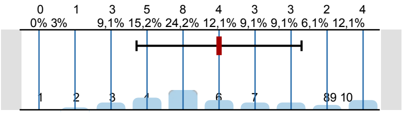 10

n=33
av.=6
dev.=2.3

How do you assess the impact of this risk cluster for patients in hospital? Please mark with a cross:

1 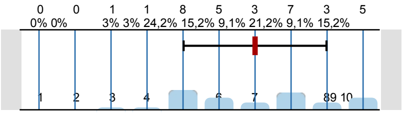 10

n=33
av.=7
dev.=2

Do you have any

comments? ■ .

■ A highly variable risk cluster (level of education, division of labor, departmental culture, etc.)

- Close cooperation with the pharmacy in our hospital
- I generally rate it highly - we work with a clinical pharmacologist precisely to avoid these problems, which certainly occur frequently Pharmacist on rounds several times a week would be helpful
-

Risk cluster: Wrong patient when prescribing (e.g. confusion of identity, same name of patients).

How do you estimate the probability of occurrence of this risk cluster in the hospital? Please mark with a cross:

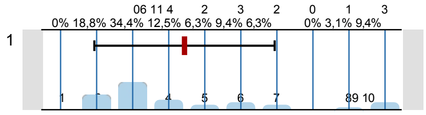

n=32
av.=4.4
dev.=2.5

How do you assess the impact of this risk cluster for patients in hospital? Please mark with a cross:

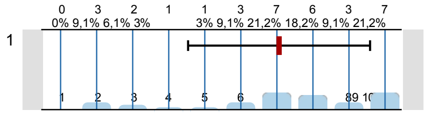

n=33
av.=7.1
dev.=2.5

Do you have any

comments? ■ .

■ Organized workflows have reduced risk

■ Medicines etc. are scanned in certain departments - in future this is to be comprehensive■ Identical

names do not occur often, this is always pointed out
Can occur - rather very rarely

Chapter: Review

Risk cluster: Lack of review/support for complex prescriptions by (clinical) pharmacists (e.g. high-risk medications, polypharmacy, complex indications and diagnoses).

How do you estimate the probability of occurrence of this risk cluster in the hospital? Please mark with a cross:

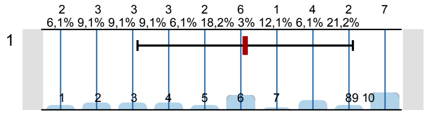

n=33
av.=6.1
dev.=3

How do you assess the impact of this risk cluster for patients in hospital? Please mark with a cross:

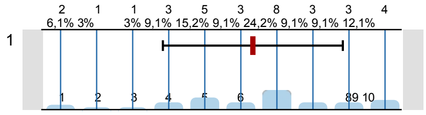

n=33
av.=6.3
dev.=2.5

Do you have any

comments? ■ .

■ The likelihood of this occurring naturally depends extremely on the structures and resources available locally and nationally. ■

Close cooperation, regular visits with the pharmacists in our hospital

■ International standard for a long time!

Chapter: Preparation/dispensing

Risk cluster: Errors in the preparation/dispensing of medication (e.g. errors in dividing tablets, incorrect medication, incorrect dose, incorrect calculation, missing or incorrect change to the ordered medication in the dispenser, missing or incorrect documentation, missing/incorrect/unclear labeling/labeling of prepared medication).

How do you estimate the probability of occurrence of this risk cluster in the hospital? Please mark with a cross:

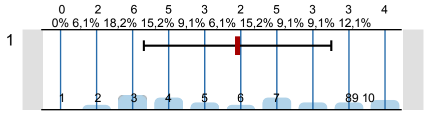

n=33
av.=5.9
dev.=2.6

How do you assess the impact of this risk cluster for patients in hospital? Please mark with a cross:

1 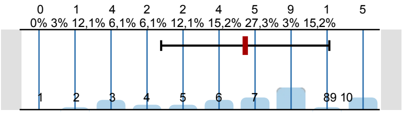

10

n=33
av.=6.7
dev.=2.3

Do you have any

comments? ■ .

■ 4- Eye principle, if possible (rarely feasible in most areas)- Control of dispensed medication■ Rapid change of replacement

medication can massively increase the risk

■ We often have similar dosages in our department - and a limited number of medications - specifically defined medication set

Risk cluster: mix-up of medications (e.g. errors with similar-looking medications, errors with similar-sounding medication names, mix-up of medication names/packaging).

How do you estimate the probability of occurrence of this risk cluster in the hospital? Please mark with a cross:

1 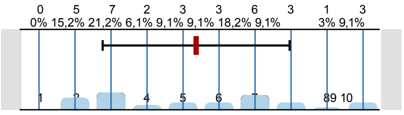

10

n=33
av.=5.4
dev.=2.6

How do you assess the impact of this risk cluster for patients in hospital? Please mark with a cross:

1 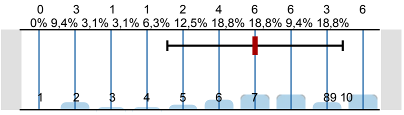

10

n=32
av.=7
dev.=2.4

Do you have any

comments? ■ .

■ 4-eyes principle, if possible (rarely feasible in most areas - personnel costs)- Checking of dispensed medication later by colleagues

■ 4-eyes principle is established

■ The increasingly frequent use of generic drugs forces us to control the drugs very well■ Prevention

through targeted labeling - and a defined set of drugs

Risk cluster: No application of guidelines and/or standards for safe preparation/dispensing (e.g. 4-eyes principle or readback method).

How do you estimate the probability of occurrence of this risk cluster in the hospital? Please mark with a cross:

1 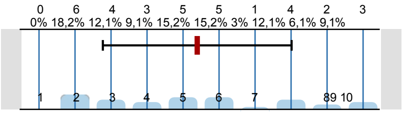

10

n=33
av.=5.4
dev.=2.6

How do you assess the impact of this risk cluster for patients in hospital? Please mark with a cross:

1 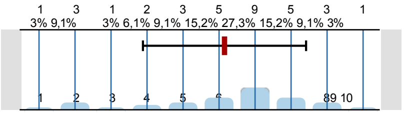

10

n=33
av.=6.2
dev.=2.3

Do you have any

comments? ■ .

■ 4- Eye principle, if possible (rarely feasible in most areas)- Checking of dispensed medication later by colleagues

■ As I consider the procedures mentioned to be relatively ineffective, I also estimate the impact of the lack of procedures to be relatively low. The omission of an ineffective preventive measure probably causes relatively little damage...

■ We have different standards but also compatibility lists etc.

Risk cluster: Wrong patient during preparation/dispensing of medication (e.g. preparation of medication for the wrong patient).

How do you estimate the probability of occurrence of this risk cluster in the hospital? Please mark with a cross:

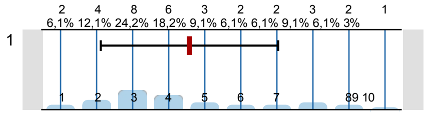

n=33
av.=4.6
dev.=2.5

How do you assess the impact of this risk cluster for patients in hospital? Please mark with a cross:

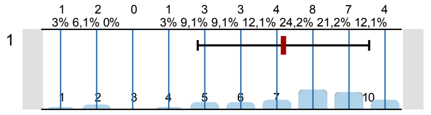

n=33
av.=7.2
dev.=2.4

Do you have any

comments? ■ .

■ 4-eyes principle is established

■ Nurses have one or two patients for whom they prepare and administer medication.

Risk cluster: Difficulties with the delivery and storage of medicines (e.g. late or incorrect deliveries, no information regarding originator vs. generics, no secure storage of dispensers/medicines).

How do you estimate the probability of occurrence of this risk cluster in the hospital? Please mark with a cross:

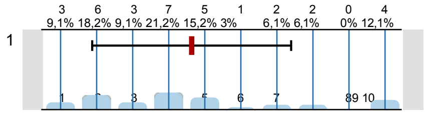

n=33
av.=4.6
dev.=2.8

How do you assess the impact of this risk cluster for patients in hospital? Please mark with a cross:

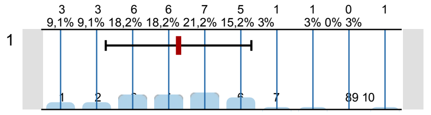

n=33
av.=4.3
dev.=2

Do you have any

comments? ■ .

■ Rapid change of substitute preparations can massively increase the risk

■ many generics, many pass-through articles, no daily orders from Apo ad companies etc...

Risk cluster: Lack of communication/misunderstandings in communication (e.g. errors in telephone instructions, misunderstandings regarding medication names, dosage, interval, dosage form, patient).

How do you estimate the probability of occurrence of this risk cluster in the hospital? Please mark with a cross:

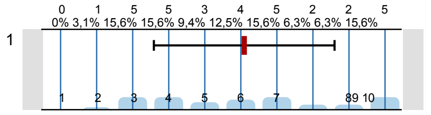

n=32
av.=6.1
dev.=2.5

How do you assess the impact of this risk cluster for patients in hospital? Please mark with a cross:

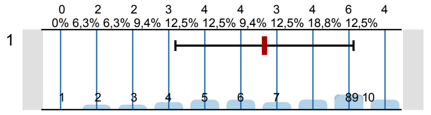

n=32
av.=6.7
dev.=2.5

Do you have any comments?

■ Depending on the experience of the caregiver, whether there is a demand because, for example, the dosage is too high or missing...

■ Examples from clinical practice: Suggammadex was confused with succinylcholine because "Suggi" was said. Esmeron was confused with esmolol because it was apparently misunderstood by the other person.

■ Fixed by eFK

Risk cluster: Errors in the preparation of intravenous medication (e.g. incorrect medication, incorrect diluent solution, incorrect labeling, incorrect dose, bacterial contamination, incompatibility or instability).

How do you estimate the probability of occurrence of this risk cluster in the hospital? Please mark with a cross:

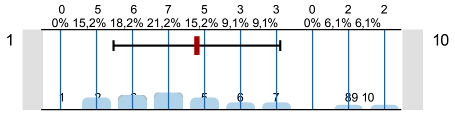

n=33
av.=4.8
dev.=2.3

How do you assess the impact of this risk cluster for patients in hospital? Please mark with a cross:

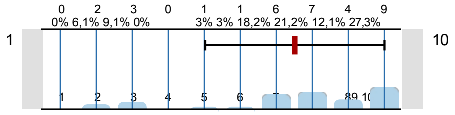

n=33
av.=7.5
dev.=2.5

Do you have any

comments? ■ .

■ Our department has compatibility lists in the documentation system, dilution specifications, etc.

■ In my area, a lot of IV medication, clear procedures, checking of prepared medication by another caregiver

Chapter: Administration

Risk cluster: medication administration errors (e.g. wrong medication, wrong dosage, wrong route of administration, confusion of similar looking or similar sounding medications, wrong time of administration, unauthorized medications, omission errors, incorrect verification activities, difficulties with infusion equipment, confusion of medication packaging, incorrect labeling of medication on packaging).

How do you estimate the probability of occurrence of this risk cluster in the hospital? Please mark with a cross:

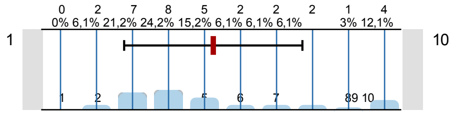

n=33
av.=5.2
dev.=2.5

How do you assess the impact of this risk cluster for patients in hospital? Please mark with a cross:

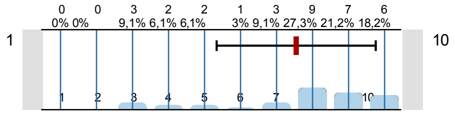

n=33
av.=7.5
dev.=2.2

Do you have any

comments? ■ .

■ Because of the ward, we administer most of the medication i/V - it certainly depends on which department we are talking about.

Risk cluster: Lack of documentation and communication about medication intake, lack of intake, interactions (e.g. allergies and drug interactions).

How do you estimate the probability of occurrence of this risk cluster in the hospital? Please mark with a cross:

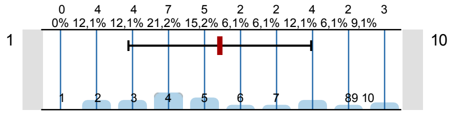

n=33
av.=5.4
dev.=2.5

How do you assess the impact of this risk cluster for patients in hospital? Please mark with a cross:

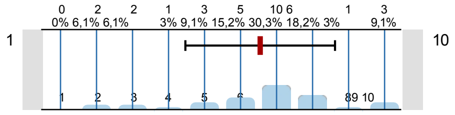

n=33
av.=6.5
dev.=2.1

Do you have any

comments? ■ .

■ Happens whs. more with handwritten documentation■

Too few qualified staff increases the risk

■ is documented very precisely through maintenance

Risk cluster: Incorrect patient identification during administration (e.g. incorrect patient identification, medication is administered to the wrong patient).

How do you estimate the probability of occurrence of this risk cluster in the hospital? Please mark with a cross:

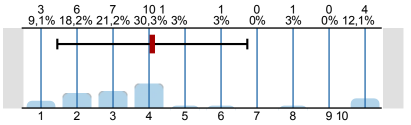

n=33
av.=4.1
dev.=2.6

How do you assess the impact of this risk cluster for patients in hospital? Please mark with a cross:

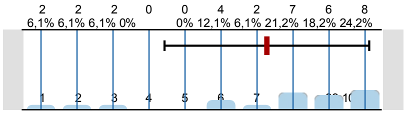

n=33
av.=7.3
dev.=2.8

Do you have any comments?

■ Effect depends on the type of medication

■ The standard practice of looking at the identification bracelets is perceived by patients as extremely unprofessional. ■ Nursing

staff look after 1-2 patients here - certainly depending on the number of patients to be cared for and the Qualification of the specialist.

■ Care groups reduce the risk

Risk cluster: Administration of medication to patients with cognitive impairment or lack of compliance (e.g. e.g. missing or incorrect control and monitoring of medication intake, missing or incorrect documentation of administration).

How do you estimate the probability of occurrence of this risk cluster in the hospital? Please mark with a cross:

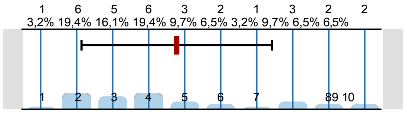

n=31
av.=4.8
dev.=2.6

How do you assess the impact of this risk cluster for patients in hospital? Please mark with a cross:

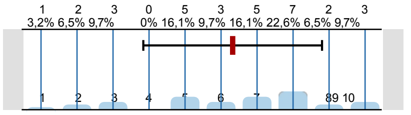

n=31
av.=6.3
dev.=2.5

Do you have any

comments? ■ .

■ These patients have a controlled drug intake■ no details

possible

■ Controlled medication intake as soon as it is noticed that the patient is not taking medication

Risk cluster: Problems with infusion pumps (e.g. incorrect handling, incorrect setting, uncalibrated/uncalibrated pumps, different pump characteristics, infusomats without locking, lack of flushing, lack of uniform standards).

How do you estimate the probability of occurrence of this risk cluster in the hospital? Please mark with a cross:

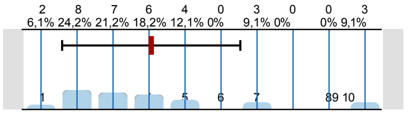

n=33
av.=4.1
dev.=2.5

How do you assess the impact of this risk cluster for patients in hospital? Please mark with a cross:

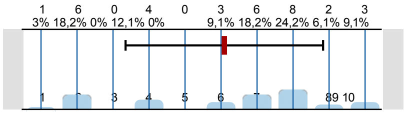

n=33
av.=6.1
dev.=2.7

Do you have any

comments? ■ .

■ Working with technical aids is routine for us

■ In principle, the pumps should be configured in such a way that hardly any errors are possible - our experience is

positive■ Too few qualified personnel increases the risk

■ All employees are trained, there is always a second employee on duty who can operate pumps uniform pumps (one brand)

Risk cluster: Errors in connection with barcode technology (e.g. missing/incorrect barcodes, systems not communicating with each other).

How do you estimate the probability of occurrence of this risk cluster in the hospital? Please mark with a cross:

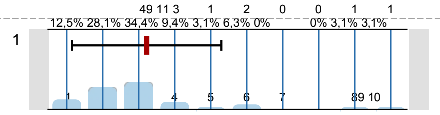

n=32
av.=3.2
dev.=2.1

How do you assess the impact of this risk cluster for patients in hospital? Please mark with a cross:

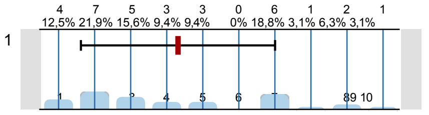

n=32
av.=4.3
dev.=2.7

Do you have any

comments? ■ .

■ Systems are normally synchronized in advance

■ We don't work with it - I would have liked to have ticked "not

applicable"■ not assessable for me

Chapter: Monitoring

Risk cluster: Incorrect further prescription of medication (e.g. without checking reactions/interactions, missing or incorrect prescription or continued prescription, missing or incorrect labeling, missing further prescription before weekends or public holidays, further prescription of medication was not stopped).

How do you estimate the probability of occurrence of this risk cluster in the hospital? Please mark with a cross:

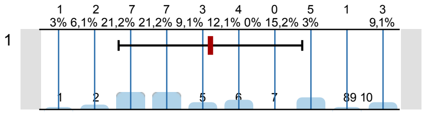

n=33
av.=5.2
dev.=2.5

How do you assess the impact of this risk cluster for patients in hospital? Please mark with a cross:

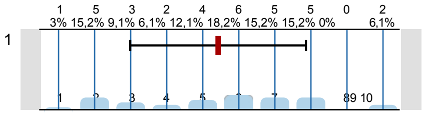

n=33
av.=5.4
dev.=2.4

Do you have any

comments? ■ .

■ Improved by eFK

■ Especially on the day of surgery, medication is not clearly paused or not correctly reintroduced

afterwards. ■ Is monitored very closely by nursing staff, as this happens very often - very time-

consuming

Risk cluster: Poor communication between employees (e.g. between nursing staff or doctors regarding medication such as effects, side effects, interactions, orders, changes and the passing on of incorrect information).

How do you estimate the probability of occurrence of this risk cluster in the hospital? Please mark with a cross:

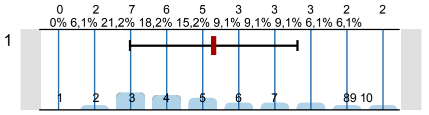

n=33
av.=5.3
dev.=2.3

How do you assess the impact of this risk cluster for patients in hospital? Please mark with a cross:

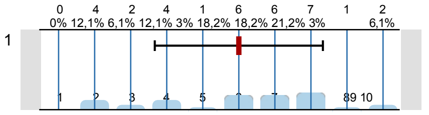

n=33
av.=6
dev.=2.3

Do you have any

comments? ■ .

■ Improved by eFK

■ Person-dependent, mostly good communication

Chapter: Dismissal

Risk cluster: Lack of communication/information between hospital and community-based doctors, nursing services and other healthcare providers regarding medication requirements on discharge; electronic communication, interface problems.

How do you estimate the probability of occurrence of this risk cluster in the hospital? Please mark with a cross:

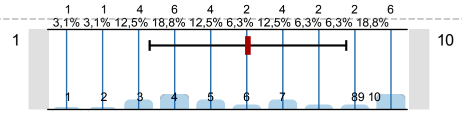

n=32
av.=6
dev.=2.7

How do you assess the impact of this risk cluster for patients in hospital? Please mark with a cross:

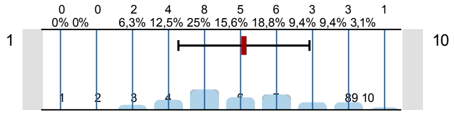

n=32
av.=6.1
dev.=1.8

Do you have any

comments? ■ .

■ Same problem as at the beginning with e-medication (only now in the other direction); partly unreflected transfer of the hospital medication into the doctor's letter (?);

■ Every patient receives a doctor's letter with medication, in the case of transfers or home nursing also a nursing/transfer report■ no

information possible

Risk cluster: Lack of communication/information with patients and relatives (e.g. need for medication, medication not explained).

How do you estimate the probability of occurrence of this risk cluster in the hospital? Please mark with a cross:

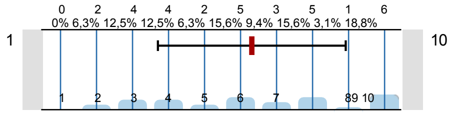

n=32
av.=6.3
dev.=2.6

How do you assess the impact of this risk cluster for patients in hospital? Please mark with a cross:

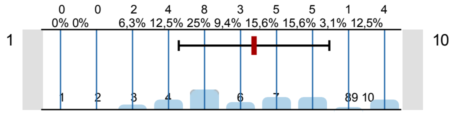

n=32
av.=6.4
dev.=2.1

Do you have any comments?

■ In my opinion, the probability of occurrence is so high mainly because many patients cannot remember the medication information, and not because no information was provided.

■ Very different, implemented across the board in my area (patient is trained, informed and has medication for at least 2 days or prescriptions on discharge)

■ no specification possible

Risk cluster: Lack of availability of medication in private practice after an inpatient stay (e.g. original preparations vs. generics).

How do you estimate the probability of occurrence of this risk cluster in the hospital? Please mark with a cross:

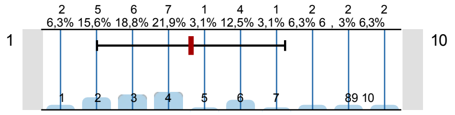

n=32
av.=4.6
dev.=2.6

How do you assess the impact of this risk cluster for patients in hospital? Please mark with a cross:

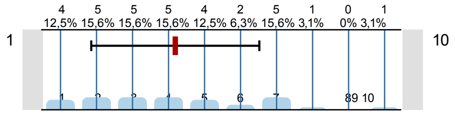

n=32
av.=4.2
dev.=2.3

Do you have any

comments? ■ .

■ Generics are not so much the problem, but the lack of approval by the chief medical officer (this used to go through more quickly than it does now electronically...??)

- Prescription guidelines must of course be observed! no information
- possible
- very different, in my area patients receive new medication to take home for 2 days or receive a prescription (less often)

Risk cluster: Inadequate/incorrect written discharge information ("doctor's letter") (e.g. missing/incomplete prescription of medication, recommended medication is incomplete, discharge letter does not reach patient, GP, specialist, nursing home or reaches them late).

How do you estimate the probability of occurrence of this risk cluster in the hospital? Please mark with a cross:

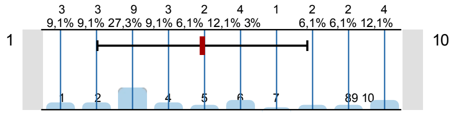

n=33
av.=4.9
dev.=2.9

How do you assess the impact of this risk cluster for patients in hospital? Please mark with a cross:

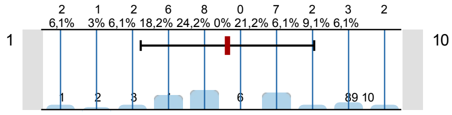

n=33
av.=5.6
dev.=2.4

Do you have any

comments? ■ .

- Discharges with a doctor's letter are already standard
- In my area, discharge only with a doctor's letter and agreed outpatient appointments
- see 6.3

Risk cluster: Lack of discharge assessment with regard to patients' further (nursing) needs (e.g. patients are unable to organize medication themselves, have problems paying for medication, live far away from GPs/pharmacies in a remote area).

How do you estimate the probability of occurrence of this risk cluster in the hospital? Please mark with a cross:

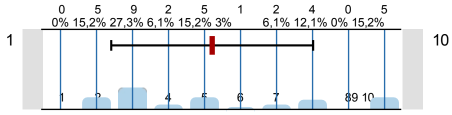

n=33
av.=5.2
dev.=2.8

How do you assess the impact of this risk cluster for patients in hospital? Please mark with a cross:

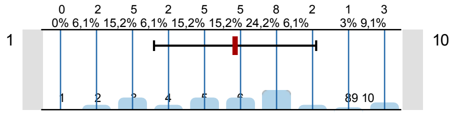

n=33
av.=5.8
dev.=2.3

Do you have any comments?

- Is currently not requested as standard and is also not written in the doctor's letter as standard.
- Lack of time
- very different, in my area the nursing discharge management is very well implemented, social worker is established, discharge consultations on a wide range of topics are established, relatives are always involved

Chapter: Healthcare personnel (competence)

Risk cluster: Problematic environment during the individual steps of the medication process (e.g. noise, poor lighting, emergencies, chaotic working environment, interruption/distraction and high workload of staff, e.g. due to understaffing, poor ward equipment).

How do you estimate the probability of occurrence of this risk cluster in the hospital? Please mark with a cross:

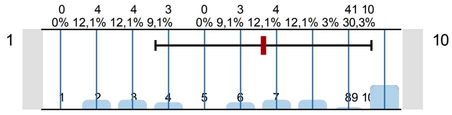

n=33
av.=6.6
dev.=3

How do you assess the impact of this risk cluster for patients in hospital? Please mark with a cross:

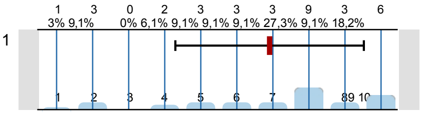

n=33
av.=6.9
dev.=2.6

Do you have any

comments? ■ .

■ certainly applies to many areas

Risk cluster: Knowledge-based errors and lack of training/experience (e.g. lack of knowledge, lack of qualified staff, working with inexperienced or new staff, frequent staff turnover and lack of training/education of people involved in the medication process).

How do you estimate the probability of occurrence of to this risk cluster in the hospital? Please mark with a cross:

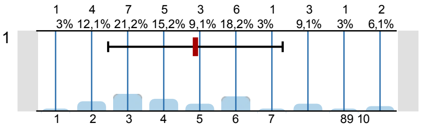

n=33
av.=4.9
dev.=2.4

How do you assess the impact of this risk cluster for patients in hospital? Please mark with a cross:

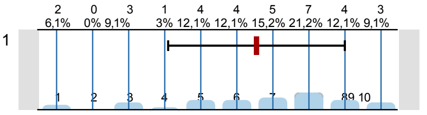

n=33
av.=6.5
dev.=2.5

Do you have any

comments? ■ .

Risk cluster: Lack of compliance with specifications/guidelines regarding the medication process of employees and lack of guidelines and standards in the hospital regarding the medication process.

How do you estimate the probability of occurrence of to this risk cluster in the hospital? Please mark with a cross:

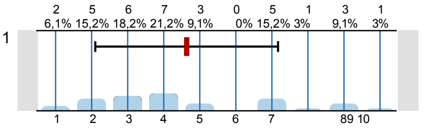

n=33
av.=4.6
dev.=2.5

How do you assess the impact of this risk cluster for patients in hospital? Please mark with a cross:

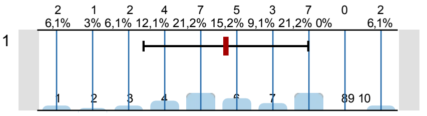

n=33
av.=5.7
dev.=2.3

Do you have any

comments? ■ .

■ 4-eyes principle and read-back method cannot be implemented - high workload and insufficient nursing staff

Risk cluster: Poor employee health (e.g. fatigue, physical exhaustion, stress) and problematic personalities (e.g. lack of error awareness and conscientiousness, complacency)

How do you estimate the probability of occurrence of this risk cluster in the hospital? Please mark with a cross:

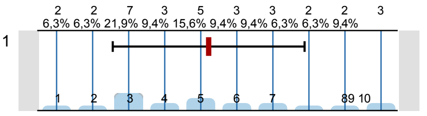

n=32
av.=5.2
dev.=2.7

How do you assess the impact of this risk cluster for patients in hospital? Please mark with a cross:

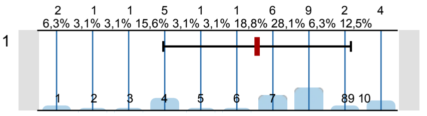

n=32
av.=6.6
dev.=2.6

Do you have any

comments? ■ .

■ no specification possible

Risk cluster: General communication difficulties between healthcare staff throughout the medication process, deficiencies in the communication structure/flow of information, lack of "speaking up" culture (expressing e.g. safety deficiencies to other staff), poor local working culture and lack of supervision/guidance from senior colleagues.

How do you estimate the probability of occurrence of this risk cluster in the hospital? Please mark with a cross:

1 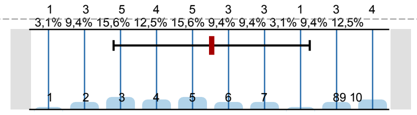 10

n=32
av.=5.5
dev.=2.7

How do you assess the impact of this risk cluster for patients in hospital? Please mark with a cross:

1 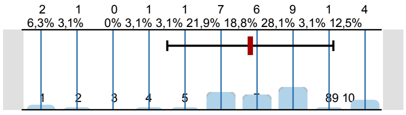 10

n=32
av.=6.8
dev.=2.3

Do you have any comments?

■ This risk cluster is highly dependent on the team and the climate of the respective station.■ no

information possible

■ very different how error culture is lived - depending on the person but generally improvement

Chapter: Patients and relatives

Risk cluster: Compliance of family caregivers/relatives with regard to medication (e.g. family caregivers do not understand the information on medication; family caregivers bring additional medication without consultation; family caregivers administer medication without consultation).

How do you estimate the probability of occurrence of this risk cluster in the hospital? Please mark with a cross:

1 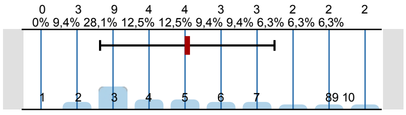 10

n=32
av.=5.1
dev.=2.4

How do you assess the impact of this risk cluster for patients in hospital? Please mark with a cross:

1 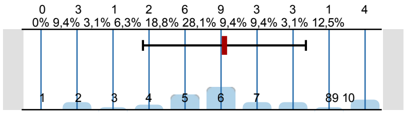 10

n=32
av.=6.1
dev.=2.3

Do you have any

comments? ■ .

■ no information possible

■ very rarely the case

Risk cluster: Risk factors relating to patients (e.g. lack of compliance, lack of health literacy, lack of knowledge about their own medication, incorrect use of medication).

How do you estimate the probability of occurrence of this risk cluster in the hospital? Please mark with a cross:

1 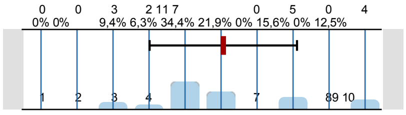 10

n=32
av.=6.1
dev.=2

How do you assess the impact of this risk cluster for patients in hospital? Please mark with a cross:

1 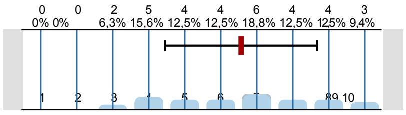 10

n=32
av.=6.6
dev.=2.1

Do you have any

comments? ■ .

■ no specification possible

Chapter: Digital process and IT security

Risk cluster: Missing/deficient hardware (e.g. lack of equipment for using electronic systems, outdated devices, poor WLAN technology).

How do you estimate the probability of occurrence of this risk cluster in the hospital? Please mark with a cross:

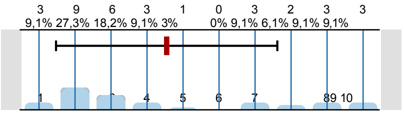

n=33
av.=4.5
dev.=3.1

How do you assess the impact of this risk cluster for patients in hospital? Please mark with a cross:

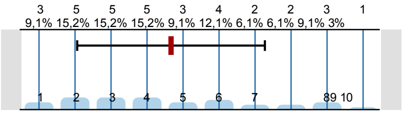

n=33
av.=4.7
dev.=2.6

Do you have any

comments? ■ .

■ Just because it is slower/more cumbersome does not mean that the result is worse - errors are less clearly hardware-related, usually a UX problem or user problem, because the inadequate hardware leads to careless work (workarounds, shortcuts...).

■ No secure process without technical equipment! ■ eFK

performance much too slow!!!!

Risk clusters: Poor software (including all health information technology in the hospital), missing/poorly designed interfaces between programs used in the medication process (e.g. for admission, prescription, ordering, inventory, discharge), poor user-friendliness, unforeseen interaction of systems, software errors, lack of security.

How do you estimate the probability of occurrence of this risk cluster in the hospital? Please mark with a cross:

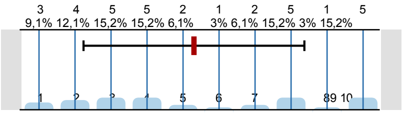

n=33
av.=5.3
dev.=3.1

How do you assess the impact of this risk cluster for patients in hospital? Please mark with a cross:

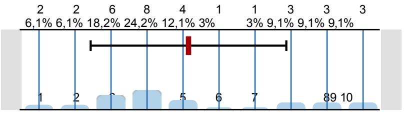

n=33
av.=5.2
dev.=2.7

Do you have any

comments? ■ .

■ The interface between the pharmacy (purchasing) and the reconciliation with the medication database urgently needs to be reformed. The 'maintenance' of the medication database also needs to be professionalized and standardized!!!!!'

Risk cluster: Deficiencies in the implementation of health information technologies (e.g. poor implementation plans, training and strengthening of IT skills of health personnel have not taken place).

How do you estimate the probability of occurrence of this risk cluster in the hospital? Please mark with a cross:

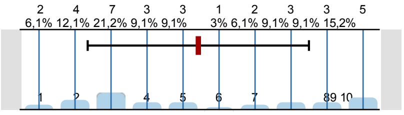

n=33
av.=5.4
dev.=3.1

How do you assess the impact of this risk cluster for patients in hospital? Please mark with a cross:

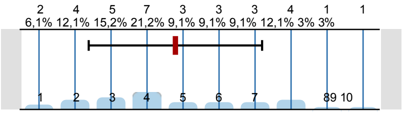

n=33
av.=4.8
dev.=2.4

Do you have any comments?

■ Especially non-tech-savvy colleagues have difficulties with digital devices and/or take longer to use them. (e.g. writing with a keyboard)

General comments

Is there anything else you would like to tell us? Are you missing a risk cluster?■ .

■ To a large extent, the questionnaire lists causes for risks in medication and not the risks themselves. The evaluation of the probability of occurrence and in particular the effects, which is used to assess risks, is unsuitable for causes! The mixing of causes and risks under the term cluster makes a meaningful assessment virtually impossible - please reconsider the method!

■ Interclinical interfaces

■ No, I think the presentation of the clusters is quite complete from my point of

view. ■ Very, very important project!!!

Thank you for your cooperation!
