# Supplementary material for: Risks in the analogue and digitally-supported medication process and potential solutions to increase patient safety in the hospital: A mixed methods study
Source: PLoS One. 2024 Feb 27;19(2):e0297491. doi: 10.1371/journal.pone.0297491 (PMC10898776; doi:10.1371/journal.pone.0297491)
Supplement: S4 File — Survey (English). (PDF) [file pone.0297491.s005.pdf]

Scientific projects

MeDi-Pro 2nd survey () No.
of responses = 28

Legend

Question text

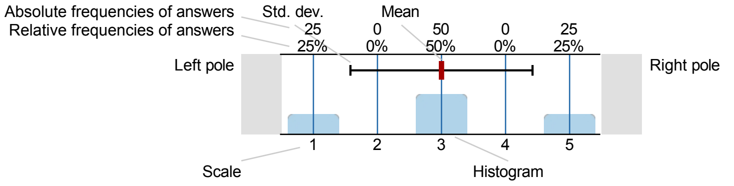

n=No. of responses
av.=Mean
dev.=hour dev.
ab.=abstention

Part 1: Questions about the person

Gender

n=28	
Female	64.3%
Male	35.7%
Miscellaneous	0%
Not specified	0%

Age

n=28	
18-30 years	17.9%
31-40 years	21.4%
41-50 years	25%
51-60 years	32.1%
≥ 61 years	3.6%

Profession (in which you mainly work)

n=28	
Caregiver	32.1%
Doctor	28.6%
Quality management/risk management	25%
Management	7.1%
Other:	7.1%

Professional experience

n=28	
0-2 years	7.1%
3-6 years	10.7%
7-10 years	14.3%
11-20 years	25%
>20 years	42.9%

Code for recognition in the 2nd Delpi survey Year of birth

mother e.g. 1955

- 1920
- 1922
- 1930

■ 1932

■ 1936

■ 1938

■ 1939

■ 1940

■ 1941 (2 Counts)

■ 1944 (2 Counts)

■ 1948

■ 1951

■ 1952

■ 1953

■ 1954

■ 1955

■ 1956

■ 1959

■ 1963 (2 Counts)

■ 1965

■ 1966

■ 1967

■ 1969

1st letter First name Mother■

A

■ B (2 Counts)

■ C (2 counts)

■ E (2 counts)■

G

■ H (3 Counts)

■ I (3 Counts)

■ R (4 counts)

■ S (2 Counts)

■ W (2 counts)■

a

■ c

■ h (2 counts)■

m

1st letter First name Father■

A (4 Counts)

■ F (4 Counts)

- G (3 Counts)
- H (2 Counts)
- J (2 counts)
- K
- R (5 counts)
- S
- a
- e
- g
- k
- w

General information

We ask you to rate the top 15 risk clusters in terms of the potential of solutions for strengthening employee skills and the potential of solutions in the area of digitalization. To evaluate the solution potential of the risk clusters, you can again use a scale of 1-10, where 1 stands for a very low solution potential and 10 for a very high solution potential.

For each risk cluster, you also have the option of submitting a specific solution proposal in the form of a comment.

The aim of the survey is to identify possible solutions for risks in the medication process that could be used in practice in the future.

Top risks - Inclusion

Admission: Inadequate communication about prescribed medication between the private practice and the hospital.

How high do you estimate the potential of a solution by strengthening employee competence (e.g. training and further education, etc.) for this risk cluster in the hospital?

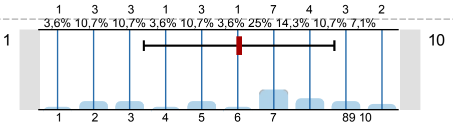

n=28
av.=6
dev.=2.6

How high do you estimate the potential of a solution in the area of digitalization (e.g. new software and hardware solutions, etc.) for this risk cluster in the hospital?

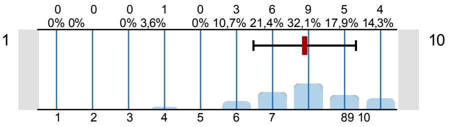

n=28
av.=7.9
dev.=1.4

Do you have a specific solution proposal for one of the risks mentioned in this risk cluster?

- Current digitalization poorly implemented and not very practicable (ELGA); new development necessary■

Entry of long-term medication in the ELGA by the GP

- Electronic "list" of prescribed medication
- Also enter dosages in ELGA, mandatory entry of all medications in ELGA by prescribing physicians
- Sensitization of employees to communication with external areas; additional facilitation of the identification of possible risk factors and prescribed medication through a digital tool
- Strengthening the GP-hospital network, ELGA is a first step

Admission: Incomplete medication list on admission with discrepancies in the medication history (e.g. different lists from patient, GP, specialist, electronic medication recording).

How high do you estimate the potential of a solution by strengthening employee competence (e.g. training and further education, etc.) for this risk cluster in the hospital?

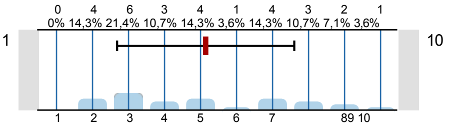

n=28
av.=5.1
dev.=2.5

How high do you estimate the potential of a solution in the area of digitalization (e.g. new software and hardware solutions, etc.) for this risk cluster in the hospital?

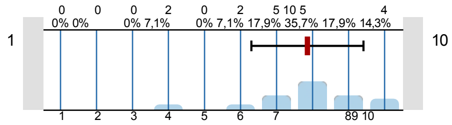

n=28
av.=7.9
dev.=1.6

Do you have a specific solution proposal for one of the risks mentioned in this risk cluster?

■ eFK will probably result in improvements, tws solution approaches already exist, current digitization poorly implemented and not very practicable (ELGA); new development necessary

■ ELGA? Mandatory for such data?

■ Electronic "list" of prescribed medication with expiry date

■ Medication plan with QR code for scanning into hospital information system■

Transfer of hospital discharge medication to ELGA

Admission: Challenges with medications (polypharmacy (defined as more than 5 medications), generics vs. originator, high-risk medications, drug interactions).

How high do you estimate the potential of a solution by strengthening employee competence (e.g. training and further education, etc.) for this risk cluster in the hospital?

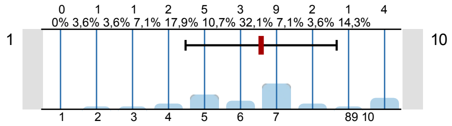

n=28
av.=6.6
dev.=2.1

How high do you estimate the potential of a solution in the area of digitalization (e.g. new software and hardware solutions, etc.) for this risk cluster in the hospital?

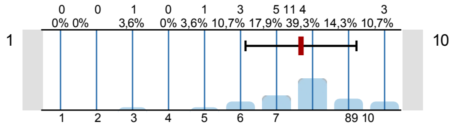

n=28
av.=7.7
dev.=1.5

Do you have a specific solution proposal for one of the risks mentioned in this risk cluster?

■ Automatic checking of interactions with "intelligent" algorithms, only warning of interactions not sufficient

■ Every patient with more than 5 medications could receive an evaluation of their medication■

Pharmacist on site, who provides advice and knows which medications are currently available■

Software solution - which runs in the background to the electronic medication list!

■ Automated presentation of potential risks (double prescription, incorrect dosage, etc.)■

Digital warning systems, increased medication training for doctors,

Admission: Allergy errors - allergies are not recorded, not or incorrectly documented or not taken into account, no indication of an allergy by patients.

How high do you estimate the potential of a solution by strengthening employee competence (e.g. training and further education, etc.) for this risk cluster in the hospital?

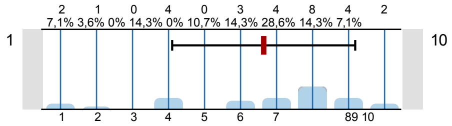

n=28
av.=6.6
dev.=2.5

How high do you estimate the potential of a solution in the area of digitalization (e.g. new software and hardware solutions, etc.) for this risk cluster in the hospital?

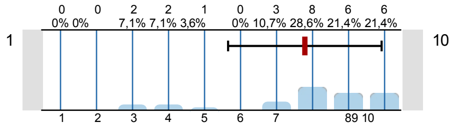

n=28
av.=7.8
dev.=2.1

Do you have a specific solution proposal for one of the risks mentioned in this risk cluster? ■ Allergies are

entered into the ELGA by the family doctor

■ Electronic "list" of prescribed medication and allergies

■ If letters can only be released if an entry or note "no allergy" has been made in the Allergies field■ for eMedication:

Prescription of medication for allergies - system does not simply allow this - clearly visible warning

■ Digital queries and warning systems, increased medication training for staff

■ Central data collection e.g. by general practitioners and central adaptation of data

Top risks - Regulation

Prescription: General errors in the prescription (e.g. wrong medication, wrong dose, incomplete prescription and other types of errors such as omission errors, transcription errors, duplication errors).

How high do you estimate the potential of a solution by strengthening employee competence (e.g. training and further education, etc.) for this risk cluster in the hospital?

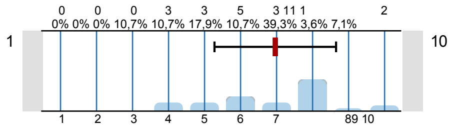

n=28
av.=7
dev.=1.7

How high do you estimate the potential of a solution in the area of digitalization (e.g. new software and hardware solutions, etc.) for this risk cluster in the hospital?

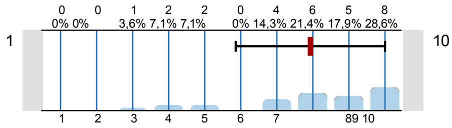

n=28
av.=7.9
dev.=2.1

Do you have a specific solution proposal for one of the risks mentioned in this risk cluster?

■ A good software for an e-fever curve and the possibility of entering medication in the outpatient doctor's letter via Medis would be helpful.
Unfortunately, the medication lists of other clinics cannot be copied and pasted, e.g. the medications for hematology are very clearly presented in the doctor's letters but cannot be copied and pasted into your own doctor's letter.

■ Medication must be clicked in the correct dose in the digital fever curve

■ Medication prescriptions from GPs should be checked by specialists or certain medications should only be prescribed by specialists, training of staff in the hospital only makes sense in part, as not all medications prescribed externally can be subsequently revised and changed for all patients in the hospital, this responsibility should be assumed by external doctors

■ e Medication: system indicates incomplete prescription, omissions, etc.

Prescription: difficulties with the handwritten prescription (e.g. incomplete prescription, illegibility of the prescription, prescription in pencil or "non-waterproof pen", use of correction varnish).

How high do you estimate the potential of a solution by strengthening employee competence (e.g. training and further education, etc.) for this risk cluster in the hospital?

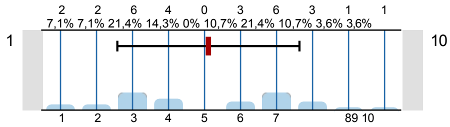

n=28
av.=5.1
dev.=2.5

How high do you estimate the potential of a solution in the area of digitalization (e.g. new software and hardware solutions, etc.) for this risk cluster in the hospital?

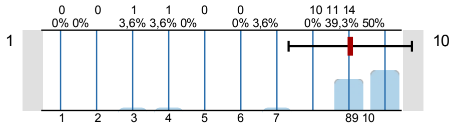

n=28
av.=9
dev.=1.7

Do you have a specific solution proposal for one of the risks mentioned in this risk cluster? ■

Implementation of the electronic temperature curve

■ Stockpile solution with eFK

■ digital fever curve solves this problem■

electronic signature

Prescribing: Challenges in prescribing complex medications/high-risk medications (e.g. polypharmacy, lack of control of drug interactions) due to lack of clinical pharmacological knowledge (e.g. irrational, inappropriate and ineffective prescribing) and/or lack of prescribing schemes or non-use of existing prescribing schemes.

How high do you estimate the potential of a solution in the area of digitalization (e.g. new software and hardware solutions, etc.) for this risk cluster in the hospital?

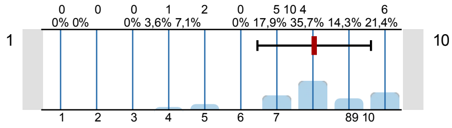

n=28
av.=8
dev.=1.6

How high do you estimate the potential of a solution by strengthening employee competence (e.g. training and further education, etc.) for this risk cluster in the hospital?

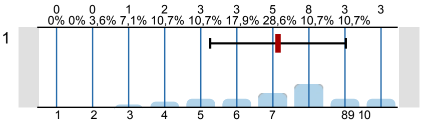

n=28
av.=7.1
dev.=1.9

Do you have a specific solution proposal for one of the risks mentioned in this risk cluster?

- 4.8 Pharmacist advises on polypharmacy - interactive conversation and discussion sometimes necessary
  4.7 eMedication: Prescription schemes are stored, system warns of incorrect prescriptions...
- Specialist information could be called up with one click in the digital temperature chart
- Not every contraindication or warning must be observed. It is often due to a lack of studies and safety considerations (financial security in the event of damage) on the part of the pharmaceutical companies that warnings are issued. If the warnings were only to be observed by means of a computer program, no children could be treated, as many frequently used drugs are off-level. Medical expertise can only be supplemented by a computer. The decision and responsibility still lies with the doctor.
- Software solution
- Promote holistic patient thinking when doctors prescribe medication (not just subject-specific), electronic warnings
- see above

Top risks - review

Review: Lack of review/support for complex prescriptions by (clinical) pharmacists (e.g. high-risk drugs, polypharmacy, complex indications and diagnoses).

How high do you estimate the potential of a solution by strengthening employee competence (e.g. training and further education, etc.) for this risk cluster in the hospital?

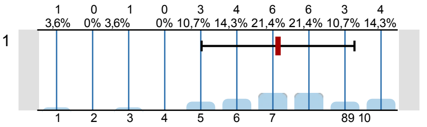

n=28
av.=7.1
dev.=2.1

How high do you estimate the potential of a solution in the area of digitalization (e.g. new software and hardware solutions, etc.) for this risk cluster in the hospital?

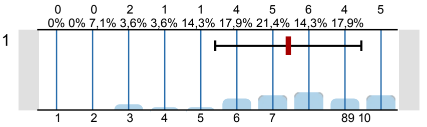

n=28
av.=7.4
dev.=2

Do you have a specific solution proposal for one of the risks mentioned in this risk cluster?

- Automatic query for patients with >5 medications as to whether a clinical pharmacology test should be ordered
- see above, pharmacists should not intervene in the therapy of doctors, there are only very few red flags (e.g. daily MTX intake) for such recommendations a computer/pharmacist would be helpful. However, there are very few serious combination and dosage errors and this is where medical education and training must intervene. Doctors together with other specialists pharmacists... should jointly determine what red flags actually are. Not every small warning is therapeutically relevant.

Top risks - Dispensing

Dispensing: Errors in the preparation/dispensing of medication (e.g. errors in dividing tablets, incorrect medication, incorrect dose, incorrect calculation, missing or incorrect change to the ordered medication in the dispenser, missing or incorrect documentation, missing/incorrect/unclear labeling/labeling of prepared medication).

How high do you estimate the potential of a solution by strengthening employee competence (e.g. training and further education, etc.) for this risk cluster in the hospital?

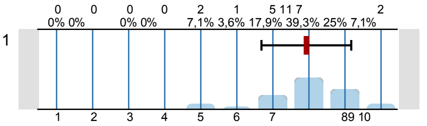

n=28
av.=7.9
dev.=1,2

How high do you estimate the potential of a solution in the area of digitalization (e.g. new software and hardware solutions, etc.) for this risk cluster in the hospital?

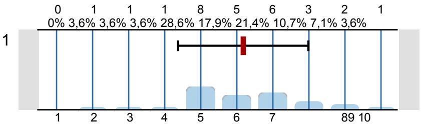

n=28
av.=6.2
dev.=1.8

Do you have a specific solution proposal for one of the risks mentioned in this risk cluster?

- 4 eyes principle and calm environment! However, legible e-fever curves are certainly an advantage.
- Outsource dispensing - medication is dispensed - only changes or new orders need to be dispensed

(error minimization and more time for patient care)

- Sensitization of employees important, electronic help to minimize errors: the electronic temperature curve

Dispensing: Mixing up medications (e.g. mistakes with similar looking medications, mistakes with similar sounding medication names, mixing up medication names/packaging).

How high do you estimate the potential of a solution by strengthening employee competence (e.g. training and further education, etc.) for this risk cluster in the hospital?

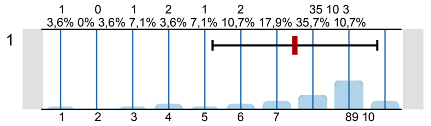

n=28
av.=7.5
dev.=2.3

How high do you estimate the potential of a solution in the area of digitalization (e.g. new software and hardware solutions, etc.) for this risk cluster in the hospital?

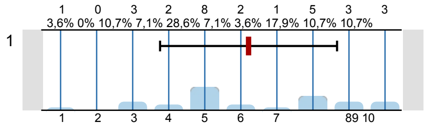

n=28
av.=6.2
dev.=2.5

Do you have a specific solution proposal for one of the risks mentioned in this risk cluster?

- Outsource dispensing - medication is dispensed - only changes or new prescriptions need to be dispensed (minimization of errors and more time for patient care)

- Remove look-a-likes from the product range. Train employees when they see look-a-likes to report/CIRS them immediately before an incident occurs.

- Sensitization of the MA■

Unit Dose

- If all medications are nested using barcode scanners, there will certainly be fewer mix-ups.

Dispensing: Lack of communication/misunderstandings in communication (e.g. errors in telephone instructions, misunderstandings regarding medication names, dosage, interval, dosage form, patient).

How high do you estimate the potential of a solution by strengthening employee competence (e.g. training and further education, etc.) for this risk cluster in the hospital?

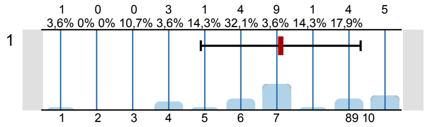

n=28
av.=7.1
dev.=2.2

How high do you estimate the potential of a solution in the area of digitalization (e.g. new software and hardware solutions, etc.) for this risk cluster in the hospital?

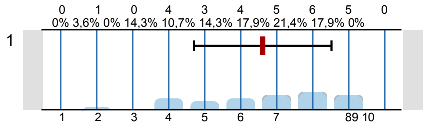

n=28
av.=6.6
dev.=1.9

Do you have a specific solution proposal for one of the risks mentioned in this risk cluster? ■ Sensitization

of employees, release of medication by the doctor (electronic FK)

Top risks - Administration

Administration: medication administration errors (e.g. wrong medication, wrong dosage, wrong route of administration, confusion of similar looking or similar sounding medications, wrong time of administration, unauthorized medications, omission errors, incorrect verification activities, difficulties with infusion equipment, confusion of medication packaging, incorrect labeling of medication on packaging).

How high do you estimate the potential of a solution by strengthening employee competence (e.g. training and further education, etc.) for this risk cluster in the hospital?

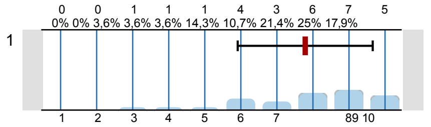

n=28
av.=7.8
dev.=1.9

How high do you estimate the potential of a solution in the area of digitalization (e.g. new software and hardware solutions, etc.) for this risk cluster in the hospital?

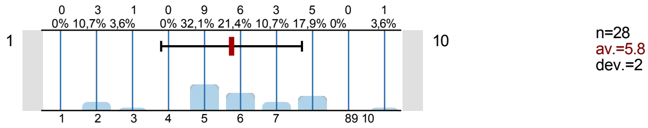

n=28
av.=5.8
dev.=2

Do you have a specific solution proposal for one of the risks mentioned in this risk cluster? ■ Electronic

warning sign for "electronic" overdosing

- Sensitization of employees, confirmation/control eFK
- For infusion therapy: possibly scan patient identification band and infusion label for comparison - correct patient/ correct time/ correct medication
- A barcode scanning system with the wristband could be considered. However, this would probably be perceived as impersonal by patients.

Top risks - Dismissal

Discharge: Lack of communication/information with patients and relatives (e.g. medication requirements, medication not explained).

How high do you estimate the potential of a solution by strengthening employee competence (e.g. training and further education, etc.) for this risk cluster in the hospital?

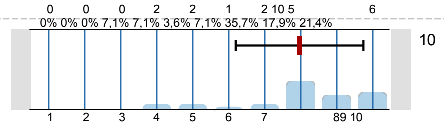

n=28
av.=8
dev.=1.8

How high do you estimate the potential of a solution in the area of digitalization (e.g. new software and hardware solutions, etc.) for this risk cluster in the hospital?

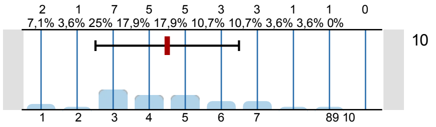

n=28
av.=4.5
dev.=2

Do you have a specific solution proposal for one of the risks mentioned in this risk cluster? ■ Digital

prescription (intramural and extramural)

■ ELGA could be much more helpful if properly filled.

■ Entering doctor's letters in the ELGA. Mark in bold in the doctor's letter if a medication has been newly initiated/terminated/

discontinued■ for special medications such as immunosuppression- Medication training APP for patients to download. Personnel

Top risks - competencies

Competencies: Problematic environment during the individual steps of the medication process (e.g. noise, poor lighting, emergencies, chaotic working environment, interruption/distraction and high workload of staff e.g. due to understaffing, poor equipment of the ward).

How high do you estimate the potential of a solution by strengthening employee competence (e.g. training and further education, etc.) for this risk cluster in the hospital?

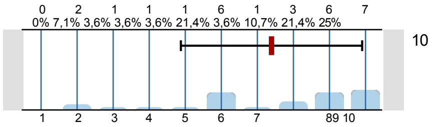

n=28
av.=7.4
dev.=2.5

How high do you estimate the potential of a solution in the area of digitalization (e.g. new software and hardware solutions, etc.) for this risk cluster in the hospital?

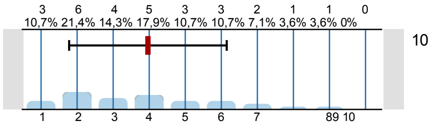

n=28
av.=4
dev.=2.2

Do you have a specific solution proposal for one of the risks mentioned in this risk cluster? ■ Adapt

environment, train/sensitize employees

■ If employees provide medication, they should only be released for this task. I once saw in Germany that employees even wore high-visibility vests... as recognition... It even had something on the back like... "Only speak to me in an emergency, I'm preparing medication"

■ Sharpen knowledge of the factors that promote errors, measures to reduce them (protected space, etc.)

■ more staff. more crisis resource management. Use of checklists and training of employees in the use of checklists.

Top risks - patients and relatives

Patients and relatives: Risk factors relating to patients (e.g. lack of compliance, lack of health literacy, lack of knowledge about their own medication, incorrect use of medication).

How high do you estimate the potential of a solution by strengthening employee competence (e.g. training and further education, etc.) for this risk cluster in the hospital?

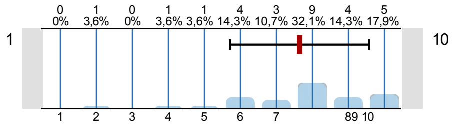

n=28
av.=7.6
dev.=1.9

How high do you estimate the potential of a solution in the area of digitalization (e.g. new software and hardware solutions, etc.) for this risk cluster in the hospital?

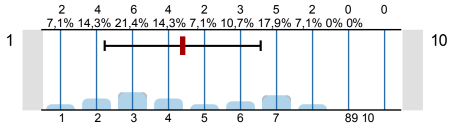

n=28
av.=4.4
dev.=2.2

Do you have a specific solution proposal for one of the risks mentioned in this risk cluster? ■ Clarification

must be provided

■ Information sheets for patients in understandable language. Provide medical staff with printed copies for

their wallets. ■ Communication support for MA patients

■ Finding personal access to the patient (empathy). Finding out what the patient wants and not relying on socially desirable answers.

■ Training MA to be able to carry out good patient education (e.g. useful discussion techniques such as motivational interviewing) Medication APP for special medication groups (e.g. immunos, oral anticoagulation, etc.)

General comments

Is there anything else you would like to tell us?

■ In my opinion, awareness is the key word in this context.

■ In my experience, it would make sense to train external doctors, as patients are often admitted to hospital with very long medication lists, as each doctor prescribes a new medication and the patient is not viewed holistically and attention is not paid to the discontinuation of individual products. It should also not be possible for every doctor to prescribe more specialized medications.

Thank you for your cooperation!
