# Supplementary material for: Risks in the analogue and digitally-supported medication process and potential solutions to increase patient safety in the hospital: A mixed methods study
Source: PLoS One. 2024 Feb 27;19(2):e0297491. doi: 10.1371/journal.pone.0297491 (PMC10898776; doi:10.1371/journal.pone.0297491)
Supplement: Attachment — Submitted filename: Response to Reviewers.docx [file pone.0297491.s006.docx]

**Response to Reviewers:**

We thank the reviewers for their helpful comments. We have incorporated their suggestions in the revised version of the manuscript.

**Reviewer 1:**

**The study's topic sounds interesting. The paper is well written and structured. The methods section is clear and well founded. There are my comments and suggestions:**

**Please remove the three last lines of the introduction since it belongs to the methods section.**

A: We have removed the last two sentences of the introduction and have added additional information in the methods section where appropriate.

**Please add a flowchart to summarize the literature search process. This will be appreciated by lecturers.**

A: We would like to thank the reviewer for this valuable comment. We agree with the suggestion and have added a flow chart to provide a better overview of the structured literature search.

**Please give more details about how the selected bibliography was considered as appropriate. How the reviewers validate the final selected references?**

A: As already described in the manuscript in the patients, materials and methods section in the paragraph about the literature search in phase 1 – identification of main risk clusters on page 4, we have limited our search to systematic reviews in English. Reviews concerning only critically ill patients, pediatrics, emergency department, or outpatient clinic were excluded. However, we included systematic reviews that included studies conducted in different settings, such as general inpatient wards and pediatric wards and/or intensive care units. Further, systematic reviews that investigated the whole medication use process and thus provided relevant information to answer the research question were included rather than systematic reviews that investigated only one aspect of the medication use process. Additional information has been added to the manuscript as well.

**Can the authors describe the two investigated processes (analogue and the digital medication use process) and give, if exist, differences between the two processes**

A: We thank the reviewer for this important comment. The analogue medication process consists of complete or partial work steps and processes that do not use IT tools. This can mean that the analogue process is for example initiated with an electronic prescription-tool for the medication, but the medication is dispensed and distributed manually and does not receive further digital support. A completely digitally supported process is therefore the goal, which is initialized with the electronic prescription, followed by machine-supported medication dispensing through to secure, barcode-supported medication distribution to patients. This so-called closed-loop-principle is a goal in order to minimize or even prevent any medication error in means of that the right medication is prescribed, dispensed and distributed to the right patient.

**Please give more details about how the experts were selected for both phases of the study (number and quality).**

A: We approached 75 experts from the DACH region, including Germany, Switzerland and Austria, to participate in the questionnaires based on the Delphi method. The experts had different backgrounds, but all focusing on different research fields in clinical and patient-related areas. The final selection was based on the responses and the willingness of the experts to take part in the questionnaires.

We provided these details about the expert selection process in chapter Materials and Methods.

**Line 114-116: please reword to underline that two parameters were considered each one rated on a 10-point scale.**

A: We thank the reviewer for reading our manuscript in such detail and for the valuable suggestion. We have rephrased the sentence that describes the Likert scale used in questionnaire A accordingly.

**Why the authors did not consider multiplying the scores of the two parameters to have a more comprehensive decision?**

A: We thank the reviewer for this valuable comment. We had internal discussions on how to best rank the identified results concerning likelihood of occurrence and impact on patient safety of the risk clusters. However, we agreed to add the mean of both parameters and to not multiply them, in order to give equal weight to both parameters.

**Please justify why only the 15 top risks were considered.**

A: At a certain stage in the project, we agreed to narrow down the identified 43 risk clusters to the most important ones. We performed the ranking as described in the manuscript in the patients, materials and methods section in the paragraph about questionnaire A in phase 2 – likelihood of risk occurrence, impact on patient safety and potential for solutions.

**Delphi method looks for consensus. What are the consensus criteria considered by the authors? For example, the 14th risk (table 2) had just 5.4 on 10-point scale while other risks have higher scores.**

A: We thank the reviewer for this valuable comment. We want to clarify at that point, that we have not performed a typical Delphi study, but rather conducted two questionnaires that were based on the Delphi method. Thus, we asked experts to evaluate the risk clusters on a Likert scale ranging from 1-10.

**Only 44% of the selected experts respond to the questionnaires. May this impact the representativeness?**

A: The questionnaires were already sent to a targeted population of experts in the clinical and patient-related fields from the DACH region and we consider the response rate of 44% and 37% high compared to other surveys.

**Reviewer 2:**

**Thank you for the opportunity to review the manuscript on an important medication safety topic. I would suggest to improve the legibility of the manuscript to consider it being reviewed by an English language editing service. Additionally, I would suggest expanding section on implication on practice and research as well as the study limitations.**

A: We thank the reviewer for these valuable comments. We have let the manuscript being reviewed by an academic English native speaker and have added changes regarding grammar and wording in the manuscript. Further, we have expanded the discussion section on limitations and implications for the practice accordingly by adding this paragraph: ‘Another limitation is the targeted expert group that was approached to participate in the two questionnaires based on the Delphi method. Since experts were from the DACH region, the results must be interpreted carefully when comparing with other regions, taking differences in health care systems into account. Nevertheless, the implication for clinical practice is given through by raising awareness of the risk clusters as well as providing a list of possible solutions.’
