# Supplementary material for: Risks in the analogue and digitally-supported medication process and potential solutions to increase patient safety in the hospital: A mixed methods study
Source: PLoS One. 2024 Feb 27;19(2):e0297491. doi: 10.1371/journal.pone.0297491 (PMC10898776; doi:10.1371/journal.pone.0297491)
Supplement: Peer review history

# Peer review: Decision Letter 0

**DOI:** 10.1371/journal.pone.0297491.r001

**Authors:** Ramune Jacobsen

12 Sep 2023

PONE-D-23-20698Burden of risks in the analogue and digitally-supported medication use process and potential for solutions to increase patient safety in the hospital: a mixed method studyPLOS ONE

Dear Dr. Lichtenegger,

Thank you for submitting your manuscript to PLOS ONE. After careful consideration, we feel that it has merit but does not fully meet PLOS ONE’s publication criteria as it currently stands. Therefore, we invite you to submit a revised version of the manuscript that addresses the points raised during the review process.

Please submit your revised manuscript by Oct 27 2023 11:59PM. If you will need more time than this to complete your revisions, please reply to this message or contact the journal office at plosone@plos.org. When you're ready to submit your revision, log on to https://www.editorialmanager.com/pone/ and select the 'Submissions Needing Revision' folder to locate your manuscript file.

Please include the following items when submitting your revised manuscript:

- A rebuttal letter that responds to each point raised by the academic editor and reviewer(s). You should upload this letter as a separate file labeled 'Response to Reviewers'.
- A marked-up copy of your manuscript that highlights changes made to the original version. You should upload this as a separate file labeled 'Revised Manuscript with Track Changes'.
- An unmarked version of your revised paper without tracked changes. You should upload this as a separate file labeled 'Manuscript'.

If you would like to make changes to your financial disclosure, please include your updated statement in your cover letter. Guidelines for resubmitting your figure files are available below the reviewer comments at the end of this letter.

If applicable, we recommend that you deposit your laboratory protocols in protocols.io to enhance the reproducibility of your results. Protocols.io assigns your protocol its own identifier (DOI) so that it can be cited independently in the future. For instructions see: https://journals.plos.org/plosone/s/submission-guidelines#loc-laboratory-protocols. Additionally, PLOS ONE offers an option for publishing peer-reviewed Lab Protocol articles, which describe protocols hosted on protocols.io. Read more information on sharing protocols at https://plos.org/protocols?utm_medium=editorial-email&utm_source=authorletters&utm_campaign=protocols.

We look forward to receiving your revised manuscript.

Kind regards,

Ramune Jacobsen

Academic Editor

PLOS ONE

Journal requirements:

When submitting your revision, we need you to address these additional requirements.

1. Please ensure that your manuscript meets PLOS ONE's style requirements, including those for file naming. The PLOS ONE style templates can be found at

https://journals.plos.org/plosone/s/file?id=wjVg/PLOSOne_formatting_sample_main_body.pdf and

https://journals.plos.org/plosone/s/file?id=ba62/PLOSOne_formatting_sample_title_authors_affiliations.pdf

2. Please provide additional details regarding participant consent. In the ethics statement in the Methods and online submission information, please ensure that you have specified (1) whether consent was informed and (2) what type you obtained (for instance, written or verbal, and if verbal, how it was documented and witnessed). If your study included minors, state whether you obtained consent from parents or guardians. If the need for consent was waived by the ethics committee, please include this information

If you are reporting a retrospective study of medical records or archived samples, please ensure that you have discussed whether all data were fully anonymized before you accessed them and/or whether the IRB or ethics committee waived the requirement for informed consent. If patients provided informed written consent to have data from their medical records used in research, please include this information

Once you have amended this/these statement(s) in the Methods section of the manuscript, please add the same text to the “Ethics Statement” field of the submission form (via “Edit Submission”).

For additional information about PLOS ONE ethical requirements for human subjects research, please refer to http://journals.plos.org/plosone/s/submission-guidelines#loc-human-subjects-research.

3. We note that you have indicated that data from this study are available upon request. PLOS only allows data to be available upon request if there are legal or ethical restrictions on sharing data publicly. For more information on unacceptable data access restrictions, please see http://journals.plos.org/plosone/s/data-availability#loc-unacceptable-data-access-restrictions.

In your revised cover letter, please address the following prompts:

a) If there are ethical or legal restrictions on sharing a de-identified data set, please explain them in detail (e.g., data contain potentially sensitive information, data are owned by a third-party organization, etc.) and who has imposed them (e.g., an ethics committee). Please also provide contact information for a data access committee, ethics committee, or other institutional body to which data requests may be sent.

b) If there are no restrictions, please upload the minimal anonymized data set necessary to replicate your study findings as either Supporting Information files or to a stable, public repository and provide us with the relevant URLs, DOIs, or accession numbers. For a list of acceptable repositories, please see http://journals.plos.org/plosone/s/data-availability#loc-recommended-repositories.

We will update your Data Availability statement on your behalf to reflect the information you provide.

4. PLOS requires an ORCID iD for the corresponding author in Editorial Manager on papers submitted after December 6th, 2016. Please ensure that you have an ORCID iD and that it is validated in Editorial Manager. To do this, go to ‘Update my Information’ (in the upper left-hand corner of the main menu), and click on the Fetch/Validate link next to the ORCID field. This will take you to the ORCID site and allow you to create a new iD or authenticate a pre-existing iD in Editorial Manager. Please see the following video for instructions on linking an ORCID iD to your Editorial Manager account: https://www.youtube.com/watch?v=_xcclfuvtxQ

5. Please review your reference list to ensure that it is complete and correct. If you have cited papers that have been retracted, please include the rationale for doing so in the manuscript text, or remove these references and replace them with relevant current references. Any changes to the reference list should be mentioned in the rebuttal letter that accompanies your revised manuscript. If you need to cite a retracted article, indicate the article’s retracted status in the References list and also include a citation and full reference for the retraction notice.

[Note: HTML markup is below. Please do not edit.]

Reviewers' comments:

Reviewer's Responses to Questions

**Comments to the Author**

1. Is the manuscript technically sound, and do the data support the conclusions?

The manuscript must describe a technically sound piece of scientific research with data that supports the conclusions. Experiments must have been conducted rigorously, with appropriate controls, replication, and sample sizes. The conclusions must be drawn appropriately based on the data presented. 

Reviewer #1: Yes

Reviewer #2: Yes

**********

2. Has the statistical analysis been performed appropriately and rigorously? 

Reviewer #1: Yes

Reviewer #2: Yes

**********

3. Have the authors made all data underlying the findings in their manuscript fully available?

The PLOS Data policy requires authors to make all data underlying the findings described in their manuscript fully available without restriction, with rare exception (please refer to the Data Availability Statement in the manuscript PDF file). The data should be provided as part of the manuscript or its supporting information, or deposited to a public repository. For example, in addition to summary statistics, the data points behind means, medians and variance measures should be available. If there are restrictions on publicly sharing data—e.g. participant privacy or use of data from a third party—those must be specified.

Reviewer #1: Yes

Reviewer #2: Yes

**********

4. Is the manuscript presented in an intelligible fashion and written in standard English?

PLOS ONE does not copyedit accepted manuscripts, so the language in submitted articles must be clear, correct, and unambiguous. Any typographical or grammatical errors should be corrected at revision, so please note any specific errors here.

Reviewer #1: Yes

Reviewer #2: No

**********

5. Review Comments to the Author

Please use the space provided to explain your answers to the questions above. You may also include additional comments for the author, including concerns about dual publication, research ethics, or publication ethics. (Please upload your review as an attachment if it exceeds 20,000 characters)

Reviewer #1: The study's topic sounds interesting. The paper is well written and structured. The methods section is clear and well founded.

There are my comments and suggestions:

- please remove the three last lines of the introduction since it belongs to the methods section.

- please add a flowchart to summarize the literature search process. This will be appreciated by lecturers.

- please give more details about how the selected bibliography was considered as appropriate. How the reviewers validate the final selected references?

- can the authors describe the two investigated processes (analogue and the digital medication use process) and give, if exist, differences between the two processes

- please give more details about how the experts were selected for both phases of the study (number and quality).

- line 114-116: please reword to underline that two parameters were considered each one rated on a 10-point scale.

- why the authors did not consider multiplying the scores of the two parameters to have a more comprehensive decision?

- please justify why only the 15 top risks were considered.

- Delphi method looks for consensus. What are the consensus criteria considered by the authors? For example, the 14th risk (table 2) had just 5.4 on 10-point scale while other risks have higher scores.

- Only 44% of the selected experts respond to the questionnaires. May this impact the representativeness?

Reviewer #2: Thank you for the opportunity to review the manuscript on an important medication safety topic. I would suggest to improve the legibility of the manuscript to consider it being reviewed by an English language editing service . Additionally, I would suggest expanding section on implication on practice and research as well as the study limitations.

**********

6. PLOS authors have the option to publish the peer review history of their article (what does this mean?). If published, this will include your full peer review and any attached files.

If you choose “no”, your identity will remain anonymous but your review may still be made public.

**Do you want your identity to be public for this peer review?** For information about this choice, including consent withdrawal, please see our Privacy Policy.

Reviewer #1: **Yes: **Aïmen Abbassi

Reviewer #2: No

**********

[NOTE: If reviewer comments were submitted as an attachment file, they will be attached to this email and accessible via the submission site. Please log into your account, locate the manuscript record, and check for the action link "View Attachments". If this link does not appear, there are no attachment files.]

While revising your submission, please upload your figure files to the Preflight Analysis and Conversion Engine (PACE) digital diagnostic tool, https://pacev2.apexcovantage.com/. PACE helps ensure that figures meet PLOS requirements. To use PACE, you must first register as a user. Registration is free. Then, login and navigate to the UPLOAD tab, where you will find detailed instructions on how to use the tool. If you encounter any issues or have any questions when using PACE, please email PLOS at figures@plos.org. Please note that Supporting Information files do not need this step.

# Author comment: Author response to Decision Letter 0

**DOI:** 10.1371/journal.pone.0297491.r002

4 Dec 2023

Response to Reviewers:

We thank the reviewers for their helpful comments. We have incorporated their suggestions in the revised version of the manuscript.

Reviewer 1:

The study's topic sounds interesting. The paper is well written and structured. The methods section is clear and well founded. There are my comments and suggestions:

Please remove the three last lines of the introduction since it belongs to the methods section.

A: We have removed the last two sentences of the introduction and have added additional information in the methods section where appropriate.

Please add a flowchart to summarize the literature search process. This will be appreciated by lecturers.

A: We would like to thank the reviewer for this valuable comment. We agree with the suggestion and have added a flow chart to provide a better overview of the structured literature search.

Please give more details about how the selected bibliography was considered as appropriate. How the reviewers validate the final selected references?

A: As already described in the manuscript in the patients, materials and methods section in the paragraph about the literature search in phase 1 – identification of main risk clusters on page 4, we have limited our search to systematic reviews in English. Reviews concerning only critically ill patients, pediatrics, emergency department, or outpatient clinic were excluded. However, we included systematic reviews that included studies conducted in different settings, such as general inpatient wards and pediatric wards and/or intensive care units. Further, systematic reviews that investigated the whole medication use process and thus provided relevant information to answer the research question were included rather than systematic reviews that investigated only one aspect of the medication use process. Additional information has been added to the manuscript as well.

Can the authors describe the two investigated processes (analogue and the digital medication use process) and give, if exist, differences between the two processes

A: We thank the reviewer for this important comment. The analogue medication process consists of complete or partial work steps and processes that do not use IT tools. This can mean that the analogue process is for example initiated with an electronic prescription-tool for the medication, but the medication is dispensed and distributed manually and does not receive further digital support. A completely digitally supported process is therefore the goal, which is initialized with the electronic prescription, followed by machine-supported medication dispensing through to secure, barcode-supported medication distribution to patients. This so-called closed-loop-principle is a goal in order to minimize or even prevent any medication error in means of that the right medication is prescribed, dispensed and distributed to the right patient.

Please give more details about how the experts were selected for both phases of the study (number and quality).

A: We approached 75 experts from the DACH region, including Germany, Switzerland and Austria, to participate in the questionnaires based on the Delphi method. The experts had different backgrounds, but all focusing on different research fields in clinical and patient-related areas. The final selection was based on the responses and the willingness of the experts to take part in the questionnaires.

We provided these details about the expert selection process in chapter Materials and Methods.

Line 114-116: please reword to underline that two parameters were considered each one rated on a 10-point scale.

A: We thank the reviewer for reading our manuscript in such detail and for the valuable suggestion. We have rephrased the sentence that describes the Likert scale used in questionnaire A accordingly.

Why the authors did not consider multiplying the scores of the two parameters to have a more comprehensive decision?

A: We thank the reviewer for this valuable comment. We had internal discussions on how to best rank the identified results concerning likelihood of occurrence and impact on patient safety of the risk clusters. However, we agreed to add the mean of both parameters and to not multiply them, in order to give equal weight to both parameters.

Please justify why only the 15 top risks were considered.

A: At a certain stage in the project, we agreed to narrow down the identified 43 risk clusters to the most important ones. We performed the ranking as described in the manuscript in the patients, materials and methods section in the paragraph about questionnaire A in phase 2 – likelihood of risk occurrence, impact on patient safety and potential for solutions.

Delphi method looks for consensus. What are the consensus criteria considered by the authors? For example, the 14th risk (table 2) had just 5.4 on 10-point scale while other risks have higher scores.

A: We thank the reviewer for this valuable comment. We want to clarify at that point, that we have not performed a typical Delphi study, but rather conducted two questionnaires that were based on the Delphi method. Thus, we asked experts to evaluate the risk clusters on a Likert scale ranging from 1-10.

Only 44% of the selected experts respond to the questionnaires. May this impact the representativeness?

A: The questionnaires were already sent to a targeted population of experts in the clinical and patient-related fields from the DACH region and we consider the response rate of 44% and 37% high compared to other surveys.

Reviewer 2: 

Thank you for the opportunity to review the manuscript on an important medication safety topic. I would suggest to improve the legibility of the manuscript to consider it being reviewed by an English language editing service. Additionally, I would suggest expanding section on implication on practice and research as well as the study limitations.

A: We thank the reviewer for these valuable comments. We have let the manuscript being reviewed by an academic English native speaker and have added changes regarding grammar and wording in the manuscript. Further, we have expanded the discussion section on limitations and implications for the practice accordingly by adding this paragraph: ‘Another limitation is the targeted expert group that was approached to participate in the two questionnaires based on the Delphi method. Since experts were from the DACH region, the results must be interpreted carefully when comparing with other regions, taking differences in health care systems into account. Nevertheless, the implication for clinical practice is given through by raising awareness of the risk clusters as well as providing a list of possible solutions.’

Attachment Submitted filename: Response to Reviewers.docx

# Peer review: Decision Letter 1

**DOI:** 10.1371/journal.pone.0297491.r003

**Authors:** Ramune Jacobsen

8 Jan 2024

Risks in the analogue and digitally-supported medication process and potential solutions to increase patient safety in the hospital: a mixed method study

PONE-D-23-20698R1

Dear Dr. Lichtenegger,

We’re pleased to inform you that your manuscript has been judged scientifically suitable for publication and will be formally accepted for publication once it meets all outstanding technical requirements.

Within one week, you’ll receive an e-mail detailing the required amendments. When these have been addressed, you’ll receive a formal acceptance letter and your manuscript will be scheduled for publication.

An invoice for payment will follow shortly after the formal acceptance. To ensure an efficient process, please log into Editorial Manager at http://www.editorialmanager.com/pone/, click the 'Update My Information' link at the top of the page, and double check that your user information is up-to-date. If you have any billing related questions, please contact our Author Billing department directly at authorbilling@plos.org.

If your institution or institutions have a press office, please notify them about your upcoming paper to help maximize its impact. If they’ll be preparing press materials, please inform our press team as soon as possible -- no later than 48 hours after receiving the formal acceptance. Your manuscript will remain under strict press embargo until 2 pm Eastern Time on the date of publication. For more information, please contact onepress@plos.org.

Kind regards,

Ramune Jacobsen

Academic Editor

PLOS ONE

Reviewers' comments:

Reviewer's Responses to Questions

**Comments to the Author**

1. If the authors have adequately addressed your comments raised in a previous round of review and you feel that this manuscript is now acceptable for publication, you may indicate that here to bypass the “Comments to the Author” section, enter your conflict of interest statement in the “Confidential to Editor” section, and submit your "Accept" recommendation.

Reviewer #1: All comments have been addressed

Reviewer #2: All comments have been addressed

**********

2. Is the manuscript technically sound, and do the data support the conclusions?

The manuscript must describe a technically sound piece of scientific research with data that supports the conclusions. Experiments must have been conducted rigorously, with appropriate controls, replication, and sample sizes. The conclusions must be drawn appropriately based on the data presented. 

Reviewer #1: Yes

Reviewer #2: Yes

**********

3. Has the statistical analysis been performed appropriately and rigorously? 

Reviewer #1: Yes

Reviewer #2: Yes

**********

4. Have the authors made all data underlying the findings in their manuscript fully available?

The PLOS Data policy requires authors to make all data underlying the findings described in their manuscript fully available without restriction, with rare exception (please refer to the Data Availability Statement in the manuscript PDF file). The data should be provided as part of the manuscript or its supporting information, or deposited to a public repository. For example, in addition to summary statistics, the data points behind means, medians and variance measures should be available. If there are restrictions on publicly sharing data—e.g. participant privacy or use of data from a third party—those must be specified.

Reviewer #1: Yes

Reviewer #2: Yes

**********

5. Is the manuscript presented in an intelligible fashion and written in standard English?

PLOS ONE does not copyedit accepted manuscripts, so the language in submitted articles must be clear, correct, and unambiguous. Any typographical or grammatical errors should be corrected at revision, so please note any specific errors here.

Reviewer #1: Yes

Reviewer #2: Yes

**********

6. Review Comments to the Author

Please use the space provided to explain your answers to the questions above. You may also include additional comments for the author, including concerns about dual publication, research ethics, or publication ethics. (Please upload your review as an attachment if it exceeds 20,000 characters)

Reviewer #1: (No Response)

Reviewer #2: Thanks for considering the comments presented to the authors for considerations. Most comments have been addressed .

**********

7. PLOS authors have the option to publish the peer review history of their article (what does this mean?). If published, this will include your full peer review and any attached files.

If you choose “no”, your identity will remain anonymous but your review may still be made public.

**Do you want your identity to be public for this peer review?** For information about this choice, including consent withdrawal, please see our Privacy Policy.

Reviewer #1: **Yes: **Aïmen Abbassi

Reviewer #2: No

**********

# Editor report: Acceptance letter

**DOI:** 10.1371/journal.pone.0297491.r004

**Authors:** Ramune Jacobsen

17 Feb 2024

PONE-D-23-20698R1 

PLOS ONE

Dear Dr. Lichtenegger, 

I'm pleased to inform you that your manuscript has been deemed suitable for publication in PLOS ONE. Congratulations! Your manuscript is now being handed over to our production team.

At this stage, our production department will prepare your paper for publication. This includes ensuring the following:

* All references, tables, and figures are properly cited

* All relevant supporting information is included in the manuscript submission,

* There are no issues that prevent the paper from being properly typeset

If revisions are needed, the production department will contact you directly to resolve them. If no revisions are needed, you will receive an email when the publication date has been set. At this time, we do not offer pre-publication proofs to authors during production of the accepted work. Please keep in mind that we are working through a large volume of accepted articles, so please give us a few weeks to review your paper and let you know the next and final steps. 

Lastly, if your institution or institutions have a press office, please let them know about your upcoming paper now to help maximize its impact. If they'll be preparing press materials, please inform our press team within the next 48 hours. Your manuscript will remain under strict press embargo until 2 pm Eastern Time on the date of publication. For more information, please contact onepress@plos.org.

If we can help with anything else, please email us at customercare@plos.org.

Thank you for submitting your work to PLOS ONE and supporting open access. 

Kind regards, 

PLOS ONE Editorial Office Staff

on behalf of

Dr. Ramune Jacobsen 

Academic Editor

PLOS ONE
